# Long-distance device-independent quantum key distribution

**DOI:** 10.1038/s41598-019-53803-0

**Published:** 2019-11-28

**Authors:** Víctor Zapatero, Marcos Curty

**Affiliations:** Escuela de Ingeniería de Telecomunicación, Department of Signal Theory and Communications, University of Vigo, Vigo, E-36310 Spain

**Keywords:** Quantum information, Quantum optics

## Abstract

Besides being a beautiful idea, device-independent quantum key distribution (DIQKD) is probably the ultimate solution to defeat quantum hacking. Its security is based on a loophole-free violation of a Bell inequality, which results in a very limited maximum achievable distance. To overcome this limitation, DIQKD must be furnished with heralding devices like, for instance, qubit amplifiers, which can signal the arrival of a photon before the measurement settings are actually selected. In this way, one can decouple channel loss from the selection of the measurement settings and, consequently, it is possible to safely post-select the heralded events and discard the rest, which results in a significant enhancement of the achievable distance. In this work, we investigate photonic-based DIQKD assisted by two main types of qubit amplifiers in the finite data block size scenario, and study the resources—particularly, the detection efficiency of the photodetectors and the quality of the entanglement sources—that would be necessary to achieve long-distance DIQKD within a reasonable time frame of signal transmission.

## Introduction

The use of quantum mechanics for cryptographic means was first proposed in the early 70’s by Stephen Wiesner, aiming to create unfalsifiable banknotes^1^. Inspired by this seminal work, Charles Bennett and Gilles Brassard introduced a protocol to securely distribute cryptographic keys^2^. Nowadays, intense theoretical and experimental research^3–5^ has turned this latter task—called quantum key distribution (QKD)—into a feasible commercial solution^6^.

Despite such tremendous progress, a major flaw of QKD today is the existing big gap between the theory and the practice. This is so because security proofs of QKD typically rely on simple mathematical models to describe the behaviour of the different physical devices. As a result, any departure from these models might render real-life QKD implementations vulnerable to quantum hacking attacks^7–13^.

To overcome this problem, the ultimate solution probably is device-independent QKD (DIQKD)^14–19^. Given that the users’ devices are honest^20,21^, DIQKD can guarantee security without characterizing the internal functioning of the apparatuses, thereby ruling out all hacking attacks against the physical implementation. It is based on a feature of some entangled states known as nonlocality^22^, which guarantees that two distant parties (say, Alice and Bob) sharing an ideal nonlocal quantum state observe perfectly correlated outcomes when performing adequate quantum measurements on their shares. Moreover, these correlations are monogamous, *i.e*., the measurement outcomes are statistically independent of any pre-existing information held by a third party. This property can be verified with a two-party Bell test^22–27^ known as the Clauser-Horne-Shimony-Holt (CHSH) test, which basically consists of repeatedly playing a two-party nonlocal game^19,28^. The winning rate of the game indicates the amount of monogamous correlations shared between Alice and Bob.

The security of DIQKD has been rigorously established in different works, first against collective attacks^15^ in the asymptotic regime, then against coherent attacks^17^ also in the asymptotic regime, and only recently in the practical scenario of finite data block sizes^28^ (see also^29^). The security proof in^28^ relies on the so-called entropy accumulation theorem^30,31^, which effectively allows to prove the security of the full protocol from the security of a single round of the protocol by using a worst-case scenario.

Security proofs require, however, that two fundamental loopholes are closed: the *locality loophole*^22^ and the *detection loophole*^22,32,33^. The former is closed by enforcing a proper isolation of Alice’s and Bob’s devices. Closing the detection loophole is more tricky, especially if Alice and Bob wish to cover long distances. Indeed, if an adversary were able to correlate channel loss to Alice’s or Bob’s measurement settings, such an adversary could easily fake nonlocal correlations, and thus compromise the security of the distilled key. A simple solution is to assign a pre-established outcome value to each lost signal while running the CHSH test. The main drawback of this technique is however the limited achievable distance, because such mapping translates loss into errors. Indeed, with such an approach, even if the entanglement source could generate perfect Bell pairs and Alice and Bob could measure them with unit efficiency detectors, channel loss would limit the maximum DIQKD transmission distance to about only 3.5 km for a typical optical fiber in the telecom wavelength with an attenuation coefficient equal to $\alpha =0.2$ dB/km.

To enhance the distance, Alice and Bob need to use heralding devices. These are devices that herald the arrival of a signal to the receiver, allowing a fair post-selection of the heralded events. Then, if Alice and Bob choose their measurement settings *a posteriori*, *i.e*., once their heralding devices have declared the reception of a signal, they actually decouple channel loss from their measurement settings selection, thus paving the way for DIQKD over long distances.

A heralding device particularly suited for DIQKD is a qubit amplifier^34–36^, which basically consists of a teleportation gate. That is, a successful heralding corresponds to the teleportation of the state of the arriving signal to a signal at the output port of the qubit amplifier. DIQKD supported by qubit amplifiers has been analysed in^34–38^ in the asymptotic regime, *i.e*., by considering an infinite number of signals. In this work, we focus on the practical finite data block size scenario. More precisely, we study finite-key DIQKD with the two different types of qubit amplifier architectures introduced in^35^ and^36^. We pay particular attention to the effect that typical device imperfections (especially, the finite detection efficiency of the photodetectors and the multi-photon pulses emitted by practical entanglement light sources) have on the performance of the system. In doing so, we determine the resources needed to achieve long-distance implementations of DIQKD within a reasonable time frame of signal transmission.

## Results

### DIQKD protocol

We consider the DIQKD protocol introduced in^28^. It is based on a CHSH test^24^, and it is equivalent to a certain two-party nonlocal game known as the CHSH game.

Before presenting the steps of the protocol in detail, let us introduce some notation first. Alice’s measurement setting in the $i$-th successfully heralded round of the protocol is denoted by ${X}_{i}\in \{0,1\}$, where ${X}_{i}=0$ and ${X}_{i}=1$ tag the measurements described by the two following Pauli operators

1 $${\sigma }_{{\rm{z}}}=(\begin{array}{cc}1 & 0\\ 0 & -1\end{array})\,\mathrm{and}\,{\sigma }_{{\rm{x}}}=(\begin{array}{cc}0 & 1\\ 1 & 0\end{array}),$$

respectively. On the other hand, Bob’s measurement setting in the $i$-th successfully heralded round of the protocol is denoted by ${Y}_{i}\in \{0,1,2\}$, where ${Y}_{i}=0$ tags the measurement ${\sigma }_{+}=({\sigma }_{{\rm{z}}}+{\sigma }_{{\rm{x}}})/\sqrt{2}$, ${Y}_{i}=1$ indicates the measurement ${\sigma }_{-}=({\sigma }_{{\rm{z}}}-{\sigma }_{{\rm{x}}})/\sqrt{2}$ and ${Y}_{i}=2$ refers to the measurement ${\sigma }_{{\rm{z}}}$, with ${\sigma }_{{\rm{z}}}$ and ${\sigma }_{{\rm{x}}}$ again given by Eq. (1). Similarly, Alice’s (Bob’s) outcome in the $i$-th successfully heralded round is denoted by ${A}_{i}\in \{0,1\}$ (${B}_{i}\in \{0,1\}$).

**Figure 1:**
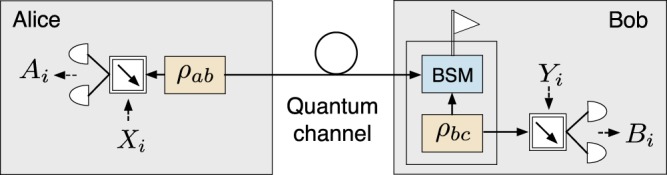
Schematic of the considered DIQKD protocol. While Alice holds an entanglement source, ${\rho }_{ab}$, in her lab, Bob holds a qubit amplifier, which consists of an entanglement source, ${\rho }_{bc}$, and a Bell state measurement (BSM) used for teleportation. The role of the qubit amplifier is to mitigate the effect of channel loss. In every round of the protocol in which a successful heralding takes place at the qubit amplifier, Bob randomly chooses a bit value ${T}_{i}\in \{0,1\}$. If ${T}_{i}=0$, Alice (Bob) chooses as measurement setting ${X}_{i}={\sigma }_{{\rm{z}}}$ (${Y}_{i}={\sigma }_{{\rm{z}}}$). If ${T}_{i}=1$, Alice chooses at random her measurement setting ${X}_{i}\in \{{\sigma }_{{\rm{z}}},{\sigma }_{{\rm{x}}}\}$, with ${\sigma }_{{\rm{z}}}$ and ${\sigma }_{{\rm{x}}}$ being the Pauli matrices given by Eq. (1). Similarly, in this latter case, Bob chooses at random his measurement setting ${Y}_{i}\in \left\{{\sigma }_{+},{\sigma }_{-}\right\}$, where ${\sigma }_{\pm }=({\sigma }_{{\rm{z}}}\pm {\sigma }_{{\rm{x}}})/\sqrt{2}$. Their respective outcomes are recorded as ${A}_{i},{B}_{i}\in \left\{0,1\right\}$, where ${A}_{i}$ (${B}_{i}$) indicates which of Alice’s (Bob’s) two photodetectors registered a single-photon pulse. If, say, Alice obtains an inconclusive result (*i.e*., no photons or multiple photons are observed, she deterministically selects ${A}_{i}=1$, and similarly for Bob. The reader is referred to the main text for further details.

Next, we present the different steps of the protocol. A schematic is shown in Fig. 1. For simplicity, we shall assume here that only Bob holds a qubit amplifier to compensate channel loss (see the Methods section for a description of the qubit amplifiers we consider), while Alice has the entanglement source, ${\rho }_{ab}$, in her lab. The case where both Alice and Bob hold a qubit amplifier and the entanglement source is located in the middle of the channel between them is analyzed in the Supplementary Information.

**Protocol steps**

1. *Initialization*. Bob sets the counter $i$ of successfully heralded rounds to 0. While $i < {n}_{{\rm{S}}{\rm{H}}}$ for a certain prefixed value ${n}_{{\rm{S}}{\rm{H}}}$, steps $2$ and $3$ below are repeated.
2. *Distribution*. Alice prepares a bipartite entangled state, ${\rho }_{ab}$, and sends system $B$ to Bob through the quantum channel. If no successful heralding takes place at Bob’s qubit amplifier, the signal is discarded and step 2 is repeated. Otherwise, Bob updates the counter $i$ to $i+1$. Then, he randomly chooses a bit value ${T}_{i}\in \left\{0,1\right\}$ with probabilities $P({T}_{i}=0)=1-\gamma$ and $P({T}_{i}=1)=\gamma$, respectively, and sends it to Alice through an authenticated classical channel. We denote by $T=({T}_{1},{T}_{2},\ldots ,{T}_{{n}_{{\rm{S}}{\rm{H}}}})$ the string of all bit values ${T}_{i}$.
3. *Measurement*. If ${T}_{i}=0$, the $i$-th successfully heralded round is considered to be a key generation round, and Alice and Bob choose the settings $({X}_{i},{Y}_{i})=(0,2)$. If ${T}_{i}=1$, such round is considered to be a test round (*i.e*., a CHSH game round), and they independently select ${X}_{i},{Y}_{i}\in \left\{0,1\right\}$ uniformly at random. Alice and Bob record their measurement outcomes as ${A}_{i}$, ${B}_{i}\in \{0,1\}$, respectively. If, say, Alice (Bob) obtains an inconclusive result (*i.e*., no photon or multiple photons are observed in the detectors) she (he) deterministically assigns ${A}_{i}=1$ (${B}_{i}=1$) to keep the detection loophole closed. Finally, Alice (Bob) publicly announces the measurement settings ${X}_{i}$ (${Y}_{i}$). In what follows, we will denote by $X$ ($Y$) the bit string ${X}_{1},{X}_{2},\ldots ,{X}_{{n}_{{\rm{S}}{\rm{H}}}}$ (${Y}_{1},{Y}_{2},\ldots ,{Y}_{{n}_{{\rm{S}}{\rm{H}}}}$) of Alice’s (Bob’s) measurement settings for the successfully heralded rounds. Similarly, $a$ ($B$) will denote the string of measurement outcomes ${A}_{1},{A}_{2},\ldots ,{A}_{{n}_{{\rm{S}}{\rm{H}}}}$ (${B}_{1},{B}_{2},\ldots ,{B}_{{n}_{{\rm{S}}{\rm{H}}}}$).
4. *Information reconciliation*. Alice and Bob use an error correction protocol to obtain two identical bit strings, ${Z}_{{\rm{A}}}$ and ${Z}_{{\rm{B}}}$, from $A$ and $B$, respectively. For this, Alice sends Bob $lea{k}_{{\rm{I}}{\rm{R}}}$ bits of syndrome information and Bob obtains an estimate, ${Z}_{{\rm{B}}}$, of $A$. Next, they perform an error verification step (using two-universal hash functions) that leaks at most $\lceil {\log }_{2}(1/{\epsilon }_{{\rm{I}}{\rm{R}}})\rceil$ bits of information to Eve, for a certain prefixed parameter ${\epsilon }_{{\rm{I}}{\rm{R}}}$. If this last step is successful, it is guaranteed that Alice’s and Bob’s bit strings ${Z}_{{\rm{A}}}=A$ and ${Z}_{{\rm{B}}}$ satisfy $P({Z}_{{\rm{A}}}\ne {Z}_{{\rm{B}}})\le {\epsilon }_{{\rm{I}}{\rm{R}}}$. Otherwise, the protocol aborts.
5. *Parameter estimation*. Bob sets the parameter ${C}_{i}=\perp$ for the key generation rounds (*i.e*., when ${T}_{i}=0$) and ${C}_{i}={\omega }_{{\rm{C}}{\rm{H}}{\rm{S}}{\rm{H}}}({Z}_{{\text{B}}_{i}},{B}_{i},{X}_{i},{Y}_{i})$ for the test rounds (*i.e*., when ${T}_{i}=1$), with $i=1,2,\ldots ,{n}_{{\rm{S}}{\rm{H}}}$, and where ${Z}_{{\text{B}}_{i}}$ denotes the $i$-th bit of the string ${Z}_{{\rm{B}}}$ and the function ${\omega }_{{\rm{C}}{\rm{H}}{\rm{S}}{\rm{H}}}$ is defined as
  2 $${\omega }_{{\rm{C}}{\rm{H}}{\rm{S}}{\rm{H}}}(a,b,x,y)=\{\begin{array}{cc}1 & \mathrm{if}\,a\oplus b=x\cdot y,\\ 0 & \mathrm{otherwise},\end{array}$$
  with $\oplus$ denoting bit addition modulo $2$ and $\cdot$ denoting bit multiplication. The overall number of test rounds in which ${C}_{i}=1$ (and thus the parties win the CHSH game) is denoted by ${C}_{{\rm{S}}{\rm{H}}}={\sum }_{\{i:{T}_{i}=1\}}{C}_{i}$. This quantity allows to compute a lower bound on the number of secret bits that can be extracted from ${Z}_{{\rm{A}}}$ and ${Z}_{{\rm{B}}}$ using privacy amplification^28^. Bob aborts the protocol if the fraction of wins lies below a certain prefixed threshold value, *i.e*., when ${C}_{{\rm{S}}{\rm{H}}}/{n}_{{\rm{S}}{\rm{H}}} < \omega {|}_{{\rm{S}}{\rm{H}}}\gamma -{\delta }_{{\rm{e}}{\rm{s}}{\rm{t}}}$, where $\omega {|}_{{\rm{S}}{\rm{H}}}\in (3/4,(2+\sqrt{2})/4)$ is the expected winning rate of the CHSH game in the test rounds (which requires an experimental characterization of the setup), $\gamma$ is again the probability that Bob uses a successfully heralded round as a test round, and ${\delta }_{{\rm{e}}{\rm{s}}{\rm{t}}}$ is the confidence interval that defines the abortion threshold. That is, ${\delta }_{{\rm{e}}{\rm{s}}{\rm{t}}}$ is the maximum difference between the expected and the actual winning rates of the CHSH game that Bob accepts without aborting.
6. *Privacy amplification*. Alice and Bob apply a privacy amplification protocol to their bit strings ${Z}_{{\rm{A}}}$ and ${Z}_{{\rm{B}}}$ to obtain the final keys, ${K}_{{\rm{A}}}$ and ${K}_{{\rm{B}}}$, of length $l$. This protocol uses a randomness extractor that succeeds except with error probability ${\epsilon }_{{\rm{P}}{\rm{A}}}$.

The main protocol arguments are summarised in Table 1.

**Table 1:** List containing the main protocol arguments.

	Protocol arguments
${n}_{{\rm{S}}{\rm{H}}}$	Post-processing block size
$\gamma$	Probability of a test round
${\delta }_{{\rm{e}}{\rm{s}}{\rm{t}}}$	Confidence interval for the CHSH game winning rate
${\epsilon }_{{\rm{I}}{\rm{R}}}$	Error probability of information reconciliation
${\epsilon }_{{\rm{P}}{\rm{A}}}$	Error probability of privacy amplification
$l$	Length of the final keys ${K}_{{\rm{A}}}$ and ${K}_{{\rm{B}}}$

We remark that in the distribution step of the protocol, Alice and Bob need to store their signals until they choose their measurement settings and measure the signals in the third step of the protocol. For simplicity, below we will optimistically assume that, for this purpose, both of them hold noiseless and lossless quantum memories in their labs. Alternatively, they could also decide which rounds are key generation rounds and which ones are test rounds *a posteriori* by using the typical sifting step in QKD, though this approach results in a slightly less efficient solution. This is so because the data associated to Alice measuring ${\sigma }_{{\rm{x}}}$ and Bob measuring ${\sigma }_{{\rm{z}}}$ is not used in the protocol.

Also, we note that in a photonic implementation of the DIQKD scheme, the measurements ${X}_{i}$ and ${Y}_{i}$ can be realised by means of a polarization modulator that rotates the polarization state of the incoming signals, together with a PBS that separates vertical and horizontal polarization modes, followed by two photodetectors. For example, the rotation angles of the polarization modulator associated to the measurements ${\sigma }_{{\rm{z}}}$, ${\sigma }_{{\rm{x}}}$, ${\sigma }_{+}$ and ${\sigma }_{-}$ are $0$, $\pi /4$, $\pi /8$ and $-\pi /8$ radians, respectively. The observation of one single-photon in, say, horizontal (vertical) polarization is recorded by Alice as ${A}_{i}=0$ (${A}_{i}=1$), and the same applies to Bob.

### Evaluation

A major goal of this work is to determine the resources needed to implement DIQKD over long distances, with a particular emphasis on the detection efficiency and the quality of the entanglement sources. In this section, we use the device models and the secret key rate formula presented in the Methods section to evaluate the performance of the DIQKD protocol described above. All the relevant calculations required to reproduce the results presented here are included in the Supplementary Information.

To start up with, let us introduce some concepts and notation that shall be used in what follows. First of all, we will refer to two different DIQKD setups: the entanglement swapping relay (ESR) based setup and the polarizing qubit amplifier (PQA) based setup. The only difference between them lies in the qubit amplifier^34–36^, particularly, in the internal mechanism it uses to generate entangled photons for teleportation. To be precise, the ESR utilizes a source of entangled pairs directly^36^ (for instance, a PDC source), while the PQA^34,35^ generates entanglement by interfering single photon pulses in a linear optics network (see the Methods section). In both cases, we suppose that the teleportation is performed using a standard linear optics Bell state measurement (BSM). Regarding the photodetectors, we assume that all of them are photon-number-resolving (PNR) with the same detection efficiency, ${\eta }_{{\rm{d}}}$, and the same dark count probability, ${p}_{{\rm{d}}}$. Similarly, all the optical couplers used to link the light sources to the optical fiber are assumed to have the same coupling efficiency, ${\eta }_{{\rm{c}}}$. See the Methods section for further details about the device models, as well as for a detailed description of the qubit amplifiers we consider.

**Figure 2:**
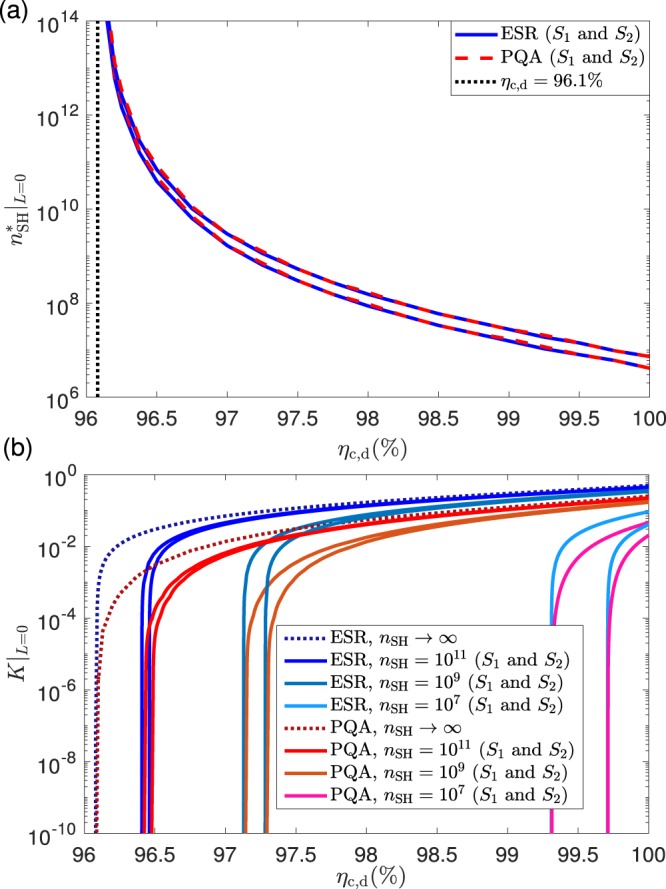
Performance evaluation of DIQKD with ideal photon sources at $L=0$ km, for the setup given in Fig. 1. Bluish (reddish) lines are used for the ESR (PQA) architecture. (**a**) Minimum value of the detection and coupling efficiency, ${\eta }_{{\rm{c}},{\rm{d}}}$, and minimum value of the block size, ${n}_{{\rm{S}}{\rm{H}}}$, required to obtain a zero-distance secret key rate $K{| }_{L=0}\ge 1{0}^{-10}$. Both sets of security requirements, ${S}_{1}$ and ${S}_{2}$, are compared for each qubit amplifier. Any combination of parameters ${\eta }_{{\rm{c}},{\rm{d}}}$ and ${n}_{{\rm{S}}{\rm{H}}}$ must be above the lower (upper) lines to achieve a secret key rate above the threshold value with the security requirements given by the sets ${S}_{1}$ (${S}_{2}$). The dotted black vertical line indicates the (asymptotic) minimum efficiency, ${\eta }_{{\rm{c}},{\rm{d}}}\approx 96.1{\rm{ \% }}$, which is the smallest detection efficiency that delivers a zero-distance asymptotic secret key rate ${K}_{\infty }{| }_{L=0}\ge 1{0}^{-10}$. (**b**) Zero-distance secret key rate, $K{| }_{L=0}$, as a function of ${\eta }_{{\rm{c}},{\rm{d}}}$ for various values of the block size ${n}_{{\rm{S}}{\rm{H}}}$. For each qubit amplifier, four different block sizes are considered: ${n}_{{\rm{S}}{\rm{H}}}\to {\rm{\infty }}$, ${n}_{{\rm{S}}{\rm{H}}}=1{0}^{11}$, ${n}_{{\rm{S}}{\rm{H}}}=1{0}^{9}$ and ${n}_{{\rm{S}}{\rm{H}}}=1{0}^{7}$. The finite secret key rates appear in pairs of solid lines, one for the security set ${S}_{1}$ (upper line) and another one for the security set ${S}_{2}$ (lower line). The asymptotic secret key rates corresponding to ${n}_{{\rm{S}}{\rm{H}}}\to {\rm{\infty }}$ are illustrated with dotted lines.

For simplicity, in the simulations below we assume that the coupling efficiency of the light sources is equal to the detection efficiency of the photodetectors, *i.e*., we set ${\eta }_{{\rm{c}}}={\eta }_{{\rm{d}}}={\eta }_{{\rm{c}},{\rm{d}}}$. This decision is motivated because DIQKD requires very high values of ${\eta }_{{\rm{c}},{\rm{d}}}$, so the effect of this simplification is negligible, and it reduces the number of experimental parameters to consider. Also, unless otherwise stated, we fix the dark count rate of the photodetectors to ${p}_{{\rm{d}}}=1{0}^{-7}$. Although this is a quite low value, it is achievable with current technology, for instance, by using superconducting nanowire single-photon detectors^39,40^ or even avalanche photodiodes^41^.

Regarding the security of the protocol, Alice and Bob should agree on the value of the secrecy parameter, ${\epsilon }_{{\rm{s}}{\rm{e}}{\rm{c}}}$, the correctness parameter, ${\epsilon }_{{\rm{c}}{\rm{o}}{\rm{r}}}$, and the robustness parameter, ${\epsilon }_{{\rm{r}}{\rm{o}}{\rm{b}}}$ in advance. Of course, these and other parameters, together with the secret key length of the protocol, are properly presented in the Methods section. For illustration purposes, in the simulations we consider two examples of security parameter sets $({\epsilon }_{{\rm{s}}{\rm{e}}{\rm{c}}},{\epsilon }_{{\rm{c}}{\rm{o}}{\rm{r}}},{\epsilon }_{{\rm{r}}{\rm{o}}{\rm{b}}})$, which we denote by ${S}_{1}$ and ${S}_{2}$. These sets are given in Table 2. In addition, we further simplify the numerics by fixing the value of the failure probability of the entropy accumulation theorem, ${\epsilon }_{{\rm{E}}{\rm{A}}}$, which is another parameter entering the security analysis. Precisely, we take ${\epsilon }_{{\rm{E}}{\rm{A}}}=1{0}^{-6}$ (${\epsilon }_{{\rm{E}}{\rm{A}}}=1{0}^{-10}$) for the set ${S}_{1}$ (${S}_{2}$). We remark, however, that according to our simulations the loss of generality that results from fixing the value of ${\epsilon }_{{\rm{E}}{\rm{A}}}$ in advance is very small. The secret key rate is then maximised over the remaining parameters. These include some security error terms affecting the secret key length, together with some experimental parameters that depend on the photon sources and on the qubit amplifier under consideration.

**Table 2:** Sets of security parameters ${\epsilon }_{{\rm{s}}{\rm{e}}{\rm{c}}}$, ${\epsilon }_{{\rm{c}}{\rm{o}}{\rm{r}}}$ and ${\epsilon }_{{\rm{r}}{\rm{o}}{\rm{b}}}$ considered in the performance evaluation of DIQKD. The set ${S}_{1}$ provides a lower level of security than the set ${S}_{2}$.

	${{\boldsymbol{\epsilon }}}_{{\bf{s}}{\bf{e}}{\bf{c}}}$	${{\boldsymbol{\epsilon }}}_{{\bf{c}}{\bf{o}}{\bf{r}}}$	${{\boldsymbol{\epsilon }}}_{{\bf{r}}{\bf{o}}{\bf{b}}}$
${S}_{1}$	$1{0}^{-5}$	$1{0}^{-10}$	$1{0}^{-2}$
${S}_{2}$	$1{0}^{-9}$	$1{0}^{-15}$	$1{0}^{-3}$

Importantly, the number of signals transmitted in an execution of the protocol, which we shall denote by $N$, is not determined a priori. Indeed, as described in the previous section, it is the target number of successful heralding events that we fix, ${n}_{{\rm{S}}{\rm{H}}}$. Therefore, in the absence of real experimental data, we set $N$ to its expected value $\left\langle N\right\rangle$ for the simulations. In this way, what we shall refer to as the secret key rate in this section is the ratio

3 $$K=\frac{l}{\left\langle N\right\rangle },$$

$l$ being the secret key length of the protocol. Similarly, the *conditional* secret key rate (*i.e*., the number of secret key bits per successful heralding event) reads $K{|}_{{\rm{S}}{\rm{H}}}=l/{n}_{{\rm{S}}{\rm{H}}}$, and it is related to $k$ via $K={P}_{{\rm{S}}{\rm{H}}}K{|}_{{\rm{S}}{\rm{H}}}$, where ${P}_{{\rm{S}}{\rm{H}}}$ is the successful heralding probability of the qubit amplifier. Note that we are assuming here that ${P}_{{\rm{S}}{\rm{H}}}$ is the same for all the rounds of the protocol, in such a way that

4 $$\langle N\rangle =\frac{{n}_{{\rm{S}}{\rm{H}}}}{{P}_{{\rm{S}}{\rm{H}}}}$$

Finally, in all the plots below we assume a threshold value for the secret key rate $K$ as low as $1{0}^{-10}$. That is, whenever the resulting secret key rate is smaller than this threshold value, it is considered to be impractical and we neglect it. Although this choice is arbitrary, $1{0}^{-10}$ seems to be a reasonable value: even with an ideal entanglement source with a high repetition-rate of 10 GHz, one could only extract 1 secret bit/s at most, which is probably too low for most applications.

#### Ideal sources

We start by analyzing the ideal scenario where Alice and Bob hold perfect photon sources. Obviously, this case provides the best possible performance, and thus it can be used as a reference about the minimum resources (say, *e.g*., the minimum value of the detection and coupling efficiency, ${\eta }_{{\rm{c}},{\rm{d}}}$, and the minimum block size, ${n}_{{\rm{S}}{\rm{H}}}$) that are required to achieve a certain secret key rate.

More precisely, we consider here that the entanglement source ${\rho }_{ab}$ at Alice’s lab generates perfect polarization Bell pairs. On the other side, the entangled states ${\rho }_{bc}$ used for teleportation are different for each qubit amplifier architecture. In the case of a PQA, ${\rho }_{bc}$ is generated via the interference of two single photon signals, ${\rho }_{{\rm{s}}{\rm{i}}{\rm{n}}{\rm{g}}{\rm{l}}{\rm{e}}}^{h}$ and ${\rho }_{{\rm{s}}{\rm{i}}{\rm{n}}{\rm{g}}{\rm{l}}{\rm{e}}}^{v}$, on a beamsplitter of tunable transmittance $t$ (see the Methods section for more details). Here, we set ${\rho }_{{\rm{s}}{\rm{i}}{\rm{n}}{\rm{g}}{\rm{l}}{\rm{e}}}^{h}$ (${\rho }_{{\rm{s}}{\rm{i}}{\rm{n}}{\rm{g}}{\rm{l}}{\rm{e}}}^{v}$) to be a perfect single-photon source generating horizontally (vertically) polarised single photons. Similarly, in the case of an ESR, we directly set ${\rho }_{bc}$ to be a perfect source of polarization Bell pairs, as ${\rho }_{ab}$.

#### No channel loss

To begin with, we compare the achievable performance when using PQAs and ESRs in the absence of channel loss, *i.e*., we set the transmission distance $L$ to zero. This scenario allows us to determine the minimum value of ${n}_{{\rm{S}}{\rm{H}}}$ as a function of ${\eta }_{{\rm{c}},{\rm{d}}}$.

As we will show below, it turns out that ${\eta }_{{\rm{c}},{\rm{d}}}$ is quite high even in this ideal scenario. This means that the probability that any of these two amplifiers provides a spurious success at Bob’s side due to the dark counts of the PNR detectors within the BSM is negligible compared to that of a genuine success triggered by a single photon from Alice. Therefore, for simplicity, in this subsection we set the dark count rate ${p}_{{\rm{d}}}$ equal to zero. With this approximation and on the basis of the ideal form of ${\rho }_{ab}$ and ${\rho }_{bc}$ just presented, one can readily use the models for the detectors and the optical couplers to derive analytical expressions for the three experimental parameters that enter the secret key rate formula: the successful heralding probability of the qubit amplifier, ${P}_{{\rm{S}}{\rm{H}}}$ (entering $K$ via $\left\langle N\right\rangle$), the expected conditional winning rate at the CHSH game, $\omega {|}_{{\rm{S}}{\rm{H}}}$, and the expected conditional quantum bit error rate, $Q{|}_{{\rm{S}}{\rm{H}}}$ (the latter two entering $K$ via $l$). In the ESR-based setup, one obtains

5 $$\begin{array}{ccc}{P}_{{\rm{S}}{\rm{H}}}^{{\rm{E}}{\rm{S}}{\rm{R}}} & = & \frac{{\xi }^{2}}{2},\\ \omega {|}_{{\rm{S}}{\rm{H}}}^{{\rm{E}}{\rm{S}}{\rm{R}}} & = & \frac{2+\sqrt{2}}{4}{\xi }^{2}+\frac{3}{4}{(1-\xi )}^{2}+\xi (1-\xi ),\\ Q{|}_{{\rm{S}}{\rm{H}}}^{{\rm{E}}{\rm{S}}{\rm{R}}} & = & \xi (1-\xi ),\end{array}$$

where the parameter $\xi ={\eta }_{{\rm{c}},{\rm{d}}}^{2}$. We remark that the successful heralding probability scales with ${\xi }^{2}$, *i.e*., with ${\eta }_{{\rm{c}},{\rm{d}}}^{4}$, because it requires the successful detection of two photons in the BSM. Note that the detection probability of each photon scales with $\xi$, as this quantity includes both the detection and the coupling factors, *i.e*., $\xi ={\eta }_{c}{\eta }_{d}={\eta }_{c,d}^{2}$. Similarly, in the PQA-based setup, one finds

6 $$\begin{array}{ccc}{P}_{{\rm{S}}{\rm{H}}}^{{\rm{P}}{\rm{Q}}{\rm{A}}} & = & (1-t){\xi }^{2}[1-\xi (1-t)],\\ \omega {|}_{{\rm{S}}{\rm{H}}}^{{\rm{P}}{\rm{Q}}{\rm{A}}} & = & \frac{1}{1-\xi (1-t)}[\frac{2+\sqrt{2}}{4}t{\xi }^{2}+\frac{3}{4}{(1-\xi )}^{2}\\  &  & +\frac{(1+t)}{2}\xi (1-\xi )],\\ Q{|}_{{\rm{S}}{\rm{H}}}^{{\rm{P}}{\rm{Q}}{\rm{A}}} & = & \frac{(1+t)\xi (1-\xi )}{2[1-\xi (1-t)]},\end{array}$$

where the parameter $t$ corresponds to the transmittance of the BS within the amplifier. The reader is referred to the Supplementary Information for the detailed calculation of ${P}_{{\rm{S}}{\rm{H}}}$, $\omega {|}_{{\rm{S}}{\rm{H}}}$ and $Q{|}_{{\rm{S}}{\rm{H}}}$ in more general settings that reduce to Eqs. (5) and (6) when ideal sources are considered.

From Eq. (6), it is evident that there is a trade-off on the coefficient $t$. The terms $\omega {|}_{{\rm{S}}{\rm{H}}}^{{\rm{P}}{\rm{Q}}{\rm{A}}}$ and $Q{|}_{{\rm{S}}{\rm{H}}}^{{\rm{P}}{\rm{Q}}{\rm{A}}}$ favor $t\approx 1$, and thus the conditional secret key rate $K{|}_{{\rm{S}}{\rm{H}}}$, which depends on these parameters but not on ${P}_{{\rm{S}}{\rm{H}}}^{{\rm{P}}{\rm{Q}}{\rm{A}}}$, also favors $t\approx 1$. Indeed, in the limit $t\to 1$ we have that $\omega {|}_{{\rm{S}}{\rm{H}}}^{{\rm{P}}{\rm{Q}}{\rm{A}}}=\omega {|}_{{\rm{S}}{\rm{H}}}^{{\rm{E}}{\rm{S}}{\rm{R}}}$ and $Q{|}_{{\rm{S}}{\rm{H}}}^{{\rm{P}}{\rm{Q}}{\rm{A}}}=Q{|}_{{\rm{S}}{\rm{H}}}^{{\rm{E}}{\rm{S}}{\rm{R}}}$. On the other hand, ${P}_{{\rm{S}}{\rm{H}}}^{{\rm{P}}{\rm{Q}}{\rm{A}}}$ is maximised when $t=1-{(2\xi )}^{-1}$, and it actually vanishes when $t=1$. This behaviour can be easily understood by examining the states ${\rho }_{bc}={\left|\phi \right\rangle }_{bc}\left\langle \phi \right|$ generated with the single-photon interference inside the PQA, whose expression with ideal single photon sources is of the form ${\left|\phi \right\rangle }_{bc}=(1-t){\left|\psi \right\rangle }_{bb}+t{\left|\varphi \right\rangle }_{cc}-\sqrt{2t(1-t)}{\left|\chi \right\rangle }_{bc}$ and the states ${\left|\psi \right\rangle }_{bb}$, ${\left|\varphi \right\rangle }_{cc}$ and ${\left|\chi \right\rangle }_{bc}$ are given in Eq. (17) of the Methods section. In short, by setting $t$ close to 1 we have that whenever a successful heralding takes place at the qubit amplifier, this event is due to the entangled pair ${\left|\chi \right\rangle }_{bc}$ with a high probability, and thus $K{|}_{{\rm{S}}{\rm{H}}}$ is maximised. On the contrary, the lower the transmittance $t$ is, the more likely it is that a success comes from the spurious term ${\left|\psi \right\rangle }_{bb}$ and, consequently, $K{|}_{{\rm{S}}{\rm{H}}}$ is minimised. In our simulations, we numerically optimise the value of $t$ so that the overall secret key rate $K$ is maximised.

Also, we remark that by substituting the parameters from Eqs. (5) and (6) in Eq. (31), equating the resulting expression to zero and numerically solving for $\xi$, one obtains the minimum value of $\xi$ required for a positive key rate. This minimum value happens to be very large, $\xi \,\gtrapprox \,92.3 \%$ (or, equivalently, ${\eta }_{{\rm{c}},{\rm{d}}}=\sqrt{\xi }\,\gtrapprox \,96.1{\rm{ \% }}$) for both types of qubit amplifiers. Similarly, from Eqs. (5) and (6), it can be shown that, irrespectively of the value of $t$, whenever $\xi \ge 50 \%$ we have that ${P}_{{\rm{S}}{\rm{H}}}^{{\rm{E}}{\rm{S}}{\rm{R}}} > {P}_{{\rm{S}}{\rm{H}}}^{{\rm{P}}{\rm{Q}}{\rm{A}}}$, $\omega {|}_{{\rm{S}}{\rm{H}}}^{{\rm{E}}{\rm{S}}{\rm{R}}}\ge \omega {|}_{{\rm{S}}{\rm{H}}}^{{\rm{P}}{\rm{Q}}{\rm{A}}}$ and $Q{|}_{{\rm{S}}{\rm{H}}}^{{\rm{E}}{\rm{S}}{\rm{R}}}\le Q{|}_{{\rm{S}}{\rm{H}}}^{{\rm{P}}{\rm{Q}}{\rm{A}}}$. That is, an ESR-based qubit amplifier always outperforms a PQA in the absence of channel loss, if perfect sources are assumed. In Fig. 2(a) we plot the minimum block size, ${n}_{{\rm{S}}{\rm{H}}}$, and the minimum detection efficiency, ${\eta }_{{\rm{c}},{\rm{d}}}$, that are needed to obtain a secret key rate above the threshold value of $1{0}^{-10}$ at $L=0$ km. We denote this secret key rate by $K{| }_{L=0}$ and the minimum block size by ${n}_{{\rm{S}}{\rm{H}}}^{\ast }{|}_{L=0}$. The value of ${n}_{{\rm{S}}{\rm{H}}}^{\ast }{|}_{L=0}$ is obtained for each ${\eta }_{{\rm{c}},{\rm{d}}}$ via exhaustive numerical search over all the free parameters contained in the finite-key rate formula. The solid (dashed) bluish (reddish) lines correspond to the ESR (PQA) architecture, and in each case the lower (upper) line uses the set of security requirements ${S}_{1}$ (${S}_{2}$) of Table 2. Remarkably, despite the security parameters of ${S}_{2}$ being significantly more demanding than those of ${S}_{1}$, it turns out that the set ${S}_{2}$ does not require much larger block sizes than ${S}_{1}$.

**Figure 3:**
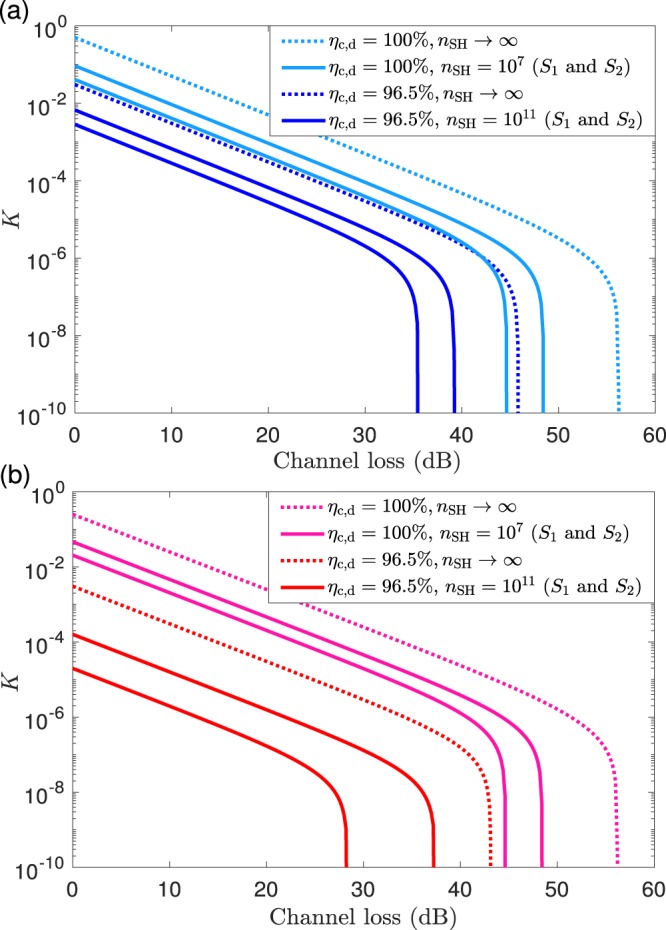
Secret key rate $K$ as a function of the overall channel loss $\Lambda$ measured in dB for the case of ideal photon sources. The considered setup is again that of Fig. 1. (**a**) Corresponds to the ESR architecture and (**b**) to the PQA architecture. In both figures, we use two different detection and coupling efficiencies, ${\eta }_{{\rm{c}},{\rm{d}}}=100{\rm{ \% }}$ and ${\eta }_{{\rm{c}},{\rm{d}}}=96.5{\rm{ \% }}$, each of them tagged with a different color. For each value of the efficiency, we plot the asymptotic secret key rate ${K}_{\infty }$ (dotted line), together with two finite-key rates for different values of ${n}_{{\rm{S}}{\rm{H}}}$ (solid lines). Each finite-key rate is plotted twice, the upper (lower) line corresponding to the security settings ${S}_{1}$ (${S}_{2}$), and in both cases a common block size ${n}_{{\rm{S}}{\rm{H}}}$ close to the critical one is assumed (see Fig. 2(a)). More precisely, we take ${n}_{{\rm{S}}{\rm{H}}}=1{0}^{7}$ when ${\eta }_{{\rm{c}},{\rm{d}}}=100{\rm{ \% }}$ and ${n}_{{\rm{S}}{\rm{H}}}=1{0}^{11}$ when ${\eta }_{{\rm{c}},{\rm{d}}}=96.5{\rm{ \% }}$. By increasing the value of ${\eta }_{{\rm{c}},{\rm{d}}}$ and/or ${n}_{{\rm{S}}{\rm{H}}}$ the finite-key rates approach those of the optimal scenario, which corresponds to ${K}_{\infty }$ assuming ${\eta }_{{\rm{c}},{\rm{d}}}=100{\rm{ \% }}$.

Also, Fig. 2(a) indicates that both qubit amplifiers require a similar minimum block size, ${n}_{{\rm{S}}{\rm{H}}}^{\ast }{|}_{L=0}$, to deliver a secret key rate above the threshold value. Indeed, it is easy to show that if the threshold value for the secret key rate were zero (instead of $1{0}^{-10}$) then the value of ${n}_{{\rm{S}}{\rm{H}}}^{\ast }{|}_{L=0}$ would be equal for both qubit amplifiers. However, the fact that we use a threshold value greater than zero implies that ${n}_{{\rm{S}}{\rm{H}}}^{\ast }{|}_{L=0}$ is always slightly lower for the ESR than for the PQA. This is so because, even though the latter can mimic the conditional secret key rate of the former for any efficiency ${\eta }_{{\rm{c}},{\rm{d}}}$, the ESR has a higher success probability ${P}_{{\rm{S}}{\rm{H}}}^{{\rm{E}}{\rm{S}}{\rm{R}}}$, thus leading to a higher overall secret key rate. Nevertheless, this effect cannot be fully appreciated with the resolution of Fig. 2(a).

Finally, the dotted black vertical line illustrated in Fig. 2(a) corresponds to the (asymptotic) minimum efficiency, ${\eta }_{{\rm{c}},{\rm{d}}}\approx 96.1{\rm{ \% }}$, required to obtain ${K}_{\infty }{| }_{L=0}\ge 1{0}^{-10}$. That is, no secret key rate above such threshold value is possible when ${\eta }_{{\rm{c}},{\rm{d}}}\,\lessapprox \,96.1{\rm{ \% }}$, no matter how much we increase the block size.

In what follows, we will refer to the lines in Fig. 2(a) as the *critical lines*, since every pair $({\eta }_{{\rm{c}},{\rm{d}}},{n}_{{\rm{S}}{\rm{H}}})$ lying below these lines delivers a negligible secret key rate with the corresponding security requirements.

Figure 2(b) shows the zero-distance secret key rate, $K{| }_{L=0}$, as a function of the detection and coupling efficiency, ${\eta }_{{\rm{c}},{\rm{d}}}$, for different values of the block size, ${n}_{{\rm{S}}{\rm{H}}}$. As already discussed above, the ESR architecture always leads to larger secret key rates for all values of ${\eta }_{{\rm{c}},{\rm{d}}}$, while the minimum efficiencies required to have a secret key rate larger than the threshold value are roughly equal for both qubit amplifiers. Again, the small mismatch between the minimum efficiencies required by both amplifiers occurs because the selected threshold is greater than zero. Otherwise, the minimum efficiencies would match. For illustrative purposes, Fig. 2(b) considers four different block sizes: ${n}_{{\rm{S}}{\rm{H}}}\to {\rm{\infty }}$, ${n}_{{\rm{S}}{\rm{H}}}={10}^{11}$, ${n}_{{\rm{S}}{\rm{H}}}={10}^{9}$ and ${n}_{{\rm{S}}{\rm{H}}}={10}^{17}$. As already shown in Fig. 2(a), the smaller the block size is, the larger the value of the minimum efficiency ${\eta }_{{\rm{c}},{\rm{d}}}$ that is required. For instance, for a block size as large as, say, ${n}_{{\rm{S}}{\rm{H}}}={10}^{11}$, and if one considers the weaker set of security requirements ${S}_{1}$, the minimum efficiency is at least ${\eta }_{{\rm{c}},{\rm{d}}}\approx 96.4{\rm{ \% }}$. Also, for any given value of ${n}_{{\rm{S}}{\rm{H}}}$, the greater the detection efficiency considered (with respect to its minimum value), the closer the resulting secret key rates corresponding to the security settings ${S}_{1}$ and ${S}_{2}$ become. This is so because, in this situation, the effect of finite statistics is less prominent. Note that in the limit given by the asymptotic regime, the secret key rate ${K}_{\infty }$ does not depend on the security sets ${S}_{1}$ and ${S}_{2}$, but these sets are only relevant in the finite-key regime.

#### Channel loss

In this subsection, we consider the effect of channel loss as modeled in the Methods section, where it is parametrised by the quantity $\Lambda =\alpha L$ measured in dB. Here and throughout this work, the transmission distance $L$ is the distance between Alice’s lab and Bob’s lab. For example, when only Bob has a qubit amplifier, then $L$ represents the distance between Alice’s entanglement source ${\rho }_{ab}$ (located inside Alice’s lab) and Bob’s qubit amplifier (located inside Bob’s lab). Similarly, when both Alice and Bob have a qubit amplifier (see the Supplementary Information), $L$ is the distance between Alice’s qubit amplifier and Bob’s qubit amplifier. Also, we set here the dark count rate of the detectors to ${p}_{{\rm{d}}}=1{0}^{-7}$, as the effect of dark counts becomes relevant in this scenario.

Figure 3 plots the secret key rate $K$ as a function of $\Lambda$ for various values of ${\eta }_{{\rm{c}},{\rm{d}}}$ and ${n}_{{\rm{S}}{\rm{H}}}$, and for the two qubit amplifier architectures under consideration. More precisely, we use two values for ${\eta }_{{\rm{c}},{\rm{d}}}$: the ideal one, *i.e*., ${\eta }_{{\rm{c}},{\rm{d}}}=100{\rm{ \% }}$, and another one close to the threshold value of $96.1 \%$ discussed above, say, ${\eta }_{{\rm{c}},{\rm{d}}}=96.5{\rm{ \% }}$. Moreover, for each of these values of the efficiency ${\eta }_{{\rm{c}},{\rm{d}}}$, we plot three different secret key rates: the asymptotic one ${K}_{\infty }$, and two finite-key rates, one for the security settings ${S}_{1}$ and another one for the security settings ${S}_{2}$. In both finite-key cases, we use a common block size ${n}_{{\rm{S}}{\rm{H}}}$ close to the critical value obtained from Fig. 2(a). Specifically, we set ${n}_{{\rm{S}}{\rm{H}}}=1{0}^{7}$ when ${\eta }_{{\rm{c}},{\rm{d}}}=100{\rm{ \% }}$, and ${n}_{{\rm{S}}{\rm{H}}}=1{0}^{11}$ when ${\eta }_{{\rm{c}},{\rm{d}}}=96.5{\rm{ \% }}$. In doing so, and for the considered security analysis, we are simultaneously providing upper bounds (given by ${K}_{\infty }$) and lower bounds (given by the finite-key rates) to the finite-key performance that could be achieved with the chosen detection and coupling efficiencies, and the security requirements. By increasing the value of ${n}_{{\rm{S}}{\rm{H}}}$, the finite-key rates approach the asymptotic scenario. Also, ${K}_{\infty }$ with ${\eta }_{{\rm{c}},{\rm{d}}}=100{\rm{ \% }}$ provides a clear upper bound for the achievable secret key rate with the security analysis introduced in Sec. IV B.

**Figure 4:**
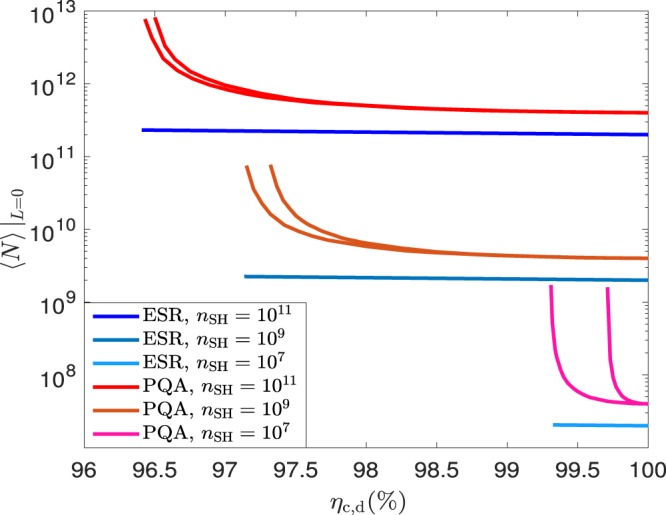
Average number of signals, $\left\langle N\right\rangle {| }_{L=0}$, that Alice needs to send Bob to collect a data block size equal to ${n}_{{\rm{S}}{\rm{H}}}$ when using ideal photon sources, as a function of the detection and coupling efficiency ${\eta }_{{\rm{c}},{\rm{d}}}$ at $L=0$ km. As in Eqs. 5 and 6), in this figure we disregard dark counts because their effect at $L=0$ km is negligible. Also, we set the free experimental and security parameters to those values that optimise the secret key rate given by Fig. 2(b). The figure considers three different data block sizes, *i.e*., ${n}_{{\rm{S}}{\rm{H}}}=1{0}^{7}$, ${n}_{{\rm{S}}{\rm{H}}}=1{0}^{9}$ and ${n}_{{\rm{S}}{\rm{H}}}=1{0}^{11}$. All the plots are cut at the value of ${\eta }_{{\rm{c}},{\rm{d}}}$ for which the resulting secret key rate is below the threshold value of $1{0}^{-10}$. We note that, since in the case of the ESR the value of $\left\langle N\right\rangle {| }_{L=0}$ does not depend on any parameter to be optimised, the cases ${S}_{1}$ and ${S}_{2}$ only differ in the minimum ${\eta }_{{\rm{c}},{\rm{d}}}$ that still provides $K\ge 1{0}^{-10}$, which can be extracted from Fig. 2. In the case of the PQA, $\left\langle N\right\rangle {| }_{L=0}$ depends on the transmittance $t$ to be optimised, and therefore the cases ${S}_{1}$ and ${S}_{2}$ differ more from each other.

Figures 3(a) and 3(b) further show, as expected, that in the case of ideal sources the ESR architecture outperforms the PQA architecture also in the presence of channel loss.

As a final remark, we note that if Bob did not use a qubit amplifier, then the maximum possible value of $\Lambda$ would be very limited. Indeed, it can be shown that in the case of ideal sources, and even if one sets ${\eta }_{{\rm{c}},{\rm{d}}}=100{\rm{ \% }}$ and ${n}_{{\rm{S}}{\rm{H}}}\to {\rm{\infty }}$, the maximum value of $\Lambda$ is as low as $\Lambda \,\lessapprox \,0.7$ dB. See the Supplementary Information for further details.

#### Time constraints

In the discussion so far, we have not considered the duration of a DIQKD session, which is another crucial experimental parameter. Indeed, this parameter imposes strong restrictions on the loss that DIQKD can tolerate. We study it in this section.

According to the protocol described earlier, the post-processing block size, ${n}_{{\rm{S}}{\rm{H}}}$, is fixed a priori. This means, in particular, that the number of transmitted signals, $N$, and thus the duration of the distribution step of the protocol, which we shall denote by $\tau$, are random variables. Their mean values are given by Eq. (4) and $\left\langle \tau \right\rangle =\nu \left\langle N\right\rangle$, respectively, where $\nu$ represents the clock rate of system. From Eq. (4) we have that, for a given ${n}_{{\rm{S}}{\rm{H}}}$, the value of $\left\langle N\right\rangle$ increases when the success probability of the qubit amplifier decreases, for instance, due to channel and/or detection loss. Indeed, according to Eqs. 5 and 6) we find that $\left\langle N\right\rangle$ at $L=0$ km, which we will denote by $\left\langle N\right\rangle {| }_{L=0}$, is, in the case of an ESR, equal to

7 $$\langle N\rangle {|}_{L=0}=\frac{2{n}_{{\rm{S}}{\rm{H}}}}{{\eta }_{{\rm{c}},{\rm{d}}}^{2}},$$

while in the case of a PQA it satisfies

8 $$\begin{array}{ccc}\langle N\rangle {|}_{L=0} & = & \frac{{n}_{{\rm{S}}{\rm{H}}}}{(1-t){\eta }_{{\rm{c}},{\rm{d}}}^{4}[1-{\eta }_{{\rm{c}},{\rm{d}}}^{2}(1-t)]}\\  & \approx  & \frac{{n}_{{\rm{S}}{\rm{H}}}}{(1-t){\eta }_{{\rm{c}},{\rm{d}}}^{4}}.\end{array}$$

This is illustrated in Fig. 4, which shows $\left\langle N\right\rangle {| }_{L=0}$ as a function of ${\eta }_{{\rm{c}},{\rm{d}}}$ when ${n}_{{\rm{S}}{\rm{H}}}=\{1{0}^{7},1{0}^{9},1{0}^{11}\}$. From Fig. 4 we find that the value of $\left\langle N\right\rangle {| }_{L=0}$ associated to the PQA presents a much steeper slope than that of the ESR architecture when ${\eta }_{{\rm{c}},{\rm{d}}}$ decreases. This is because the optimal transmittance $t$ of the PQA approaches $1$ in that regime.

**Figure 5:**
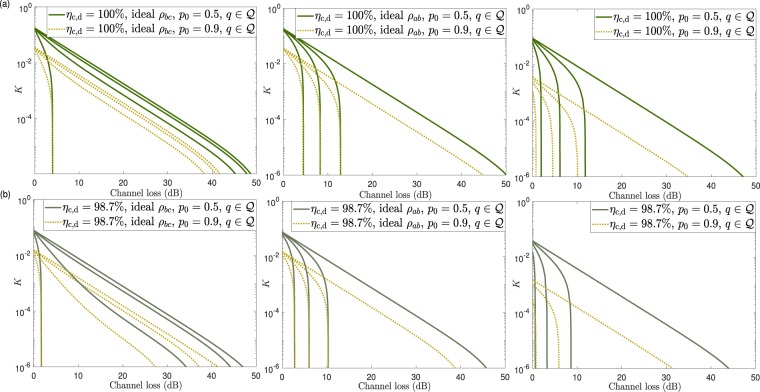
Secret key rate $K$ as a function of the overall channel loss $\Lambda$ measured in dB for generic photonic sources and assuming the ESR architecture. (**a**) considers a detection and coupling efficiency ${\eta }_{{\rm{c}},{\rm{d}}}=100{\rm{ \% }}$ and (**b**) considers ${\eta }_{{\rm{c}},{\rm{d}}}=98.7{\rm{ \% }}$. Each figure evaluates three different cases. The first (second) case, assumes that the entanglement source ${\rho }_{bc}$ (${\rho }_{ab}$) is an ideal entanglement source, while ${\rho }_{ab}$ (${\rho }_{bc}$) is characterised by the parameters ${p}_{0}$ and $q={p}_{2}/{p}_{1}$. The third case considers that both ${\rho }_{bc}$ and ${\rho }_{ab}$ are characterised by the parameters ${p}_{0}$ and $q={p}_{2}/{p}_{1}$. All figures consider two possible values for ${p}_{0}$, *i.e*., ${p}_{0}=0.5$ (solid lines) and ${p}_{0}=0.9$ (dotted lines), and four different values for the parameter $q\in {\mathscr{Q}}=\{0,1{0}^{-2},1{0}^{-1.5},1{0}^{-1}\}$. Also, for concreteness, in all cases we set ${n}_{{\rm{S}}{\rm{H}}}=1{0}^{9}$ and choose the security settings ${S}_{1}$.

In the scenario where $L > 0$ km, the success probability of the qubit amplifier decreases exponentially with the channel loss. In particular, we find that the value of $\left\langle N\right\rangle {| }_{L\ge 0}$ in this case is given by

9 $$\begin{array}{ccc}\langle N\rangle {|}_{L\ge 0} & = & \frac{2{n}_{{\rm{S}}{\rm{H}}}}{{\eta }_{{\rm{c}},{\rm{d}}}^{2}}\{(1-4{p}_{{\rm{d}}}){\eta }_{{\rm{c}}{\rm{h}}}{{\eta }_{{\rm{c}},{\rm{d}}}}^{2}\\  &  & {+4{p}_{{\rm{d}}}[1+{\eta }_{{\rm{c}}{\rm{h}}}(1-2{{\eta }_{{\rm{c}},{\rm{d}}}}^{2})]\}}^{-1},\end{array}$$

for the ESR architecture, and

10 $$\begin{array}{ccc}\langle N\rangle {|}_{L\ge 0} & = & \frac{{n}_{{\rm{S}}{\rm{H}}}}{{\eta }_{{\rm{c}},{\rm{d}}}^{2}[1-{\eta }_{{\rm{c}},{\rm{d}}}^{2}(1-t)]}\{(1-10{p}_{{\rm{d}}})(1-t)\\  &  & {\times {\eta }_{{\rm{c}}{\rm{h}}}{\eta }_{{\rm{c}},{\rm{d}}}^{2}+4{p}_{{\rm{d}}}(1-t+{\eta }_{{\rm{c}}{\rm{h}}}/2)\}}^{-1},\end{array}$$

for the PQA. In these two equations, for simplicity, the success probability ${P}_{{\rm{S}}{\rm{H}}}$ is computed to the first order in ${p}_{{\rm{d}}}$.

We recall that Fig. 3 shows the overall channel loss that Alice and Bob can tolerate before the secret key rate drops down to zero, and which secret key rates they can attain depending on the channel loss. This information is complemented by Table 3, which, for every finite block size curve in the figure, tells us the value of $\left\langle N\right\rangle {| }_{L\ge 0}$ required at the extreme channel loss where the key rate starts dropping down to zero. These values are easily translated into time constraints, and they are quite large from a practical point of view. Indeed, if one considers, for example, that the clock rate of the system is, say, $10$ GHz, we find that when ${\eta }_{{\rm{c}},{\rm{d}}}=96.5{\rm{ \% }}$ it would take about $2.1$ ($37.0$) days to establish a secret key of length $7.56\times 1{0}^{7}$ ($8.64\times 1{0}^{7}$) bits—out of a block size ${n}_{{\rm{S}}{\rm{H}}}=1{0}^{11}$—over a channel loss of 39 dB (37 dB) when using the ESR (PQA) architecture and the security settings given by ${S}_{1}$. Of course, the result improves when ${\eta }_{{\rm{c}},{\rm{d}}}$ increases. For instance, if ${\eta }_{{\rm{c}},{\rm{d}}}=100{\rm{ \% }}$ then it would take of the order of $120$ ($250$) seconds to establish a secret key of length $1.56\times 1{0}^{5}$ ($1.58\times 1{0}^{5}$)—out of a block size ${n}_{{\rm{S}}{\rm{H}}}=1{0}^{7}$—over 48 dB when using the ESR (PQA) architecture and the security settings given by ${S}_{1}$. These particular examples are given to illustrate the maximum duration of a DIQKD session, but the duration of a session with arbitrary values of ${n}_{{\rm{S}}{\rm{H}}}$, ${\eta }_{{\rm{c}},{\rm{d}}}$ and $L$ can be computed directly from Eqs. (9) and (10).

**Table 3:** Average number of signals, $\left\langle N\right\rangle$, that Alice needs to send Bob to collect a data block size equal to ${n}_{{\rm{S}}{\rm{H}}}$, when using ideal photon sources and setting the channel loss to the cutoff value for which the secret key rate starts dropping down to zero in Fig. 3. The dark count rate of the photodetectors is set to ${p}_{{\rm{d}}}=1{0}^{-7}$, and the detection and coupling efficiency is ${\eta }_{{\rm{c}},{\rm{d}}}$. The considered combinations of ${\eta }_{{\rm{c}},{\rm{d}}}$ and ${n}_{{\rm{S}}{\rm{H}}}$ correspond to the cases illustrated in Fig. 3, and both sets of security settings, ${S}_{1}$ and ${S}_{2}$, are considered.

${{\boldsymbol{S}}}_{{\bf{1}}}$	${{\boldsymbol{\eta }}}_{{\rm{c}},{\rm{d}}}$	${{\boldsymbol{n}}}_{{\rm{S}}{\rm{H}}}$	${{\boldsymbol{\Lambda }}}_{{\rm{c}}{\rm{u}}{\rm{t}}{\rm{o}}{\rm{f}}{\rm{f}}}$	${{\boldsymbol{K}}}_{{\rm{c}}{\rm{u}}{\rm{t}}{\rm{o}}{\rm{f}}{\rm{f}}}$	${\boldsymbol{\langle }}{\boldsymbol{N}}{\boldsymbol{\rangle }}$
ESR	$100 \%$	$1{0}^{7}$	$48$ dB	$1.3\times 1{0}^{-7}$	$1.2\times 1{0}^{12}$
ESR	$96.5 \%$	$1{0}^{11}$	$39$ dB	$4.2\times 1{0}^{-8}$	$1.8\times 1{0}^{15}$
PQA	$100 \%$	$1{0}^{7}$	$48$ dB	$6.3\times 1{0}^{-8}$	$2.5\times 1{0}^{12}$
PQA	$96.5 \%$	$1{0}^{11}$	$37$ dB	$2.7\times 1{0}^{-9}$	$3.2\times 1{0}^{16}$
${{\boldsymbol{S}}}_{{\bf{2}}}$	${{\boldsymbol{\eta }}}_{{\rm{c}},{\rm{d}}}$	${{\boldsymbol{n}}}_{{\rm{S}}{\rm{H}}}$	${{\boldsymbol{\Lambda }}}_{{\rm{c}}{\rm{u}}{\rm{t}}{\rm{o}}{\rm{f}}{\rm{f}}}$	${{\boldsymbol{K}}}_{{\rm{c}}{\rm{u}}{\rm{t}}{\rm{o}}{\rm{f}}{\rm{f}}}$	${\boldsymbol{\langle }}{\boldsymbol{N}}{\boldsymbol{\rangle }}$
ESR	$100 \%$	$1{0}^{7}$	$44$ dB	$2.1\times 1{0}^{-7}$	$5.0\times 1{0}^{11}$
ESR	$96.5 \%$	$1{0}^{11}$	$35$ dB	$9.8\times 1{0}^{-8}$	$7.2\times 1{0}^{14}$
PQA	$100 \%$	$1{0}^{7}$	$44$ dB	$1.0\times 1{0}^{-7}$	$9.9\times 1{0}^{11}$
PQA	$96.5 \%$	$1{0}^{11}$	$28$ dB	$2.4\times 1{0}^{-9}$	$4.0\times 1{0}^{15}$

#### Generic sources

In this section we investigate the effect that vacuum pulses and multiple photon pairs, generated by practical entanglement sources, have on the performance of DIQKD. For concreteness, and also motivated by the results of the previous subsection, we focus on the ESR architecture for the qubit amplifier at Bob’s lab, and we consider entanglement sources ${\rho }_{ab}$ and ${\rho }_{bc}$ (as in Fig. 1) that generate a coherent superposition of bipartite entangled states written as

11 $${\left|\psi \right\rangle }_{ab}=\sqrt{{p}_{0}}{\left|{\phi }_{0}\right\rangle }_{ab}+\sqrt{{p}_{1}}{\left|{\phi }_{1}\right\rangle }_{ab}+\sqrt{{p}_{2}}{\left|{\phi }_{2}\right\rangle }_{ab},$$

where ${p}_{n}$, with $n\le 2$ and ${p}_{0}+{p}_{1}+{p}_{2}=1$, stands for the probability of generating a $2n$-photon entangled state of the form

12 $${|{\phi }_{n}\rangle }_{ab}=\frac{1}{n!\sqrt{n+1}}{({a}_{{\rm{h}}}^{\dagger }{b}_{{\rm{v}}}^{\dagger }-{a}_{{\rm{v}}}^{\dagger }{b}_{{\rm{h}}}^{\dagger })}^{n}{|0\rangle }_{ab}.$$

In Eq. (12), ${\left|0\right\rangle }_{ab}$ is the vacuum state and ${a}_{{\rm{h}}}^{\dagger }$ and ${a}_{{\rm{v}}}^{\dagger }$ (${b}_{{\rm{h}}}^{\dagger }$ and ${b}_{{\rm{v}}}^{\dagger }$) denote, respectively, the creation operators of horizontally and vertically polarised photons at the spatial mode $a$ ($b$). Remarkably, we set ${p}_{n}=0$ for $n\ge 3$ in Eq. (11). The underlying assumption is that the effect of multiple photon pairs is properly encompassed by the effect of double photon pairs, which is supported by our numerical simulations. A particular example of entanglement sources that sticks to the structure given by Eq. (12) are the parametric down conversion (PDC) sources^42,43^, and a thorough analysis of their performance for DIQKD is given in the Supplementary Information (considering a contribution of up to $n=3$ photon pairs). There, we show that PDC sources do not seem to be suitable for DIQKD, especially in the long-distance regime, since they require really long DIQKD sessions and deliver very low secret key rates.

For the evaluation of an entanglement source subject to Eq. (11), we characterise the photon-number statistics by means of two parameters alone: the probability ${p}_{0}$ of emitting vacuum, and the ratio $q={p}_{2}/{p}_{1}$ between the probability of emitting a double photon pair and that of emitting a single photon pair. Of course, if one considers a practical entanglement source, the photon-number statistics ${p}_{n}$ cannot be controlled separately, but they typically depend on an intensity parameter. For instance, in the case of PDC sources, we have that ${p}_{n}$ is fixed for all $n$ once we set the value of ${p}_{0}$ (or, equivalently, the intensity of the source), and in the low intensity regime the double-to-single photon pair ratio reads ${q}_{{\rm{P}}{\rm{D}}{\rm{C}}}={p}_{2}/{p}_{1}\approx (1-{p}_{0}-{p}_{1})/{p}_{1}=({p}_{0}^{-1/2}-1)({p}_{0}^{-1/2}+2)/2$. The case of ideal sources, on the other hand, corresponds to ${p}_{0}={p}_{2}=0$ and thus $q=0$. Due to the poor performance of PDC sources when used for DIQKD (see the Supplementary Information), below we consider combinations of $({p}_{0},q)$ that satisfy $0\le q < {q}_{{\rm{P}}{\rm{D}}{\rm{C}}}$ for the corresponding ${p}_{0} > 0$. In doing so, we investigate an intermediate scenario between the photon number statistics of ideal sources and those of PDC sources.

We remark, however, that the Supplementary Information includes a full mode analysis, both for the ESR and PQA architectures, which allows the evaluation of any desired photon number distribution for the different light sources, including the contribution of up to any wanted number of photon pairs per source.

The results for the simplified scenario discussed above are illustrated in Fig. 5, which shows the secret key rate as a function of the channel loss $\Lambda$ for ${n}_{{\rm{S}}{\rm{H}}}=1{0}^{9}$ and the security requirements given by ${S}_{1}$. Regarding the detector and coupling efficiency, we use ${\eta }_{{\rm{c}},{\rm{d}}}\in \{100{\rm{ \% }},98.7{\rm{ \% }}\}$. Note that the value ${\eta }_{{\rm{c}},{\rm{d}}}=96.5{\rm{ \% }}$ is not used here, as it is too close to the threshold efficiency with ideal sources. Instead, we choose ${\eta }_{{\rm{c}},{\rm{d}}}=98.7{\rm{ \% }}$, which in turn allows to simplify the comparison between this case and the one based on PDC sources in the Supplementary Information. For each value of ${\eta }_{{\rm{c}},{\rm{d}}}$, we plot three different cases. The first (second) case, assumes that the entanglement source ${\rho }_{bc}$ (${\rho }_{ab}$) is an ideal entanglement source, while ${\rho }_{ab}$ (${\rho }_{bc}$) is characterised by the parameters $({p}_{0},q)$. The third case considers that both ${\rho }_{bc}$ and ${\rho }_{ab}$ are characterised by the parameters $({p}_{0},q)$, which, for simplicity, we assume are the same for both sources. In general, though, the optimal intensity for each source will depend of the value of the channel loss. In each figure, we evaluate two possible values for ${p}_{0}$: ${p}_{0}=0.5$ (solid lines) and ${p}_{0}=0.9$ (dotted lines). Also, we consider four different values for the parameter $q\in {\mathscr{Q}}=\{0,1{0}^{-2},1{0}^{-1.5},1{0}^{-1}\}$.

**Figure 6:**
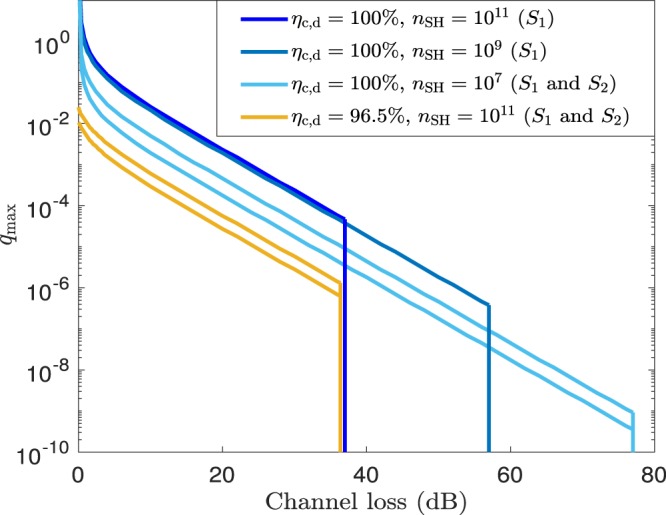
Upper bound on the maximum double-to-single photon pair ratio ${q}_{{\rm{m}}{\rm{a}}{\rm{x}}}$ of the entanglement source ${\rho }_{bc}$ required to achieve $K\ge 0$ with $\left\langle N\right\rangle \le 1{0}^{15}$, as a function of the channel loss $\Lambda$. Here, we set ${p}_{{\rm{d}}}=0$, so that the multiphotons generated in the qubit amplifier are the only source of noise in the system. The bluish lines use coupling and detection efficiency ${\eta }_{{\rm{c}},{\rm{d}}}=100{\rm{ \% }}$ and they include the block sizes ${n}_{{\rm{S}}{\rm{H}}}=1{0}^{11}$, $1{0}^{9}$ and $1{0}^{7}$, while the yellow lines use ${\eta }_{{\rm{c}},{\rm{d}}}=96.5{\rm{ \% }}$ and they only include the case ${n}_{{\rm{S}}{\rm{H}}}=1{0}^{11}$. This is so because ${n}_{{\rm{S}}{\rm{H}}}=1{0}^{9}$ and ${n}_{{\rm{S}}{\rm{H}}}=1{0}^{7}$ do not deliver a positive secret key rate for ${\eta }_{{\rm{c}},{\rm{d}}}=96.5{\rm{ \% }}$. Also, for each pair $({\eta }_{{\rm{c}},{\rm{d}}},{n}_{{\rm{S}}{\rm{H}}})$, the graphs corresponding to both sets of security settings, ${S}_{1}$ and ${S}_{2}$, are included whenever they are significantly different. Otherwise, we only plot that of ${S}_{1}$ for simplicity. The vertical cutoffs in the graphs indicate the points where $\left\langle N\right\rangle \approx 1{0}^{15}$. As expected, when ${\eta }_{{\rm{c}},{\rm{d}}}$ decreases the value of ${q}_{{\rm{m}}{\rm{a}}{\rm{x}}}$ decreases as well.

In all the plots within Fig. 5, if one compares the solid lines with the dotted lines, we observe that reducing the value of the probability ${p}_{0}$ for a fixed value of $q$ basically leads to a rigid increase of the secret key rate (*i.e*., an increase by a constant factor that keeps the slope of the curve in logarithmic scale). This is so because vacuum signals rarely lead to false heralding flags in the qubit amplifier: if ${\rho }_{ab}$ (${\rho }_{bc}$) emits a vacuum state, it is necessary that either ${\rho }_{bc}$ (${\rho }_{ab}$) emits more than one photon pair or that at least one dark count takes place at the detectors within the qubit amplifier in order to have a (spurious) successful heralding event. As a consequence, to a good extent, ${p}_{0}$ mainly affects the pre-factor ${P}_{{\rm{S}}{\rm{H}}}$, but not the conditional secret key rate, $K{|}_{{\rm{S}}{\rm{H}}}$. The greater the value of ${p}_{0}$, the smaller the value of ${P}_{{\rm{S}}{\rm{H}}}$, and thus the secret key rate rigidly decreases.

On the other hand, for a fixed value of ${p}_{0}$, increasing $q$ significantly affects $K{|}_{{\rm{S}}{\rm{H}}}$, so that multiple photon pairs are responsible for the changing slope of the secret key rate as well as for the position of the cutoff point where the secret key rate starts dropping down to zero, as shown in Fig. 5. This is so because multiple photon pairs lead to spurious heralding events that limit the utility of the qubit amplifier, and, as expected, this effect is amplified when the detection and coupling efficiency ${\eta }_{{\rm{c}},{\rm{d}}}$ decreases. In this regard, we also note that the performance of DIQKD seems to be more robust to the presence of multiple photon pairs in ${\rho }_{ab}$ than in ${\rho }_{bc}$. The reason goes as follows. Multi-photons arising from ${\rho }_{ab}$ need to undergo a lossy channel, but multi-photons from ${\rho }_{bc}$ do not. Therefore, the latter are more likely to trigger a spurious success at the qubit amplifier when the input from the channel is a vacuum signal. Actually, from Fig. 5 we observe that the curves with an ideal source ${\rho }_{bc}$, and ${\rho }_{ab}$ characterised with $q=1{0}^{-2}$ or $1{0}^{-1.5}$ are relatively close to the curve corresponding to $q=0$. This is so because the cutoff points of these curves at the high loss regime are due to the dark counts of the detectors at the qubit amplifier, as in the case $q=0$, and not to the presence of multiple photon pairs in ${\rho }_{ab}$. On the contrary, all the curves with an ideal source ${\rho }_{ab}$, and ${\rho }_{bc}$ characterised with a nonzero $q$, show an early cutoff point induced by the multiple photon pairs in ${\rho }_{bc}$.

Furthermore, we note that the cutoff points match for ${p}_{0}=0.5$ and ${p}_{0}=0.9$ if they are caused by the presence of multiple photon pairs, but they do not match if they are caused by the dark counts of the detectors. This is so because, in the former case, the cutoff point is roughly determined by the double-to-single photon pair ratio (*i.e*., by the parameter $q$), while in the latter case it is determined by the dark count to single photon pair ratio, which is different for each curve. Either way, Fig. 5 suggests that the noise induced by multiple photon pairs generated by the sources, particularly those generated by the sources within the qubit amplifier, seems to be the major challenge to achieve long-distance DIQKD with the considered setup.

This effect is investigated further in Fig. 6, where we plot an upper bound on the maximum value of the parameter $q$, which we denote by ${q}_{{\rm{m}}{\rm{a}}{\rm{x}}}$, to achieve $K\ge 0$ with $\left\langle N\right\rangle \le 1{0}^{15}$, as a function of the channel loss $\Lambda$. For this, we assume that the source ${\rho }_{ab}$ is an ideal source and we parametrise the source ${\rho }_{bc}$ with the quantity $q$. Note that since here we use the condition that the secret key rate is strictly greater than zero, we can set ${p}_{0}=0$ for ${\rho }_{bc}$ as well. This is so because, as already explained, setting ${p}_{0} > 0$ simply translates into a rigid decrease of the secret key rate, thus not affecting the value of ${q}_{{\rm{m}}{\rm{a}}{\rm{x}}}$. That is, we define ${q}_{{\rm{m}}{\rm{a}}{\rm{x}}}={min}_{{p}_{1}}\{(1-{p}_{1})/{p}_{1}|K\ge 0\}$. For illustration purposes, we consider the extreme cases ${\eta }_{{\rm{c}},{\rm{d}}}=100{\rm{ \% }}$ and ${\eta }_{{\rm{c}},{\rm{d}}}=96.5{\rm{ \% }}$ again, and in addition we set ${p}_{{\rm{d}}}=0$ in order to investigate the limitations imposed by the noise due to multiple photon pairs alone. In this scenario, Fig. 6 suggests that, irrespectively of the block size ${n}_{{\rm{S}}{\rm{H}}}$, the value of the detector efficiency ${\eta }_{{\rm{c}},{\rm{d}}}$ and the security settings, the double-to-single photon pair ratio $q$ severely restricts the maximum distance that is achievable with DIQKD. Moreover, note that in a realistic situation with a non-ideal ${\rho }_{ab}$, ${q}_{{\rm{m}}{\rm{a}}{\rm{x}}}$ would be lower than the value shown in Fig. 6. The vertical cutoffs in the graphs indicate the points where $\left\langle N\right\rangle \approx 1{0}^{15}$, as this value is already probably too large for a QKD session today. Since ${q}_{{\rm{m}}{\rm{a}}{\rm{x}}}$ is very small at these cutoff points, the corresponding values of $\Lambda$ are very close (indistinguishable to our numerical precision) to those of the case $q=0$, which are given by

13 $$\Lambda =150-10{\log }_{10}(\frac{2{n}_{{\rm{S}}{\rm{H}}}}{{\eta }_{{\rm{c}},{\rm{d}}}^{4}})$$

**Figure 7:**
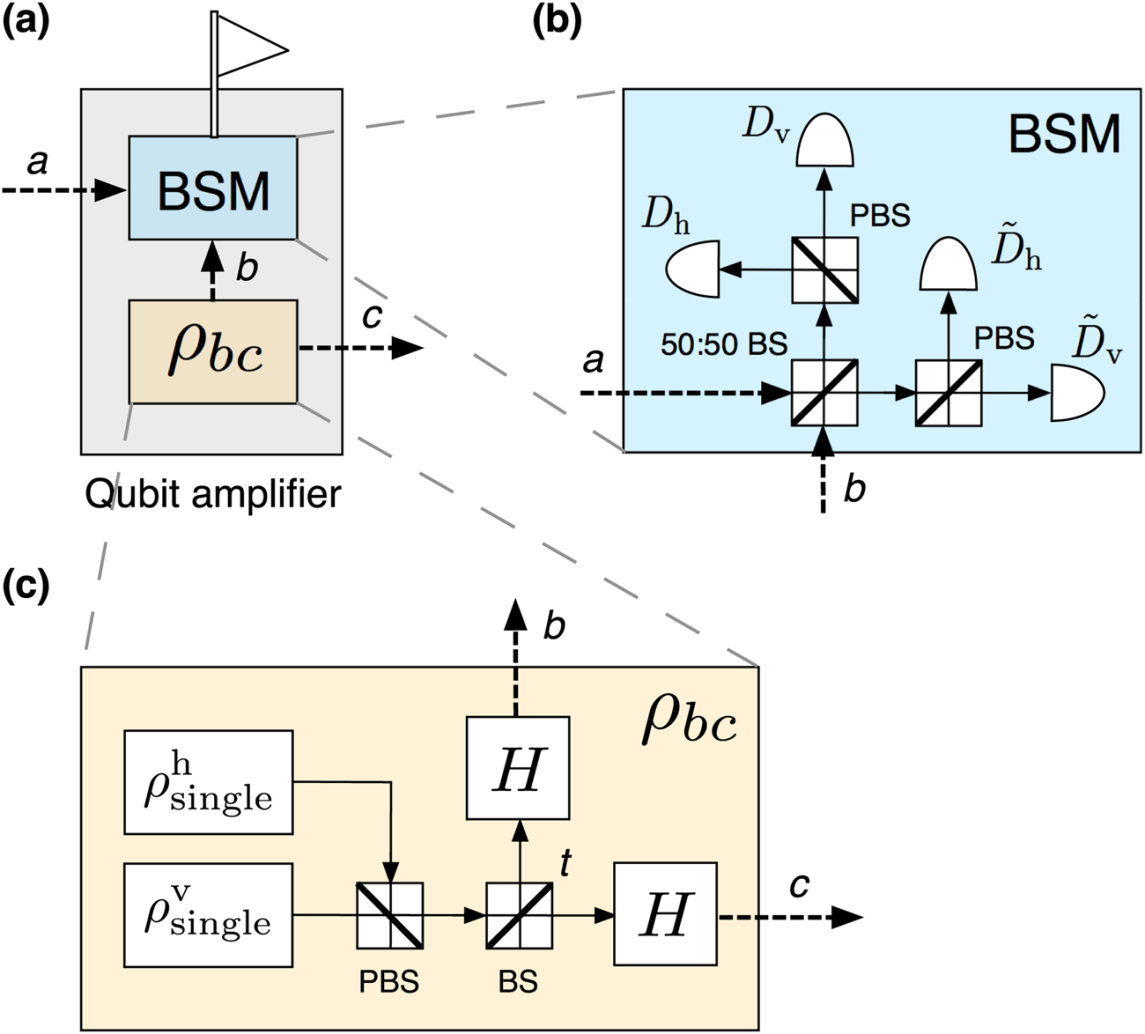
(**a**) Working principle of an heralded qubit amplifier based on teleportation^34–36^. A successful heralding is indicated with a flag. It notifies that a photon at the input port, $a$, of the qubit amplifier was teleported to a photon at its output port, $c$. For this, the qubit amplifier first generates a bipartite entangled state, ${\rho }_{bc}$, and then measures the signals in modes $a$ and $b$ with a BSM. In doing so, the state of the photon at mode $a$ is teleported to that at mode $c$ up to a unitary rotation. The only difference between the qubit amplifiers proposed in^34–36^ is the mechanism to generate the entangled states ${\rho }_{bc}$. See the main text for further details. (**b**) Linear-optics BSM. The input states in modes $a$ and $b$ interfere at a 50:50 beamsplitter (BS). A polarizing BS (PBS) located at each output port of the BS separates vertically and horizontally polarised photons. Here we shall assume that all detectors are PNR detectors. A successful BSM corresponds to detecting two photons with orthogonal polarizations, *i.e*., only when exactly two detectors record one input photon each for any of the following photodetector pairs: $\left({D}_{h},{D}_{v}\right)$, $({D}_{h},{\widetilde{D}}_{v})$, $({\widetilde{D}}_{h},{D}_{v})$ or $({\widetilde{D}}_{h},{\widetilde{D}}_{v})$. (**c**) Scheme introduced in^35^ to generate ${\rho }_{bc}$. A light source emits horizontally (vertically) polarised single-photons ${\rho }_{{\rm{s}}{\rm{i}}{\rm{n}}{\rm{g}}{\rm{l}}{\rm{e}}}^{h}$ (${\rho }_{{\rm{s}}{\rm{i}}{\rm{n}}{\rm{g}}{\rm{l}}{\rm{e}}}^{v}$), which interfere at a PBS and then go through a BS of tunable transmittance $t$. Two Hadamard gates, denoted by $H$ in the figure, are used to avoid (if one disregards noise effects) that input vacuum signals at mode $a$ can produce a successful heralding flag when the BSM is that given by (**b)**.

for the different pairs $({\eta }_{{\rm{c}},{\rm{d}}},{n}_{{\rm{S}}{\rm{H}}})$. This expression is directly obtained from Eq. (9) assuming ${p}_{{\rm{d}}}=0$. As a final remark, note that one might achieve a source whose parameter $q < {q}_{{\rm{m}}{\rm{a}}{\rm{x}}}$ for a given distance by simply decreasing the intensity of the source. Indeed, this is the case, for example, of PDC sources, where one can reduce $\lambda$ and thus $q$ at the price of significantly increasing the probability ${p}_{0}$ of emitting vacuum. While this might provide a positive key rate according to Fig. 6 (by assuming still that ${\rho }_{ab}$ is an ideal source), the resulting secret key rate might be probably too low to be practical because the probability of having a successful heralding event would be very low. The situation gets worse in the presence of dark counts.

## Discussion

Device independence is a desirable feature for quantum key distribution (QKD) to ultimately defeat quantum hacking. However, it comes at a high price, in terms of achievable performance and required resources. Indeed, long distance device-independent QKD (DIQKD) requires the use of heralding devices, like for instance qubit amplifiers, which can herald the arrival of a photon and thus decouple channel loss from the measurement settings selection.

In this work, we have investigated all-photonic DIQKD assisted by two general types of qubit amplifiers—entanglement swapping relays and polarization qubit amplifiers—in the finite-key regime. In doing so, we have quantified some crucial experimental parameters that are essential to achieve DIQKD over practical distances and within a reasonable time frame of signal transmission. This includes, for example, the minimum value of the detection efficiency of the photodetectors and the quality of the entanglement light sources, in terms of their vacuum and multi-photon contributions. In this regard, we have shown that, even if perfect entanglement sources and photon-number-resolving detectors were available, the ability to achieve large enough violations of a loophole-free CHSH test within a DIQKD session of a reasonable time duration already imposes very strong restrictions on the minimum detection efficiency ($\gtrsim 96,5 \%$), which further increases quickly with the length of the transmission link. Similarly, we have shown that multi-photon pulses emitted by practical entanglement sources have a severe effect on the performance of DIQKD assisted by qubit amplifiers, as multiple photon pairs lead to spurious heralding events that strongly decrease the conditional secret key rate.

Altogether, our results suggest that the possibility of implementing optical DIQKD over long distances using the considered qubit amplifier architectures is probably quite far-off, as it seems to require a significant improvement of our current experimental capabilities.

## Methods

### Device models

In this section we briefly introduce the mathematical models that we used to derive the results exposed in Sec. II These models describe the main optical devices employed in a photonic implementation of a DIQKD setup, together with the behaviour of a typical lossy quantum channel.

#### Photodetectors

We consider that Alice and Bob have photon-number-resolving (PNR) detectors at their disposal, which are able to count the number of photons contained in each incoming optical pulse. In the relevant regime of low noise, they can be described by a positive operator valued measure (POVM) with the following elements:

14 $${\Pi }_{k}=\{\begin{array}{cc}(1-{p}_{{\rm{d}}}){\mathop{\Pi }\limits^{ \sim }}_{0} & \mathrm{if}\,k=0,\\ (1-{p}_{{\rm{d}}}){\mathop{\Pi }\limits^{ \sim }}_{k}+{p}_{{\rm{d}}}{\mathop{\Pi }\limits^{ \sim }}_{k-1} & \mathrm{if}\,k\ge 1,\end{array}$$

where the quantity ${p}_{{\rm{d}}}$ stands for the dark count rate of the photodetectors, which is, to a good approximation, independent of the incoming signals. On the other hand, the operators ${\widetilde{\Pi }}_{k}$ that appear in Eq. (14), with $k\in {\mathbb{N}}$, are given by

15 $${\mathop{\Pi }\limits^{ \sim }}_{k}={\sum }_{j=k}^{{\rm{\infty }}}(\genfrac{}{}{0.0pt}{}{j}{k}){\eta }_{{\rm{d}}}^{k}{(1-{\eta }_{{\rm{d}}})}^{j-k}|\,j\rangle \langle \,j\,|,$$

with ${\eta }_{{\rm{d}}}$ denoting the detection efficiency of the detectors, and where $\left|\,j\right\rangle$ stands for a Fock state with $j$ photons.

We remark that the mathematical model given by Eq. (14) assumes, for simplicity, that dark counts can only increment by one unit the number of photons observed in a given pulse. That is, if an optical pulse contains, say, $k$ photons, we assume that the measurement outcome is at most $k+1$ photons due to the dark counts, but not greater than this. This is a fair approximation given that ${p}_{{\rm{d}}}$ is sufficiently low, which indeed is typically the case in practice.

#### Heralded qubit amplifiers

To achieve long-distance DIQKD, we assume that Alice and Bob use heralded qubit amplifiers^34–36^ to notify them the arrival of a photon before they select their measurement settings. That is, only after the qubit amplifier confirms that a photon has arrived, Alice (Bob) selects the measurement and measures the photon. If no successful heralding takes place, the optical pulse is simply discarded.

Typical qubit amplifiers consist in a teleportation gate^44^. That is, a successful heralding occurs when the state of the arriving photon is teleported to a photon at the output port of the qubit amplifier. The general mechanism is depicted in Fig. 7(a), while Fig. 7(b) shows the standard linear-optics BSM used by the qubit amplifiers introduced in^34–36^ to teleport the input photon. More efficient BSMs exist^45,46^ and could be used here as well, although they require complicated entangled ancilla states. Depending on the mechanism used to generate the bipartite entangled states, ${\rho }_{bc}$, illustrated in Fig. 7(a), one can distinguish two types of qubit amplifiers: polarization qubit amplifiers (PQAs)^34,35^ and entanglement swapping relays (ESRs)^36^.

PQAs, first introduced in^34^ based on the seminal work reported in^47^, typically employ practical single-photon sources to generate ${\rho }_{bc}$. For instance, the PQA proposed in^35^ uses the linear-optics circuit shown in Fig. 7(c) for this purpose, where ${\rho }_{{\rm{s}}{\rm{i}}{\rm{n}}{\rm{g}}{\rm{l}}{\rm{e}}}^{{\rm{h}}}$ (${\rho }_{{\rm{s}}{\rm{i}}{\rm{n}}{\rm{g}}{\rm{l}}{\rm{e}}}^{{\rm{v}}}$) represents the state of a single-photon pulse prepared in horizontal (vertical) polarization. In the ideal case of perfect single-photon sources and unit detection and coupling efficiencies, it is straightforward to show that the circuit given by Fig. 7(c) generates states ${\rho }_{bc}={\left|\phi \right\rangle }_{bc}\left\langle \phi \,\right|$ with

16 $${\left|\phi \right\rangle }_{bc}=(1-t){\left|\psi \right\rangle }_{bb}+t{\left|\varphi \right\rangle }_{cc}-\sqrt{2t(1-t)}{\left|\chi \right\rangle }_{bc},$$

where the parameter $t$ is the transmittance of the tunable beamsplitter (BS) illustrated in Fig. 7(c), and the states ${\left|\psi \right\rangle }_{bb}$, ${\left|\varphi \right\rangle }_{cc}$, and ${\left|\chi \right\rangle }_{bc}$ have the form

17 $$\begin{array}{ccc}{|\psi \rangle }_{bb} & = & \frac{1}{2}({{b}_{{\rm{h}}}^{\dagger }}^{2}-{{b}_{{\rm{v}}}^{\dagger }}^{2}){|0\rangle }_{b},\\ {|\varphi \rangle }_{cc} & = & \frac{1}{2}({{c}_{{\rm{h}}}^{\dagger }}^{2}-{{c}_{{\rm{v}}}^{\dagger }}^{2}){|0\rangle }_{c},\\ {|\chi \rangle }_{bc} & = & \frac{1}{\sqrt{2}}({b}_{{\rm{h}}}^{\dagger }{c}_{{\rm{h}}}^{\dagger }-{b}_{{\rm{v}}}^{\dagger }{c}_{{\rm{v}}}^{\dagger }){|0\rangle }_{bc}.\end{array}$$

In Eq. (17), the states ${\left|0\right\rangle }_{b}$, ${\left|0\right\rangle }_{c}$ and ${\left|0\right\rangle }_{bc}$ denote the vacuum states of the corresponding modes. The expression for the output states of the PQA in the practical scenario with non-ideal sources and non-unit detector and coupling efficiencies can be found in the Supplementary Information.

Let us continue assuming, for simplicity and for the moment, an ideal scenario where the BSM within the qubit amplifier uses perfect PNR detectors (*i.e*., ${p}_{{\rm{d}}}=0$ and ${\eta }_{{\rm{d}}}=1$ in Eqs. 14 and 15) and lossless BSs. Then, from Eq. (16), it can be shown that whenever a single-photon pulse prepared in, say, the pure state ${|{\varphi }_{{\rm{i}}{\rm{n}}}\rangle }_{a}=(\alpha {a}_{{\rm{h}}}^{\dagger }+\beta {a}_{{\rm{v}}}^{\dagger }){|0\rangle }_{a}$ (with $| \alpha {| }^{2}+| \beta {| }^{2}=1$) arrives at the input port $a$ of the qubit amplifier, a successful BSM occurs with probability $t(1-t)$. Also, in the case of a successful result, the state of the output photon at mode $c$ (after applying an appropriate unitary transformation) is equal to ${|{\varphi }_{{\rm{o}}{\rm{u}}{\rm{t}}}\rangle }_{c}=(\alpha {c}_{{\rm{h}}}^{\dagger }+\beta {c}_{{\rm{v}}}^{\dagger }){|0\rangle }_{c}$. That is, the state ${|{\varphi }_{{\rm{i}}{\rm{n}}}\rangle }_{a}$ of the input photon is successfully teleported to an output photon. On the other hand, if a vacuum pulse, ${|{\varphi }_{{\rm{i}}{\rm{n}}}\rangle }_{a}={|0\rangle }_{a}$, arrives at the input port $a$ of the qubit amplifier, this state can never lead to a spurious heralding event, at least in the ideal scenario. This is so because, when the BSM uses perfect PNR detectors with no dark counts, the state ${\left|0\right\rangle }_{a}{\left|\phi \right\rangle }_{bc}$, with ${\left|\phi \right\rangle }_{bc}$ given by Eq. (16), cannot produce two detection clicks associated to orthogonal polarizations if it is measured with the BSM shown in Fig. 7(b).

Finally, qubit amplifiers based on ESRs^36^ directly prepare the state ${\rho }_{bc}$ with practical entanglement light sources like, for example, PDC sources. Indeed, in contrast to the arguments presented in^34,48^, it was shown in^36^ that when this type of qubit amplifier is used in DIQKD, it can provide higher secret key rates than those achievable with the PQA introduced in^34^ when using PDC sources.

#### Optical couplers

Our analysis in Sec. II considers a fiber-based implementation of DIQKD. Thus, we model the coupling of the photons generated by the light sources into the optical fibers by means of a BS of transmittance ${\eta }_{{\rm{c}}}$. One input to the BS is the quantum signal, while the other input is a vacuum state. Similarly, one of the outputs of the BS is the optical fiber, while we assume that the other output is not accessible and represents the loss.

#### Quantum channel

For simplicity, we suppose that the quantum channel mainly introduces loss. That is, we disregard any noise effect due for example to polarization or phase misalignment.

The channel loss is modeled with a BS of transmittance ${\eta }_{{\rm{c}}{\rm{h}}}=1{0}^{-\Lambda /10}$, where the parameter $\Lambda$ (dB) is related to the transmission distance $L$ (km) by an attenuation coefficient $\alpha$ (dB/km) via the expression $\Lambda =\alpha L$. The specific value of $\alpha$ depends on the considered channel. For instance, a typical value for $\alpha$ in the case of single-mode optical fibers in the telecom wavelength is $\alpha =0.2$ dB/km.

### Secret key length

We use the security analysis introduced in^28^, which is valid against coherent attacks. Prior to the execution of the protocol, Alice and Bob agree on three parameters that tag the security of the final keys, ${K}_{{\rm{A}}}$ and ${K}_{{\rm{B}}}$. These parameters are the secrecy parameter, ${\epsilon }_{{\rm{s}}{\rm{e}}{\rm{c}}}$, the correctness parameter, ${\epsilon }_{{\rm{c}}{\rm{o}}{\rm{r}}}$, and the robustness parameter, ${\epsilon }_{{\rm{r}}{\rm{o}}{\rm{b}}}$.

In particular, a protocol is said to be ${\epsilon }_{{\rm{s}}{\rm{e}}{\rm{c}}}$-secret, when implemented using a device D, if it satisfies

18 $$[1-P(\text{abort})]||{\rho }_{{K}_{\text{A}}E}-{\rho }_{{U}_{l}}\otimes {\rho }_{E}|{|}_{1}\le {\epsilon }_{\sec },$$

where $P(\text{abort})$ is the abortion probability of the protocol, $| | \rho | {| }_{1}=\sqrt{\rho {\rho }^{\dagger }}$ stands for the trace norm, *E* is a quantum register held by the eavesdropper that may be initially correlated with D, ${\rho }_{{K}_{A}E}$ is the output state of the DIQKD protocol describing Alice’s key string ${K}_{{\rm{A}}}$ and the quantum register E conditioned on not aborting, ${\rho }_{{U}_{l}}={\sum }_{z}\frac{1}{{2}^{l}}{|z\rangle }_{{\rm{A}}}\langle z\,|$ is the uniform mixture of all possible values of a $l$-bit string ${K}_{{\rm{A}}}$, and ${\rho }_{{U}_{l}}\otimes {\rho }_{E}$ is the perfectly secret output state.

The parameter ${\epsilon }_{{\rm{s}}{\rm{e}}{\rm{c}}}$ is upper bounded by

19 $${\epsilon }_{{\rm{s}}{\rm{e}}{\rm{c}}}\le {\epsilon }_{{\rm{P}}{\rm{A}}}+{\epsilon }_{{\rm{s}}}+{\epsilon }_{{\rm{E}}{\rm{A}}},$$

where ${\epsilon }_{{\rm{P}}{\rm{A}}}+{\epsilon }_{{\rm{s}}}$ is the total failure probability associated to the privacy amplification step, ${\epsilon }_{{\rm{P}}{\rm{A}}}$ being an upper bound on the error probability of the randomness extractor and ${\epsilon }_{{\rm{s}}}$ being the smoothing parameter of the ${\epsilon }_{{\rm{s}}}$-smooth min-entropy^28^. The term ${\epsilon }_{{\rm{E}}{\rm{A}}}$ is the failure probability associated to the entropy accumulation theorem^30^, which only guarantees that a certain lower bound on the ${\epsilon }_{{\rm{s}}}$-smooth min-entropy holds with a probability larger than $1-{\epsilon }_{{\rm{E}}{\rm{A}}}$.

The correctness parameter, ${\epsilon }_{{\rm{c}}{\rm{o}}{\rm{r}}}$, quantifies the probability that the final keys, ${K}_{{\rm{A}}}$ and ${K}_{{\rm{B}}}$, are not equal. More precisely, a protocol is said to be ${\epsilon }_{{\rm{c}}{\rm{o}}{\rm{r}}}$-correct if $P[{K}_{{\rm{A}}}\ne {K}_{{\rm{B}}}]\le {\epsilon }_{{\rm{c}}{\rm{o}}{\rm{r}}}$. According to the protocol definition given in the previous section, we have that ${\epsilon }_{{\rm{c}}{\rm{o}}{\rm{r}}}={\epsilon }_{{\rm{I}}{\rm{R}}}$.

Finally, a protocol is said to be ${\epsilon }_{{\rm{r}}{\rm{o}}{\rm{b}}}$-robust for a specific honest implementation (*i.e*., for a particular implementation where the eavesdropper does not intervene) if it aborts with a probability smaller than ${\epsilon }_{{\rm{r}}{\rm{o}}{\rm{b}}}$. The protocol described above can only abort in two steps: the information reconciliation step, and the parameter estimation step. Therefore, we have that ${\epsilon }_{{\rm{r}}{\rm{o}}{\rm{b}}}$ satisfies

20 $${\epsilon }_{{\rm{r}}{\rm{o}}{\rm{b}}}\le {\epsilon }_{{\rm{r}}{\rm{o}}{\rm{b}}}^{{\rm{I}}{\rm{R}}}+{\epsilon }_{{\rm{r}}{\rm{o}}{\rm{b}}}^{{\rm{P}}{\rm{E}}},$$

where ${\epsilon }_{{\rm{r}}{\rm{o}}{\rm{b}}}^{{\rm{I}}{\rm{R}}}$ (${\epsilon }_{{\rm{r}}{\rm{o}}{\rm{b}}}^{{\rm{P}}{\rm{E}}}$) is the probability of aborting at the information reconciliation (parameter estimation) step for the considered honest implementation. Moreover, we have that the quantity ${\epsilon }_{{\rm{r}}{\rm{o}}{\rm{b}}}^{{\rm{P}}{\rm{E}}}$ verifies

21 $${\epsilon }_{{\rm{r}}{\rm{o}}{\rm{b}}}^{{\rm{P}}{\rm{E}}}\le {\epsilon }_{{\rm{r}}{\rm{o}}{\rm{b}}}^{{\rm{E}}{\rm{A}}}+{\epsilon }_{{\rm{I}}{\rm{R}}},$$

where ${\epsilon }_{{\rm{r}}{\rm{o}}{\rm{b}}}^{{\rm{E}}{\rm{A}}}$ is the probability of the fraction of CHSH wins, ${C}_{{\rm{S}}{\rm{H}}}/{n}_{{\rm{S}}{\rm{H}}}$, being lower than the threshold $\omega {|}_{{\rm{S}}{\rm{H}}}\gamma -{\delta }_{{\rm{e}}{\rm{s}}{\rm{t}}}$. That is,

22 $${\epsilon }_{{\rm{r}}{\rm{o}}{\rm{b}}}^{{\rm{E}}{\rm{A}}}=P(\omega {|}_{{\rm{S}}{\rm{H}}}\gamma -\frac{{C}_{{\rm{S}}{\rm{H}}}}{{n}_{{\rm{S}}{\rm{H}}}} > {\delta }_{{\rm{e}}{\rm{s}}{\rm{t}}}).$$

Note that for fixed values of ${\epsilon }_{{\rm{r}}{\rm{o}}{\rm{b}}}^{{\rm{E}}{\rm{A}}}$ and ${n}_{{\rm{S}}{\rm{H}}}$, the minimum value of ${\delta }_{{\rm{e}}{\rm{s}}{\rm{t}}}$ such that Eq. (22) holds satisfies^49^

23 $${\delta }_{{\rm{e}}{\rm{s}}{\rm{t}}}\ge \sqrt{\frac{1}{2{n}_{{\rm{S}}{\rm{H}}}}\mathrm{ln}(\frac{1}{{\epsilon }_{{\rm{r}}{\rm{o}}{\rm{b}}}^{{\rm{E}}{\rm{A}}}})}.$$

Also, we note that the parameter ${\epsilon }_{{\rm{I}}{\rm{R}}}$ contributes to ${\epsilon }_{{\rm{r}}{\rm{o}}{\rm{b}}}^{{\rm{P}}{\rm{E}}}$ in Eq. (21) because, conditioned on not aborting the protocol in the error verification step, Bob performs the parameter estimation step by using his original bit string of outcomes, $B$, and his estimate, ${Z}_{{\rm{B}}}$, of Alice’s string, which is equal to ${Z}_{{\rm{A}}}$ except with probability ${\epsilon }_{{\rm{I}}{\rm{R}}}$.

A list with the main parameters related to the security of the protocol is provided in Table 4.

**Table 4:** List containing the main finite-key security parameters.

	Finite-key security parameters
${\epsilon }_{{\rm{s}}{\rm{e}}{\rm{c}}}$	Secrecy parameter
${\epsilon }_{{\rm{c}}{\rm{o}}{\rm{r}}}$	Correctness parameter
${\epsilon }_{{\rm{r}}{\rm{o}}{\rm{b}}}$	Robustness parameter
${\epsilon }_{{\rm{s}}}$	Smoothing parameter of the min-entropy
${\epsilon }_{{\rm{r}}{\rm{o}}{\rm{b}}}^{{\rm{P}}{\rm{E}}}$	Abortion probability of parameter estimation
${\epsilon }_{{\rm{r}}{\rm{o}}{\rm{b}}}^{{\rm{I}}{\rm{R}}}$	Abortion probability of information reconciliation
${\epsilon }_{{\rm{r}}{\rm{o}}{\rm{b}}}^{{\rm{E}}{\rm{A}}}$	Abortion probability of entropy accumulation
${\epsilon }_{{\rm{E}}{\rm{A}}}$	Error probability of the entropy accumulation bound

Then, it turns out that, conditioned on not aborting, the DIQKD protocol presented in the Results section delivers ${\epsilon }_{{\rm{c}}{\rm{o}}{\rm{r}}}$-correct and ${\epsilon }_{{\rm{s}}{\rm{e}}{\rm{c}}}$-secret output keys, ${K}_{{\rm{A}}}$ and ${K}_{{\rm{B}}}$, whose length, $l$, is given by

24 $$\begin{array}{ccc}l & = & {n}_{{\rm{S}}{\rm{H}}}({\eta }_{{\rm{o}}{\rm{p}}{\rm{t}}}-\gamma )-2\log 7\sqrt{1-2\log [\frac{{\epsilon }_{{\rm{s}}}}{4}({\epsilon }_{{\rm{E}}{\rm{A}}}+{\epsilon }_{{\rm{I}}{\rm{R}}})]}\\  &  & \times \sqrt{{n}_{{\rm{S}}{\rm{H}}}}-lea{k}_{{\rm{I}}{\rm{R}}}-3\log [1-\sqrt{1-{(\frac{{\epsilon }_{{\rm{s}}}}{4})}^{2}}]\\  &  & -2\log \frac{1}{{\epsilon }_{{\rm{P}}{\rm{A}}}}.\end{array}$$

Here, the term ${\eta }_{{\rm{o}}{\rm{p}}{\rm{t}}}$ represents a lower bound on the entropy generation rate of the CHSH game,

25 $${\eta }_{{\rm{o}}{\rm{p}}{\rm{t}}}(\omega {|}_{{\rm{S}}{\rm{H}}},{n}_{{\rm{S}}{\rm{H}}},\gamma ,{\delta }_{{\rm{e}}{\rm{s}}{\rm{t}}},{\epsilon }_{{\rm{s}}}/4,{\epsilon }_{{\rm{E}}{\rm{A}}}+{\epsilon }_{{\rm{I}}{\rm{R}}})={max}_{\frac{3}{4} < {p}_{t} < \frac{2+\sqrt{2}}{4}}\eta (\frac{\omega {|}_{{\rm{S}}{\rm{H}}}\gamma -{\delta }_{{\rm{e}}{\rm{s}}{\rm{t}}}}{\gamma },{p}_{t},{n}_{{\rm{S}}{\rm{H}}},\gamma ,{\epsilon }_{{\rm{s}}}/4,{\epsilon }_{{\rm{E}}{\rm{A}}}+{\epsilon }_{{\rm{I}}{\rm{R}}}),$$

where $\eta (p,{p}_{t},{n}_{{\rm{S}}{\rm{H}}},\gamma ,{\epsilon }_{1},{\epsilon }_{2})$ has the form

26 $$\begin{array}{ccc}\eta (p,{p}_{t},{n}_{{\rm{S}}{\rm{H}}},\gamma ,{\epsilon }_{1},{\epsilon }_{2}) & = & {f}_{min}(p,{p}_{t})-\frac{2}{\sqrt{{n}_{{\rm{S}}{\rm{H}}}}}\\  &  & \times [\log 13+{\frac{1}{\gamma }\frac{dg(p)}{dp}|}_{{p}_{t}}]\\  &  & \times \sqrt{1-2\log ({\epsilon }_{1}{\epsilon }_{2})}.\end{array}$$

In this equation, ${f}_{{\rm{m}}{\rm{i}}{\rm{n}}}(p,{p}_{t})$ is given by

27 $${f}_{{\rm{m}}{\rm{i}}{\rm{n}}}(p,{p}_{t})=\{\begin{array}{cc}g(p) & p < {p}_{t}\\ {g({p}_{t})+\frac{dg(p)}{dp}|}_{{p}_{t}}(p-{p}_{t}) & p\ge {p}_{t},\end{array}$$

and the function $g(p)$ reads

28 $$g(p)=1-h\left[\frac{1}{2}+\frac{1}{2}\sqrt{16p(p-1)+3}\right],$$

where the winning probability $p$ lies in the interval $0\le p\le (2+\sqrt{2})/4$. Similarly, $h(x)$ is the binary entropy function, $h(x)=-\,x\log x-(1-x)\log (1-x)$.

On the other hand, the information reconciliation leakage term in 24, $lea{k}_{{\rm{I}}{\rm{R}}}$, depends not only on the expected conditional quantum bit error rate of the key rounds, $Q{|}_{{\rm{S}}{\rm{H}}}$, but also on the expected conditional winning rate of the test rounds, $\omega {|}_{{\rm{S}}{\rm{H}}}$. It can be written as

29 $$\begin{array}{ccc}lea{k}_{{\rm{I}}{\rm{R}}}(Q{|}_{{\rm{S}}{\rm{H}}},\omega {|}_{{\rm{S}}{\rm{H}}},{n}_{{\rm{S}}{\rm{H}}},\gamma ,{\epsilon }_{{\rm{I}}{\rm{R}}},{\epsilon }_{{\rm{r}}{\rm{o}}{\rm{b}}}^{{\rm{I}}{\rm{R}}}) & = & {n}_{{\rm{S}}{\rm{H}}}[(1-\gamma )h(Q{|}_{{\rm{S}}{\rm{H}}})+\gamma h(\omega {|}_{{\rm{S}}{\rm{H}}})]\\  &  & +4\sqrt{{n}_{{\rm{S}}{\rm{H}}}}\log (2\sqrt{2}+1)\sqrt{2\log [\frac{8}{{{\epsilon }^{{\rm{^{\prime} }}}}_{{\rm{I}}{\rm{R}}}^{2}}]}\\  &  & +\log [\frac{8}{{{\epsilon }^{{\rm{^{\prime} }}}}_{IR}^{2}}+\frac{2}{2-{{\epsilon }^{{\rm{^{\prime} }}}}_{IR}}]+\log (\frac{1}{{\epsilon }_{{\rm{I}}{\rm{R}}}}),\end{array}$$

where $${\epsilon }_{{\rm{r}}{\rm{o}}{\rm{b}}}^{{\rm{I}}{\rm{R}}}={{\epsilon }^{{\rm{^{\prime} }}}}_{{\rm{I}}{\rm{R}}}+{\epsilon }_{{\rm{I}}{\rm{R}}}$$.

Recently, a slightly improved bound on the smooth min-entropy was derived for the entropy accumulation theorem^31^, thus enabling an improvement of the secret key length given by Eq. (24). However, the results reported in^29,31^ suggest that such improvement is small and does not probably have a significant impact in the regime of block sizes required by practical DIQKD.

Finally, in an actual execution of the protocol that required the transmission of $N$ signals, the secret key rate is defined as

30 $$K=\frac{l}{N}.$$

We remark that, in the limit where ${n}_{{\rm{S}}{\rm{H}}}\to {\rm{\infty }}$, Eq. (30) matches the asymptotic secret key rate against general attacks reported in^17^, which is given by

31 $$\begin{array}{ccc}{K}_{{\rm{\infty }}} & = & {lim}_{{n}_{{\rm{S}}{\rm{H}}}\to {\rm{\infty }}}\frac{{n}_{{\rm{S}}{\rm{H}}}{\eta }_{{\rm{o}}{\rm{p}}{\rm{t}}}-lea{k}_{{\rm{I}}{\rm{R}}}}{N}\\  & = & {P}_{{\rm{S}}{\rm{H}}}\{1-h[\frac{1}{2}+\frac{1}{2}\sqrt{16\omega {|}_{{\rm{S}}{\rm{H}}}(\omega {|}_{{\rm{S}}{\rm{H}}}-1)+3}]\\  &  & -h(Q{|}_{{\rm{S}}{\rm{H}}})\}.\end{array}$$

## Supplementary information

Supplementary Information

## Data Availability

No datasets were generated or analysed during the current study.
