# Supplementary material for: Long-distance device-independent quantum key distribution
Source: Sci Rep. 2019 Nov 28;9:17749. doi: 10.1038/s41598-019-53803-0 (PMC6882898; doi:10.1038/s41598-019-53803-0)
Supplement: Supplementary file 1 — Supplementary Information [file 41598_2019_53803_MOESM1_ESM.pdf]

Long-distance device-independent quantum key distribution

Víctor Zapatero* and Marcos Curty

I. HONEST IMPLEMENTATIONS

Here, we present the main calculations needed to reproduce our simulation results. For this, we use the mathematical models introduced in the main text. As mentioned there, we model loss (*e.g.* occurring in the optical couplers or the fiber link) with a beamsplitter (BS) described by a unitary transformation of the form

$$\begin{aligned} a_{\mathrm{h,v}}^{\dagger} &\to \sqrt{\eta} c_{\mathrm{h,v}}^{\dagger} + \sqrt{1-\eta} d_{\mathrm{h,v}}^{\dagger}, \\ b_{\mathrm{h,v}}^{\dagger} &\to \sqrt{\eta} d_{\mathrm{h,v}}^{\dagger} - \sqrt{1-\eta} c_{\mathrm{h,v}}^{\dagger}, \end{aligned} \tag{1}$$

where the input mode a is the quantum signal, the input mode b is a vacuum state, the output mode c is the optical fiber, and the output mode d represents loss. In Eq. (1), the subscript "h,v" indicates again horizontal and vertical polarization respectively. That is, the BS transformation given by Eq. (1) applies to both polarizations.

Similarly, polarization modulators are simply described by a rotation that transforms the input modes $a_{\mathrm{h}}^{\dagger}$ and $a_{\mathrm{v}}^{\dagger}$ as follows

$$\begin{aligned} a_{\mathrm{h}}^{\dagger} &\to \cos\theta b_{\mathrm{h}}^{\dagger} + \sin\theta b_{\mathrm{v}}^{\dagger}, \\ a_{\mathrm{v}}^{\dagger} &\to \cos\theta b_{\mathrm{v}}^{\dagger} - \sin\theta b_{\mathrm{h}}^{\dagger}, \end{aligned} \tag{2}$$

where $b_{\mathrm{h}}^{\dagger}$ and $b_{\mathrm{v}}^{\dagger}$ denote the output modes. The case $\theta = \pi/4$ corresponds to the Hadamard transformation.

A. Entanglement swapping relay

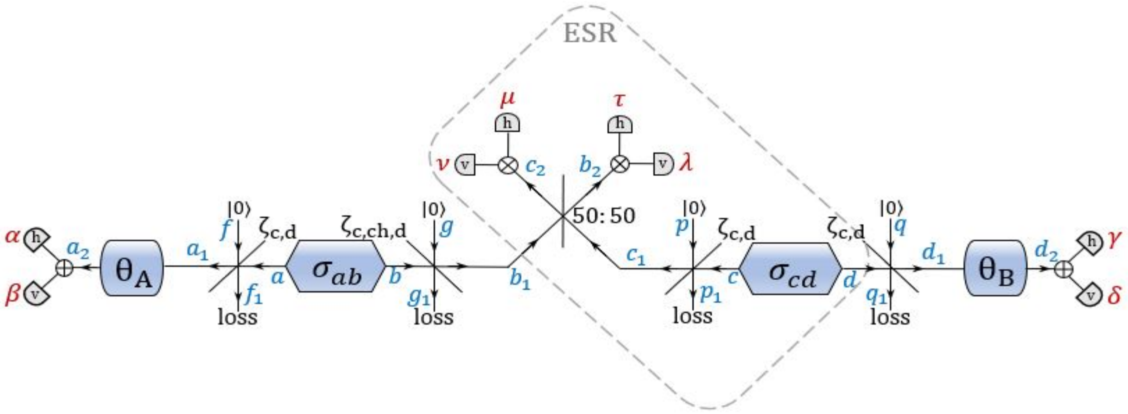

FIG. 1: Schematic of the ESR-based DIQKD setup matching the mathematical models presented in the main text: σ_{ab} and σ_{cd} stand for the entanglement sources, θ_{A} (θ_{B}) denotes the rotation angle of Alice's (Bob's) measurement settings and $\zeta_{\mathrm{c,d}}$ and $\zeta_{\mathrm{c,ch,d}}$ tag the effective efficiency parameters, $\zeta_{\mathrm{c,d}} = \eta_{\mathrm{c}}\eta_{\mathrm{d}}$ and $\zeta_{\mathrm{c,ch,d}} = \eta_{\mathrm{c}}\eta_{\mathrm{ch}}\eta_{\mathrm{d}}$, where η_{c}, η_{d} and η_{ch} denote, respectively, the transmittance of the BSs modeling the coupling loss, the detection inefficiency of the detectors and the channel loss. The symbol "$\oplus$" is used to denote the PBSs that precede the photodetectors. The latin letters in blue color indicate the different modes, while the greek letters in red color are used to tag the number of photons observed at each of the detectors in any given detection event. The output modes f_1, g_1, p_1 and q_1 correspond to the losses, $|0\rangle$ is the vacuum state, and a dashed grey rectangle identifies the ESR.

*Electronic address: vzapatero@com.uvigo.es

Here, we calculate the parameters $P_{\rm SH}$, $Q|_{\rm SH}$ and $\omega|_{\rm SH}$ for an honest implementation of the DIQKD protocol assisted by an ESR at Bob's side. A schematic of the mathematical model that describes the optical setup is given in Fig. 1. This figure includes all the parameters and the relevant notation that shall be used in what follows.

1. *Click pattern distribution*

Since all photodetectors are described with a POVM whose elements are diagonal in the Fock basis, for convenience we will consider that Alice's source emits mixed states of the form

$$\sigma_{ab} = \sum_{n=0}^{\infty} p_n |\phi_n\rangle_{ab}\langle\phi_n|, \tag{3}$$

with

$$|\phi_n\rangle_{ab} = \frac{1}{n!\sqrt{n+1}}(a_{\rm h}^{\dagger}b_{\rm v}^{\dagger} - a_{\rm v}^{\dagger}b_{\rm h}^{\dagger})^n|0\rangle_{ab}, \tag{4}$$

instead of the pure states

$$|\psi\rangle_{ab} = \sum_{n=0}^{\infty} \sqrt{p_n}|\phi_n\rangle_{ab} \tag{5}$$

introduced in the main text (of course, the meaning of $a_{\rm h,v}^{\dagger}$, $b_{\rm h,v}^{\dagger}$ and $|0\rangle_{ab}$ in Eq. (4) is again the one presented there). Note that both σ_{ab} and $|\psi\rangle_{ab}$ deliver exactly the same output statistics when measured in the Fock basis, and thus we can use the state σ_{ab} for the calculations below. Similarly, we will consider that the state emitted by the entanglement source within the qubit amplifier is of the form

$$\sigma_{cd} = \sum_{n=0}^{\infty} p'_n |\phi_n\rangle_{cd}\langle\phi_n|, \tag{6}$$

where the apostrophe in p'_n indicates that the statistics of σ_{cd} are generally different from those of σ_{ab}. For instance, σ_{ab} and σ_{cd} could originate from PDC sources with different intensity parameters.

The starting point for our calculations is then the quantum state

$$\rho_0 = \sigma_{ab}\otimes\sigma_{cd} = \sum_{n=0}^{\infty}\sum_{n'=0}^{\infty} p_n p'_{n'}|\phi_n\rangle_{ab}\langle\phi_n|\otimes|\phi_{n'}\rangle_{cd}\langle\phi_{n'}|. \tag{7}$$

Prior to the interference in the linear optics BSM, the states σ_{ab} and σ_{cd} evolve separately. In particular, let us focus on the evolution of σ_{ab} first. We have that the states $|\phi_n\rangle_{ab}$ can be written as

$$|\phi_n\rangle_{ab} = \frac{1}{n!\sqrt{n+1}}\sum_{i=0}^{n}\binom{n}{i}(-1)^i(a_{\rm v}^{\dagger}b_{\rm h}^{\dagger})^i(a_{\rm h}^{\dagger}b_{\rm v}^{\dagger})^{n-i}|0\rangle. \tag{8}$$

Here and in what follows, we shall simply use $|0\rangle$ to denote the vacuum state in all spatial modes.

We model each photodetector by means of a BS with transmittance $\eta_{\rm d}$ attached to a lossless PNR detector, whose POVM elements are simply given by projectors onto Fock states: $\Pi_j = |j\rangle\langle j|$, $j\in\mathbb{N}$. The effect of dark counts is incorporated a posteriori. Altogether, one can readily check that this model is equivalent to that given in the main text. Moreover, since all the detectors (optical couplers) are assumed to have the same detection (coupling) efficiency $\eta_{\rm d}$ ($\eta_{\rm c}$) one can combine the effect of finite detection and coupling efficiencies on mode a with one BS of transmittance $\zeta_{\rm c,d} = \eta_{\rm c}\eta_{\rm d}$. Further incorporating the channel loss, a BS of transmittance $\zeta_{\rm c,ch,d} = \eta_{\rm c}\eta_{\rm ch}\eta_{\rm d}$ acts on mode b, where $\eta_{\rm ch} = 10^{-\Lambda/10}$ is the transmission efficiency of the fiber link connecting Alice's and Bob's labs (which depends on the channel loss Λ). In doing so, we find that the quantum states $|\phi_n\rangle_{ab}$ evolve to

$$\begin{aligned}|\phi_n\rangle_{a_1b_1;f_1g_1} &= \frac{1}{n!\sqrt{n+1}}\sum_{i=0}^{n}\sum_{j=0}^{i}\sum_{k=0}^{i}\sum_{l=0}^{n-i}\sum_{m=0}^{n-i}(-1)^i\binom{n}{i}\binom{i}{j}\binom{i}{k}\binom{n-i}{l}\binom{n-i}{m}T_{\rm c,d}^{j+l}T_{\rm c,ch,d}^{k+m}R_{\rm c,d}^{n-j-l}R_{\rm c,ch,d}^{n-k-m}\\ &\times\; a_{1,\rm h}^{\dagger\,l}a_{1,\rm v}^{\dagger\,j}b_{1,\rm h}^{\dagger\,k}b_{1,\rm v}^{\dagger\,m}f_{1,\rm h}^{\dagger\,n-i-l}f_{1,\rm v}^{\dagger\,i-j}g_{1,\rm h}^{\dagger\,i-k}g_{1,\rm v}^{\dagger\,n-i-m}|0\rangle,\end{aligned} \tag{9}$$

where the different modes are illustrated in Fig. 1. In Eq. (9) we define $T_{\rm c,d} = {\zeta_{\rm c,d}}^{1/2}$, $T_{\rm c,ch,d} = {\zeta_{\rm c,ch,d}}^{1/2}$, $R_{\rm c,d} = (1-\zeta_{\rm c,d})^{1/2}$ and $R_{\rm c,ch,d} = (1-\zeta_{\rm c,ch,d})^{1/2}$ for readability. Similarly, the state σ_{cd} within the ESR undergoes a similar transformation, so that the pure states $|\phi_{n'}\rangle_{cd}$ evolve to $|\phi_{n'}\rangle_{c_1d_1;p_1q_1}$, whose form is identical to that of $|\phi_n\rangle_{a_1b_1;f_1g_1}$ but taking $T_{\rm c,ch,d} \to T_{\rm c,d}$ and $R_{\rm c,ch,d} \to R_{\rm c,d}$, and now obviously referred to the modes c_1, d_1, p_1 and q_1 (instead of a_1, b_1, f_1 and g_1). Putting it all together, the overall quantum state prior to the interference in the ESR is given by

$$\rho_{\rm BSM} = \sum_{n=0}^{\infty}\sum_{n'=0}^{\infty} p_n p'_{n'} |\phi_n\rangle_{a_1b_1;f_1g_1}\langle\phi_n| \otimes |\phi_{n'}\rangle_{c_1d_1;p_1q_1}\langle\phi_{n'}|, \tag{10}$$

where the pure states $|\phi_n\rangle_{a_1b_1;f_1g_1} \otimes |\phi_{n'}\rangle_{c_1d_1;p_1q_1}$ are written as

$$\begin{aligned} |\phi_n\rangle_{a_1b_1;f_1g_1} \otimes |\phi_{n'}\rangle_{c_1d_1;p_1q_1} &= \frac{1}{n!n'!\sqrt{(n+1)(n'+1)}} \sum_{i=0}^{n}\sum_{x=0}^{n'}\sum_{j=0}^{i}\sum_{y=0}^{x}\sum_{k=0}^{i}\sum_{z=0}^{x}\sum_{l=0}^{n-i}\sum_{t=0}^{n'-x}\sum_{m=0}^{n-i}\sum_{w=0}^{n'-x} (-1)^{i+x}\binom{n}{i}\binom{n'}{x}\binom{i}{j} \\ &\times \binom{x}{y}\binom{i}{k}\binom{x}{z}\binom{n-i}{l}\binom{n'-x}{t}\binom{n-i}{m}\binom{n'-x}{w} T_{\rm c,d}^{j+l+y+z+t+w} T_{\rm c,ch,d}^{k+m} R_{\rm c,d}^{n+2n'-j-l-y-z-t-w} R_{\rm c,ch,d}^{n-k-m} {a_{1,\rm h}^\dagger}^{l} {a_{1,\rm v}^\dagger}^{j} \\ &\times {b_{1,\rm h}^\dagger}^{k} {b_{1,\rm v}^\dagger}^{m} {c_{1,\rm h}^\dagger}^{t} {c_{1,\rm v}^\dagger}^{y} {d_{1,\rm h}^\dagger}^{z} {d_{1,\rm v}^\dagger}^{w} {f_{1,\rm h}^\dagger}^{n-i-l} {f_{1,\rm v}^\dagger}^{i-j} {g_{1,\rm h}^\dagger}^{i-k} {g_{1,\rm v}^\dagger}^{n-i-m} {p_{1,\rm h}^\dagger}^{n'-x-t} {p_{1,\rm v}^\dagger}^{x-y} {q_{1,\rm h}^\dagger}^{x-z} {q_{1,\rm v}^\dagger}^{n'-x-w} |0\rangle. \end{aligned} \tag{11}$$

Next, modes b_1 and c_1 interfere at a 50:50 BS within the ESR. The corresponding transformation is given by

$${b_{1,\rm h}^\dagger}^{k} {b_{1,\rm v}^\dagger}^{m} {c_{1,\rm h}^\dagger}^{t} {c_{1,\rm v}^\dagger}^{y} \xrightarrow{50:50} \sum_{h=0}^{k}\sum_{r=0}^{m}\sum_{u=0}^{t}\sum_{v=0}^{y} \frac{(-1)^{u+v}}{\sqrt{2}^{k+m+t+y}} \binom{k}{h}\binom{m}{r}\binom{t}{u}\binom{y}{v} {b_{2,\rm h}^\dagger}^{h+u} {b_{2,\rm v}^\dagger}^{r+v} {c_{2,\rm h}^\dagger}^{k+t-h-u} \times {c_{2,\rm v}^\dagger}^{m+y-r-v}. \tag{12}$$

If we define the sum variables $s = h + u$ and $o = r + v$, and we use the fact that a rectangle $\{0 \le a \le A, 0 \le b \le B\}$ can be equivalently characterised by $\{0 \le s \le A + B, \max(0, s - A) \le b \le \min(s, B)\}$, we have that the RHS of Eq. (12) can be written as

$$\sum_{s=0}^{k+t}\sum_{o=0}^{m+y}\sum_{u=\max\{0,s-k\}}^{\min\{s,t\}}\sum_{v=\max\{0,o-m\}}^{\min\{o,y\}} \frac{(-1)^{u+v}}{\sqrt{2}^{k+m+t+y}} \binom{k}{s-u}\binom{m}{o-v}\binom{t}{u}\binom{y}{v} {b_{2,\rm h}^\dagger}^{s} {b_{2,\rm v}^\dagger}^{o} {c_{2,\rm h}^\dagger}^{k+t-s} {c_{2,\rm v}^\dagger}^{m+y-o}. \tag{13}$$

This means that the state $|\phi_n\rangle_{a_1b_1;f_1g_1} \otimes |\phi_{n'}\rangle_{c_1d_1;p_1q_1}$ is transformed into $|\phi_{n,n'}\rangle_{a_1b_2c_2d_1;f_1g_1p_1q_1}$ given by

$$\begin{aligned} |\phi_{n,n'}\rangle_{a_1b_2c_2d_1;f_1g_1p_1q_1} &= \frac{1}{n!n'!\sqrt{(n+1)(n'+1)}} \sum_{i=0}^{n}\sum_{x=0}^{n'}\sum_{j=0}^{i}\sum_{y=0}^{x}\sum_{k=0}^{i}\sum_{z=0}^{x}\sum_{l=0}^{n-i}\sum_{t=0}^{n'-x}\sum_{m=0}^{n-i}\sum_{w=0}^{n'-x}\sum_{s=0}^{k+t}\sum_{o=0}^{m+y}\sum_{u=\max\{0,s-k\}}^{\min\{s,t\}} \\ &\times \sum_{v=\max\{0,o-m\}}^{\min\{o,y\}} \frac{(-1)^{i+x+u+v}}{\sqrt{2}^{k+m+t+y}} \binom{n}{i}\binom{n'}{x}\binom{i}{j}\binom{x}{y}\binom{i}{k}\binom{x}{z}\binom{n-i}{l}\binom{n'-x}{t}\binom{n-i}{m}\binom{n'-x}{w}\binom{k}{s-u}\binom{m}{o-v}\binom{t}{u} \\ &\times \binom{y}{v} T_{\rm c,d}^{j+l+y+z+t+w} T_{\rm c,ch,d}^{k+m} R_{\rm c,d}^{n+2n'-j-l-y-z-t-w} R_{\rm c,ch,d}^{n-k-m} {a_{1,\rm h}^\dagger}^{l} {a_{1,\rm v}^\dagger}^{j} {d_{1,\rm h}^\dagger}^{z} {d_{1,\rm v}^\dagger}^{w} {b_{2,\rm h}^\dagger}^{s} {b_{2,\rm v}^\dagger}^{o} {c_{2,\rm h}^\dagger}^{k+t-s} {c_{2,\rm v}^\dagger}^{m+y-o} \\ &\times {f_{1,\rm h}^\dagger}^{n-i-l} {f_{1,\rm v}^\dagger}^{i-j} {g_{1,\rm h}^\dagger}^{i-k} {g_{1,\rm v}^\dagger}^{n-i-m} {p_{1,\rm h}^\dagger}^{n'-x-t} {p_{1,\rm v}^\dagger}^{x-y} {q_{1,\rm h}^\dagger}^{x-z} {q_{1,\rm v}^\dagger}^{n'-x-w} |0\rangle. \end{aligned} \tag{14}$$

Now, we incorporate Alice's and Bob's measurement settings by transforming the operators of the affected modes, a_1 and d_1, with rotation angles $\theta_{\rm A}$ and $\theta_{\rm B}$, respectively. We denote the corresponding output pure state by

$|\phi_{n,n'}\rangle^{\theta_{\rm A},\theta_{\rm B}}_{a_2b_2c_2d_2;f_1g_1p_1q_1}$, and it is given by

$$|\phi_{n,n'}\rangle^{\theta_{\rm A},\theta_{\rm B}}_{a_2b_2c_2d_2;f_1g_1p_1q_1} = \frac{1}{n!n'!\sqrt{(n+1)(n'+1)}}\sum_{i=0}^{n}\sum_{x=0}^{n'}\sum_{j=0}^{i}\sum_{y=0}^{x}\sum_{k=0}^{i}\sum_{z=0}^{x}\sum_{l=0}^{n-i}\sum_{t=0}^{n'-x}\sum_{m=0}^{n-i}\sum_{w=0}^{n'-x}\sum_{s=0}^{k+t}\sum_{o=0}^{m+y}\sum_{u=\max\{0,s-k\}}^{\min\{s,t\}}$$
$$\times \sum_{v=\max\{0,o-m\}}^{\min\{o,y\}}\sum_{e=0}^{l+j}\sum_{r=0}^{z+w}\sum_{h=\max\{0,e-l\}}^{\min\{e,j\}}\sum_{\tilde{n}=\max\{0,r-z\}}^{\min\{r,w\}}\binom{n}{i}\binom{n'}{x}\binom{i}{j}\binom{x}{y}\binom{i}{k}\binom{x}{z}\binom{n-i}{l}\binom{n'-x}{t}\binom{n-i}{m}\binom{n'-x}{w}$$
$$\times \binom{k}{s-u}\binom{m}{o-v}\binom{t}{u}\binom{y}{v}\binom{l}{e-h}\binom{j}{h}\binom{z}{r-\tilde{n}}\binom{w}{\tilde{n}}\frac{(-1)^{i+x+u+v+h+\tilde{n}}}{\sqrt{2}^{k+m+t+y}}T_{\rm c,d}^{j+l+y+z+t+w}T_{\rm c,ch,d}^{k+m}R_{\rm c,d}^{n+2n'-j-l-y-z-t-w}$$
$$\times R_{\rm c,ch,d}^{n-k-m}\cos\theta_{\rm A}{}^{e+j-2h}\sin\theta_{\rm A}{}^{l+2h-e}\cos\theta_{\rm B}{}^{r+w-2\tilde{n}}\sin\theta_{\rm B}{}^{z+2\tilde{n}-r}a_{2,\rm h}^{\dagger}{}^{e}a_{2,\rm v}^{\dagger}{}^{l+j-e}d_{2,\rm h}^{\dagger}{}^{r}d_{2,\rm v}^{\dagger}{}^{z+w-r}b_{2,\rm h}^{\dagger}{}^{s}b_{2,\rm v}^{\dagger}{}^{o}c_{2,\rm h}^{\dagger}{}^{k+t-s}$$
$$\times c_{2,\rm v}^{\dagger}{}^{m+y-o}f_{1,\rm h}^{\dagger}{}^{n-i-l}f_{1,\rm v}^{\dagger}{}^{i-j}g_{1,\rm h}^{\dagger}{}^{i-k}g_{1,\rm v}^{\dagger}{}^{n-i-m}p_{1,\rm h}^{\dagger}{}^{n'-x-t}p_{1,\rm v}^{\dagger}{}^{x-y}q_{1,\rm h}^{\dagger}{}^{x-z}q_{1,\rm v}^{\dagger}{}^{n'-x-w}|0\rangle. \qquad (15)$$

That is, Eq. (15) describes the quantum state immediately prior to the detectors, conditioned on the photon pair numbers n and n'.

Importantly, as the detection efficiencies of all detectors were already accounted for in the effective BS models, projective measurements onto Fock states must be considered now, as explained above. More precisely, we are interested in the conditional probability, $\mathrm{P}\left(\vec{\alpha}|n,n'\right)_{\theta_{\rm A},\theta_{\rm B}}$, of observing a detection pattern $\vec{\alpha}$ given the quantum state $|\phi_{n,n'}\rangle^{\theta_{\rm A},\theta_{\rm B}}_{a_2b_2c_2d_2;f_1g_1p_1q_1}$. Here, we introduced the vector notation $\vec{\alpha}=(\alpha,\beta,\gamma,\delta,\mu,\nu,\tau,\lambda)$, where each element of the vector denotes the number of photons in a certain output mode: α refers to mode $a_{2,\rm h}$, β to mode $a_{2,\rm v}$, γ to mode $d_{2,\rm h}$, δ to mode $d_{2,\rm v}$, μ to mode $c_{2,\rm h}$, ν to mode $c_{2,\rm v}$, τ to mode $b_{2,\rm h}$ and λ to mode $b_{2,\rm v}$. With this notation, and if we disregard for the moment the effect of dark counts, we have that $\mathrm{P}\left(\vec{\alpha}|n,n'\right)_{\theta_{\rm A},\theta_{\rm B}}$ is given by

$$\mathrm{P}\left(\vec{\alpha}|n,n'\right)_{\theta_{\rm A},\theta_{\rm B}} = \left\||\tilde{\phi}_{n,n'}\rangle^{\theta_{\rm A},\theta_{\rm B}}_{\vec{\alpha};f_1g_1p_1q_1}\right\|^2, \qquad (16)$$

where the unnormalised state $|\tilde{\phi}_{n,n'}\rangle^{\theta_{\rm A},\theta_{\rm B}}_{\vec{\alpha};f_1g_1p_1q_1}$ has the form

$$|\tilde{\phi}_{n,n'}\rangle^{\theta_{\rm A},\theta_{\rm B}}_{\vec{\alpha};f_1g_1p_1q_1} = \langle\vec{\alpha}|\phi_{n,n'}\rangle^{\theta_{\rm A},\theta_{\rm B}}_{a_2b_2c_2d_2;f_1g_1p_1q_1}, \qquad (17)$$

being $|\vec{\alpha}\rangle=|\alpha,\beta,\gamma,\delta,\mu,\nu,\tau,\lambda\rangle$. To compute $|\tilde{\phi}_{n,n'}\rangle^{\theta_{\rm A},\theta_{\rm B}}_{\vec{\alpha};f_1g_1p_1q_1}$, we make use of the following orthogonality relation:

$$\langle\vec{\alpha}|a_{2,\rm h}^{\dagger}{}^{e}a_{2,\rm v}^{\dagger}{}^{l+j-e}d_{2,\rm h}^{\dagger}{}^{r}d_{2,\rm v}^{\dagger}{}^{z+w-r}c_{2,\rm h}^{\dagger}{}^{k+t-s}c_{2,\rm v}^{\dagger}{}^{m+y-o}b_{2,\rm h}^{\dagger}{}^{s}b_{2,\rm v}^{\dagger}{}^{o}|0\rangle =$$
$$(\alpha!\beta!\gamma!\delta!\mu!\nu!\tau!\lambda!)^{1/2}\,\delta_{\alpha}^{e}\delta_{\beta}^{l+j-e}\delta_{\gamma}^{r}\delta_{\delta}^{z+w-r}\times\delta_{\mu}^{k+t-s}\delta_{\nu}^{m+y-o}\delta_{\tau}^{s}\delta_{\lambda}^{o}, \qquad (18)$$

where δ_i^j stands for the Kronecker's delta symbol, *i.e.*, $\delta_i^j=1$ only if $i=j$, otherwise it is zero. Also, we recall that for finite range sums, $\sum_{a=A_1}^{A_2}f(a)\delta_x^a=f(x)\Theta_{x-A_1}\Theta_{A_2-x}$, where Θ_j is the "discrete" Heaviside function [2]. Then, in order to incorporate the effect of the different Θ's, one must modify the affected index ranges accordingly. This yields

$$|\tilde{\phi}_{n,n'}\rangle^{\theta_{\rm A},\theta_{\rm B}}_{\vec{\alpha};f_1g_1p_1q_1} = \frac{1}{n!n'!}\left[\frac{\alpha!\beta!\gamma!\delta!\mu!\nu!\tau!\lambda!}{(n+1)(n'+1)2^{\mu+\nu+\tau+\lambda}}\right]^{\frac{1}{2}}T_{\rm c,d}^{\alpha+\beta+\gamma+\delta+\mu+\tau}T_{\rm c,ch,d}^{\nu+\lambda}R_{\rm c,d}^{n+2n'-\alpha-\beta-\gamma-\delta-\mu-\tau}R_{\rm c,ch,d}^{n-\nu-\lambda}\cos\theta_{\rm A}{}^{\alpha}$$
$$\times\sin\theta_{\rm A}{}^{\beta}\cos\theta_{\rm B}{}^{\gamma}\sin\theta_{\rm B}{}^{\delta}\sum_{i=0}^{n}\sum_{x=0}^{n'}\sum_{j=\max\{0,\alpha+\beta+i-n\}}^{\min\{i,\alpha+\beta\}}\sum_{y=\max\{0,\nu+\lambda+i-n\}}^{\min\{x,\nu+\lambda\}}\sum_{k=\max\{0,\mu+\tau+x-n'\}}^{\min\{i,\mu+\tau\}}\sum_{w=\max\{0,\gamma+\delta-x\}}^{\min\{n'-x,\gamma+\delta\}}\sum_{u=\max\{0,\tau-k\}}^{\min\{\tau,\mu+\tau-k\}}$$
$$\times\sum_{v=\max\{0,y-\nu\}}^{\min\{\lambda,y\}}\sum_{h=\max\{0,j-\beta\}}^{\min\{\alpha,j\}}\sum_{\tilde{n}=\max\{0,w-\delta\}}^{\min\{\gamma,w\}}\binom{n}{i}\binom{n'}{x}\binom{i}{j}\binom{x}{y}\binom{i}{k}\binom{x}{\gamma+\delta-w}\binom{n-i}{\alpha+\beta-j}\binom{n'-x}{\mu+\tau-k}\binom{w}{\tilde{n}}$$
$$\times\binom{n-i}{\nu+\lambda-y}\binom{n'-x}{w}\binom{k}{\tau-u}\binom{\nu+\lambda-y}{\lambda-v}\binom{\mu+\tau-k}{u}\binom{y}{v}\binom{\alpha+\beta-j}{\alpha-h}\binom{j}{h}\binom{\gamma+\delta-w}{\gamma-\tilde{n}}\left(\frac{T_{\rm c,d}R_{\rm c,ch,d}}{T_{\rm c,ch,d}R_{\rm c,d}}\right)^{y-k}$$
$$\times(-1)^{i+x+u+v+h+\tilde{n}}\cos\theta_{\rm A}{}^{j-2h}\sin\theta_{\rm A}{}^{2h-j}\cos\theta_{\rm B}{}^{w-2\tilde{n}}\sin\theta_{\rm B}{}^{2\tilde{n}-w}f_{1,\rm h}^{\dagger}{}^{n+j-i-\alpha-\beta}f_{1,\rm v}^{\dagger}{}^{i-j}g_{1,\rm h}^{\dagger}{}^{i-k}g_{1,\rm v}^{\dagger}{}^{n+y-i-\nu-\lambda}$$
$$\times p_{1,\rm h}^{\dagger}{}^{n'+k-x-\mu-\tau}p_{1,\rm v}^{\dagger}{}^{x-y}q_{1,\rm h}^{\dagger}{}^{x+w-\gamma-\delta}q_{1,\rm v}^{\dagger}{}^{n'-x-w}|0\rangle, \qquad (19)$$

where some overall constant terms were factored from the sums. Then, by applying Eq. (16) on the state given by Eq. (19), we obtain

$$\mathrm{P}\left(\vec{\alpha}|n,n'\right)_{\theta_{\rm A},\theta_{\rm B}} = \frac{\alpha!\beta!\gamma!\delta!\mu!\nu!\tau!\lambda!\eta_{\rm B}^{\nu+\lambda}}{(n+1)(n'+1)2^{\mu+\nu+\tau+\lambda}}\eta_{\rm A}^{\alpha+\beta+\gamma+\delta+\mu+\tau}(1-\zeta_{\rm c,d})^{n+2n'-\alpha-\beta-\gamma-\delta-\mu-\tau}(1-\zeta_{\rm c,ch,d})^{n-\nu-\lambda}\cos\theta_{\rm A}{}^{2\alpha}$$
$$\times\sin\theta_{\rm A}{}^{2\beta}\cos\theta_{\rm B}{}^{2\gamma}\sin\theta_{\rm B}{}^{2\delta}\sum_{i=0}^{n}\sum_{\Delta=-i}^{n-i}\sum_{x=\max\{0,-\Delta\}}^{\min\{n',n'-\Delta\}}\sum_{j=\max\{0,\alpha+\beta+i-n,-\Delta\}}^{\min\{i,\alpha+\beta,\alpha+\beta-\Delta\}}\sum_{y=\max\{0,\nu+\lambda+i-n,-\Delta\}}^{\min\{x,\nu+\lambda,\nu+\lambda-\Delta\}}\sum_{k=\max\{0,\mu+\tau+x-n',-\Delta\}}^{\min\{i,\mu+\tau,\mu+\tau-\Delta\}}$$
$$\times\sum_{w=\max\{0,\gamma+\delta-x,\Delta\}}^{\min\{n'-x,\gamma+\delta,\gamma+\delta+\Delta\}}\sum_{u=\max\{0,\tau-k\}}^{\min\{\tau,\mu+\tau-k\}}\sum_{U=\max\{0,\tau-k-\Delta\}}^{\min\{\tau,\mu+\tau-k-\Delta\}}\sum_{v=\max\{0,y-\nu\}}^{\min\{\lambda,y\}}\sum_{V=\max\{0,y+\Delta-\nu\}}^{\min\{\lambda,y+\Delta\}}\sum_{h=\max\{0,j-\beta\}}^{\min\{\alpha,j\}}\sum_{H=\max\{0,j+\Delta-\beta\}}^{\min\{\alpha,j+\Delta\}}$$
$$\times\sum_{\tilde{n}=\max\{0,w-\delta\}}^{\min\{\gamma,w\}}\sum_{\tilde{N}=\max\{0,w-\Delta-\delta\}}^{\min\{\gamma,w-\Delta\}}\left[\frac{\zeta_{\rm c,d}(1-\zeta_{\rm c,ch,d})}{\zeta_{\rm c,ch,d}(1-\zeta_{\rm c,d})}\right]^{y-k}(-1)^{u+v+h+\tilde{n}+U+V+H+\tilde{N}}\left(\frac{\sin\theta_{\rm A}}{\cos\theta_{\rm A}}\right)^{2(h+H-j)-\Delta}$$
$$\times\left(\frac{\sin\theta_{\rm B}}{\cos\theta_{\rm B}}\right)^{2(\tilde{n}+\tilde{N}-w)+\Delta}\Upsilon(n,n',i,x,j,y,k,w,u,U,v,V,h,H,\tilde{n},\tilde{N},\Delta,\alpha,\beta,\gamma,\delta,\mu,\nu,\tau,\lambda), \tag{20}$$

where we have defined

$$\Upsilon(n,n',i,x,j,y,k,w,u,U,v,V,h,H,\tilde{n},\tilde{N},\Delta,\alpha,\beta,\gamma,\delta,\mu,\nu,\tau,\lambda) =$$
$$\times\, i!(i+\Delta)!x!(x+\Delta)!(n-i)!(n-i-\Delta)!(n'-x)!(n'-x-\Delta)!(i-j)!(x-y)!(i-k)!$$
$$\times\frac{[(x+w-\gamma-\delta)!(n+j-i-\alpha-\beta)!(n'+k-x-\mu-\tau)!(n+y-i-\nu-\lambda)!(n'-x-w)!]^{-1}}{h!H!(j-h)!(j+\Delta-H)!v!V!(y-v)!(y+\Delta-V)!(\tau-u)!(\tau-U)!(k+u-\tau)!(k+\Delta+U-\tau)!}$$
$$\times\frac{[(\gamma-\tilde{n})!(\gamma-\tilde{N})!(\delta+\tilde{n}-w)!(\delta+\tilde{N}+\Delta-w)!(\alpha-h)!(\alpha-H)!(\beta+h-j)!(\beta+H-j-\Delta)!u!U!]^{-1}}{(\mu+\tau-k-u)!(\mu+\tau-k-\Delta-U)!(\lambda-v)!(\lambda-V)!(\nu+v-y)!(\nu+V-y-\Delta)!\tilde{n}!\tilde{N}!(w-\tilde{n})!(w-\Delta-\tilde{N})!}. \tag{21}$$

Of course, the condition $\sum_{\vec{\alpha}}\mathrm{P}\left(\vec{\alpha}|n,n'\right)_{\theta_{\rm A},\theta_{\rm B}} = 1$ holds, and only those click patterns $\vec{\alpha}$ such that $\alpha+\beta\leq n$, $\gamma+\delta\leq n'$ and $\mu+\nu+\tau+\lambda\leq n+n'$ give a non vanishing contribution due to the absence of noise, which we take into account next.

In particular, if one considers the noise model introduced in the main text, we find that the resulting distribution in the noisy scenario is given by

$$\tilde{\mathrm{P}}\left(\vec{\alpha}|n,n'\right)_{\theta_{\rm A},\theta_{\rm B}} = (1-8p_{\rm d})\mathrm{P}\left(\vec{\alpha}|n,n'\right)_{\theta_{\rm A},\theta_{\rm B}} + p_{\rm d}\sum_{\vec{\sigma}\in\Gamma_{\vec{\alpha}}}\mathrm{P}\left(\vec{\sigma}|n,n'\right)_{\theta_{\rm A},\theta_{\rm B}} + O(p_{\rm d}^2), \tag{22}$$

where $\Gamma_{\vec{\alpha}} = \{\vec{\sigma} : |\vec{\alpha}| = |\vec{\sigma}|+1\}$, $|\vec{\alpha}|$ being the overall number of photons corresponding to the pattern $\vec{\alpha}$, *i.e.*, $|\vec{\alpha}| = \alpha+\beta+\gamma+\delta+\mu+\nu+\tau+\lambda$. As a consequence, and up to first order in $p_{\rm d}$, we have that $\tilde{\mathrm{P}}\left(\vec{\alpha}|n,n'\right)_{\theta_{\rm A},\theta_{\rm B}}$ vanishes for any pattern $\vec{\alpha}$ that does not fulfill $\alpha+\beta\leq n+1$, $\gamma+\delta\leq n'+1$, $\mu+\nu+\tau+\lambda\leq n+n'+1$ and $\alpha+\beta+\gamma+\delta+\mu+\nu+\tau+\lambda\leq 2(n+n')+1$.

Alice's and Bob's outcomes are expected to be anti-correlated, as the entanglement sources we are considering emit singlet states. Therefore, we flip say Alice's outcomes in such a way that, whenever Alice and Bob select the same measurement settings, their outcomes are correlated. For this, we define the distribution

$$\tilde{\mathrm{p}}\left(\vec{\alpha}|n,n'\right)_{\theta_{\rm A},\theta_{\rm B}} = \tilde{\mathrm{P}}_{\alpha\leftrightarrow\beta}\left(\vec{\alpha}|n,n'\right)_{\theta_{\rm A},\theta_{\rm B}}. \tag{23}$$

That is to say, $\tilde{\mathrm{p}}\left(\alpha,\beta,\gamma,\delta,\mu,\nu,\tau,\lambda|n,n'\right)_{\theta_{\rm A},\theta_{\rm B}} = \tilde{\mathrm{P}}\left(\beta,\alpha,\gamma,\delta,\mu,\nu,\tau,\lambda|n,n'\right)_{\theta_{\rm A},\theta_{\rm B}}$.

Finally, it only remains to take into account the deterministic assignment performed by Alice and Bob whenever they observe an inconclusive event, required to have a distribution with binary outcomes on both sides. To be precise, the assignments read

$$A_{\rm A} = \begin{cases} 0 & \text{if } (\alpha,\beta)=(1,0) \\ 1 & \text{otherwise,}\end{cases}\quad A_{\rm B} = \begin{cases} 0 & \text{if } (\gamma,\delta)=(1,0) \\ 1 & \text{otherwise.}\end{cases} \tag{24}$$

In this way, every possible event $(\alpha,\beta,\gamma,\delta)$ regarding Alice's and Bob's detector outcomes is mapped to an element of the set of binary strings $\{(0,0),(0,1),(1,0),(1,1)\}$. Importantly, we remark that such a post-processing is not performed on the outcomes of the photodetectors inside the qubit amplifier, but only on the outcomes observed by the parties. In summary, the distribution we are finally interested in is that of the "post-processed click pattern" $A_{\vec{\alpha}}=(A_{\rm A},A_{\rm B},\mu,\nu,\tau,\lambda)$, which we shall denote by $\mathbf{P}(A_{\vec{\alpha}}|n,n')_{\theta_{\rm A},\theta_{\rm B}}$. From Eqs. 24, it is obvious that

$$\begin{aligned}
\mathbf{P}(0,0,\mu,\nu,\tau,\lambda|n,n')_{\theta_{\rm A},\theta_{\rm B}} &= \tilde{\rm p}\left(1,0,1,0,\mu,\nu,\tau,\lambda|n,n'\right)_{\theta_{\rm A},\theta_{\rm B}},\\
\mathbf{P}(0,1,\mu,\nu,\tau,\lambda|n,n')_{\theta_{\rm A},\theta_{\rm B}} &= \sum_{(\gamma,\delta)\neq(1,0)} \tilde{\rm p}\left(1,0,\gamma,\delta,\mu,\nu,\tau,\lambda|n,n'\right)_{\theta_{\rm A},\theta_{\rm B}},\\
\mathbf{P}(1,0,\mu,\nu,\tau,\lambda|n,n')_{\theta_{\rm A},\theta_{\rm B}} &= \sum_{(\alpha,\beta)\neq(1,0)} \tilde{\rm p}\left(\alpha,\beta,1,0,\mu,\nu,\tau,\lambda|n,n'\right)_{\theta_{\rm A},\theta_{\rm B}},\\
\mathbf{P}(1,1,\mu,\nu,\tau,\lambda|n,n')_{\theta_{\rm A},\theta_{\rm B}} &= \sum_{(\alpha,\beta)\neq(1,0)}\sum_{(\gamma,\delta)\neq(1,0)} \tilde{\rm p}\left(\alpha,\beta,\gamma,\delta,\mu,\nu,\tau,\lambda|n,n'\right)_{\theta_{\rm A},\theta_{\rm B}}.
\end{aligned} \tag{25}$$

2. *Parameters $P_{\rm SH}$, $Q|_{\rm SH}$ and $\omega|_{\rm SH}$*

To obtain the value of these parameters we have to take into account that there are four different click patterns in the ESR which are considered to be successful heralding events, as explained in the main text. These are $(\mu,\nu,\tau,\lambda)=\{(1,1,0,0),(0,1,1,0),(1,0,0,1),(0,0,1,1)\}$. Due to the symmetries of the channel model, we can consider only one of these successful heralding events, say $\Omega=\{(\mu,\nu,\tau,\lambda)=(1,1,0,0)\}$, and the next holds: $P_{\rm SH}=4P_\Omega$, $\omega|_{\rm SH}=\omega|_\Omega$ and $Q|_{\rm SH}=Q|_\Omega$. As a consequence, we can restrict ourselves to the calculation of P_Ω, $Q|_\Omega$ and $\omega|_\Omega$.

To begin with, we have that the probability P_Ω is simply given by

$$P_\Omega=\sum_{A_{\rm A},A_{\rm B}}\mathbf{P}(A_{\rm A},A_{\rm B},\Omega)_{\theta_{\rm A},\theta_{\rm B}}=\sum_{n,n'}p_n p'_{n'}\sum_{A_{\rm A},A_{\rm B}}\mathbf{P}(A_{\rm A},A_{\rm B},\Omega|n,n')_{\theta_{\rm A},\theta_{\rm B}}, \tag{26}$$

where $A_{\rm A},A_{\rm B}\in\{0,1\}$. Obviously, since we are summing over all possible measurement outcomes for Alice and Bob in Eq. (26), P_Ω does not depend on the rotation angles $\theta_{\rm A}$ and $\theta_{\rm B}$. Therefore, one can simply set them both to zero for the numerical calculations.

Secondly, the conditional QBER is given by

$$Q|_\Omega=\frac{1}{P_\Omega}\left[\mathbf{P}(0,1,\Omega)_{0,0}+\mathbf{P}(1,0,\Omega)_{0,0}\right]=\frac{1}{P_\Omega}\sum_{n,n'}p_n p'_{n'}\left[\mathbf{P}(0,1,\Omega|n,n')_{0,0}+\mathbf{P}(1,0,\Omega|n,n')_{0,0}\right]. \tag{27}$$

We remark that this quantity is referred to the events in which both parties select the Z-basis, so that $\theta_{\rm A}=\theta_{\rm B}=0$.

Finally, the conditional winning probability at the CHSH game, $\omega|_\Omega$, can be defined in terms of the conditional CHSH violation, $S|_\Omega$, via

$$\omega|_\Omega=\frac{1}{8}S|_\Omega+\frac{1}{2}, \tag{28}$$

where [3]

$$S|_\Omega=E_{0,-\frac{\pi}{8}}|_\Omega+E_{0,\frac{\pi}{8}}|_\Omega+E_{\frac{\pi}{4},-\frac{\pi}{8}}|_\Omega-E_{\frac{\pi}{4},\frac{\pi}{8}}|_\Omega, \tag{29}$$

and the parameters $E_{\theta_{\rm A},\theta_{\rm B}}|_\Omega$ are given by

$$\begin{aligned}
E_{\theta_{\rm A},\theta_{\rm B}}|_\Omega &= \frac{1}{P_\Omega}\left[\mathbf{P}(1,1,\Omega)_{\theta_{\rm A},\theta_{\rm B}}+\mathbf{P}(0,0,\Omega)_{\theta_{\rm A},\theta_{\rm B}}\right]-\frac{1}{P_\Omega}\left[\mathbf{P}(0,1,\Omega)_{\theta_{\rm A},\theta_{\rm B}}+\mathbf{P}(1,0,\Omega)_{\theta_{\rm A},\theta_{\rm B}}\right]\\
&= \frac{2}{P_\Omega}\sum_{n,n'}p_n p'_{n'}[\mathbf{P}(0,0,\Omega|n,n')_{\theta_{\rm A},\theta_{\rm B}}+\mathbf{P}(1,1,\Omega|n,n')_{\theta_{\rm A},\theta_{\rm B}}]-1.
\end{aligned} \tag{30}$$

We note that in the second equality of Eq. (30), we simply used Eq. (26) and the law of total probability, conditioning on the photon numbers n and n'.

B. Polarizing qubit amplifier

In this Appendix we now consider a PQA-based implementation of the DIQKD protocol. A schematic of the mathematical model that describes the optical setup is illustrated in Fig. 2.

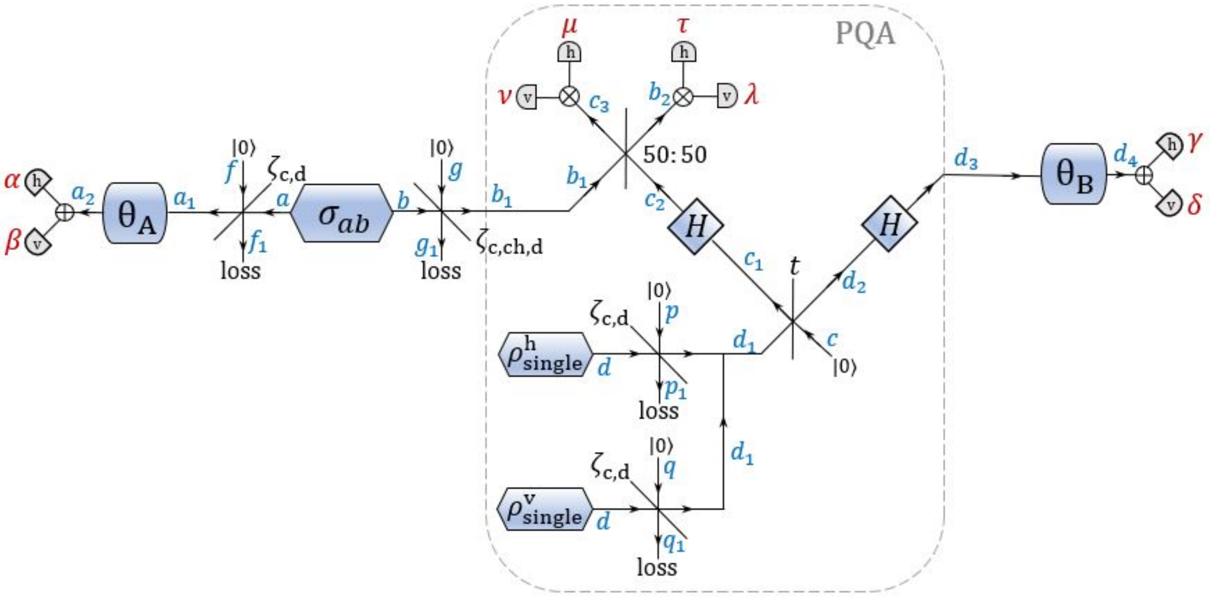

FIG. 2: Schematic of the PQA-based DIQKD setup matching the mathematical models presented in the main text. σ_{ab} stands for the entanglement source held by Alice, while $\rho^{\rm h}_{\rm single}$ and $\rho^{\rm v}_{\rm single}$ denote the single-photon sources inside the PQA. $\theta_{\rm A}$ ($\theta_{\rm B}$) denotes the rotation angle of Alice's (Bob's) measurement settings and $\zeta_{\rm c,d}$ and $\zeta_{\rm c,ch,d}$ tag the effective efficiency parameters, $\zeta_{\rm c,d} = \eta_{\rm c}\eta_{\rm d}$ and $\zeta_{\rm c,ch,d} = \eta_{\rm c}\eta_{\rm ch}\eta_{\rm d}$, where $\eta_{\rm c}$, $\eta_{\rm d}$ and $\eta_{\rm ch}$ denote, respectively, the transmittance of the BSs modeling the coupling loss, the detection inefficiency and the channel loss. Again, the symbol "$\oplus$" is used to denote the PBSs that precede the photodetectors. Similarly, the latin letters in blue color indicate the different modes, while the greek letters in red color are used to tag the number of photons observed at each of the detectors. The output modes f_1, g_1, p_1 and q_1 correspond to the losses, $|0\rangle$ is the vacuum state, t is the tunable transmittance of the BS within the PQA, two Hadamard gates are denoted by H, and a dashed grey rectangle identifies the PQA.

In the main text, only the scenario with ideal single-photon sources $\rho^{\rm h}_{\rm single}$ and $\rho^{\rm v}_{\rm single}$ is considered. Here, however, we assume a more general type of single-photon sources that are built up using a photon pairs source. Precisely, one of the modes ("idler mode") is attached to a photodetector, and it is employed to herald the presence of a single-photon signal in the other mode ("signal mode"). For this reason, we refer to this kind of sources as triggered single-photon sources, a special case of which are of course the ideal sources described in the main text. Note that, in contrast to the ESR-based implementation, Alice and Bob only keep those detection events for which, first, each triggered single-photon source at Bob's lab records one photon in its idler mode, and second, a successful BSM occurs. This being the case, we need to calculate the trigger probability $P_{\rm trigger}$ at the idler mode of a source of this kind, and the conditional quantum state $\rho_{\rm single}$ at its signal mode, given that a trigger occurred. This is what we do next.

1. *Triggered single-photon sources*

We shall consider triggered single-photon sources generated by measuring the idler mode of an entangled state of the form $|\Psi\rangle = \sum_{n=0}^{\infty} \sqrt{p_n}|n,n\rangle$ with a PNR detector. We have that $P_{\rm trigger}$ can be written as

$$P_{\rm trigger} = (1 - p_{\rm d})P_{\rm trigger|0d} + p_{\rm d}P_{\rm trigger|1d}, \tag{31}$$

where $P_{\text{trigger}|0\text{d}}$ ($P_{\text{trigger}|1\text{d}}$) denotes de trigger probability given that there is no dark count (one single dark count) in the detector. These conditional probabilities can be written as

$$P_{\text{trigger}|0\text{d}} = \sum_{n=0}^{\infty} p_n P_{\text{trigger}|n,0\text{d}}, \qquad P_{\text{trigger}|1\text{d}} = \sum_{n=0}^{\infty} p_n P_{\text{trigger}|n,1\text{d}}. \tag{32}$$

where $P_{\text{trigger}|n,0\text{d}} = n\zeta_{\text{c,d}}(1-\zeta_{\text{c,d}})^{n-1}$ and $P_{\text{trigger}|n,1\text{d}} = (1-\zeta_{\text{c,d}})^n$. Here, like in App. I A, $\zeta_{\text{c,d}} = \eta_\text{c}\eta_\text{d}$ with η_c and η_d being, respectively, the coupling and the detection efficiencies.

Similarly, the conditional quantum state at the signal mode given that a trigger occurred has the form $\rho_{\text{single}} = \sum_{n=0}^{\infty} r_n |n\rangle\langle n|$, where the probability distribution r_n can be written as:

$$r_n = \frac{p_n[(1-p_\text{d})P_{\text{trigger}|n,0\text{d}} + p_\text{d} P_{\text{trigger}|n,1\text{d}}]}{P_{\text{trigger}}}. \tag{33}$$

For example, in the case of a triggered PDC source with $p_n = \mu^n(1+\mu)^{-n-1}$ we find that $P_{\text{trigger}|0\text{d}} = \mu\zeta_{\text{c,d}}(1+\mu\zeta_{\text{c,d}})^{-2}$, and $P_{\text{trigger}|1\text{d}} = (1+\mu\zeta_{\text{c,d}})^{-1}$. Hence,

$$P_{\text{trigger}} = \frac{p_\text{d} + \mu\zeta_{\text{c,d}}}{(1+\mu\zeta_{\text{c,d}})^2}. \tag{34}$$

Also, we obtain

$$r_n = \frac{\mu^n(1-\zeta_{\text{c,d}})^{n-1}(1+\mu\zeta_{\text{c,d}})^2}{(1+\mu)^{n+1}(p_\text{d}+\mu\zeta_{\text{c,d}})} \left[(1-p_\text{d})n\zeta_{\text{c,d}} + p_\text{d}(1-\zeta_{\text{c,d}})\right]. \tag{35}$$

In what follows, we will use the notation $\rho_{\text{single}}^{\text{h}}$ and $\rho_{\text{single}}^{\text{v}}$ in order to specify the polarization state of the generated photons.

2. Click pattern distribution

Our starting point is the state of the whole system conditioned on the trigger of both triggered single-photon sources at Bob's PQA,

$$\rho_0|_{\text{double trigger}} = \sigma_{ab} \otimes \rho_{\text{single}}^{\text{h}} \otimes \rho_{\text{single}}^{\text{v}} = \sum_{n=0}^{\infty}\sum_{n_1=0}^{\infty}\sum_{n_2=0}^{\infty} p_n r_{n_1} r_{n_2} |\phi_n\rangle_{ab}\langle\phi_n| \otimes |n_1,n_2\rangle_d\langle n_1,n_2|, \tag{36}$$

where $|n_1,n_2\rangle_d = {d_\text{h}^\dagger}^{n_1}{d_\text{v}^\dagger}^{n_2}/\sqrt{n_1!n_2!}|0\rangle$ and the states $|\phi_n\rangle_{ab}$ are again given by Eq. (4).

Next, we have that the quantum states $|\phi_n\rangle_{ab}$ undergo exactly the same transformation as in the case of the ESR-based setup (see Fig. 1), leading to the states $|\phi_n\rangle_{a_1b_1;f_1g_1}$ given by Eq. (9) right before the interference at the BSM.

On the other hand, the states $|n_1,n_2\rangle_d$ evolve to

$$|\psi_{n_1},\psi_{n_2}\rangle_{d_1;p_1q_1} = \frac{1}{\sqrt{n_1!n_2!}}\sum_{x=0}^{n_1}\sum_{y=0}^{n_2}\binom{n_1}{x}\binom{n_2}{y} T_{\text{c,d}}^{x+y} R_{\text{c,d}}^{n_1+n_2-x-y} {d_\text{h}^\dagger}^x {d_\text{v}^\dagger}^y {p_{1,\text{h}}^\dagger}^{n_1-x} {q_{1,\text{v}}^\dagger}^{n_2-y}|0\rangle, \tag{37}$$

where $T_{\text{c,d}} = \zeta_{\text{c,d}}^{1/2}$ and $R_{\text{c,d}} = (1-\zeta_{\text{c,d}})^{1/2}$ as in App. I A. Here, like in the ESR-based setup, we have modeled the effect of coupling and detection loss by means of a BS with transmittance $\zeta_{\text{c,d}} = \eta_\text{c}\eta_\text{d}$. This means that for the remaining calculations we consider lossless PNR detectors.

Next, the quantum signals coming from both single-photon sources enter a BS of transmittance t. This BS transforms the quantum states $|\psi_{n_1},\psi_{n_2}\rangle_{d_1;p_1q_1}$ into

$$\begin{aligned} |\psi_{n_1},\psi_{n_2}\rangle_{c_1d_2;p_1q_1} &= \frac{1}{\sqrt{n_1!n_2!}}\sum_{x=0}^{n_1}\sum_{y=0}^{n_2}\sum_{z=0}^{x}\sum_{w=0}^{y}\binom{n_1}{x}\binom{n_2}{y}\binom{x}{z}\binom{y}{w} T_{\text{c,d}}^{x+y} R_{\text{c,d}}^{n_1+n_2-x-y} T_\text{t}^{z+w} R_\text{t}^{x+y-z-w} {d_{2,\text{h}}^\dagger}^z {d_{2,\text{v}}^\dagger}^w \\ &\times {c_{1,\text{h}}^\dagger}^{x-z} {c_{1,\text{v}}^\dagger}^{y-w} {p_{1,\text{h}}^\dagger}^{n_1-x} {q_{1,\text{v}}^\dagger}^{n_2-y}|0\rangle, \end{aligned} \tag{38}$$

where $T_{\rm t} = t^{1/2}$. Afterwards, we have that two Hadamard gates rotate the outgoing signals at each output port of the beamsplitter. These Hadamard gates transform the operator $d_{2,\rm h}^{\dagger\ z} d_{2,\rm v}^{\dagger\ w} c_{1,\rm h}^{\dagger\ x-z} c_{1,\rm v}^{\dagger\ y-w}$ as

$$d_{2,\rm h}^{\dagger\ z} d_{2,\rm v}^{\dagger\ w} c_{1,\rm h}^{\dagger\ x-z} c_{1,\rm v}^{\dagger\ y-w} \xrightarrow{\text{Hadamard}} \sum_{a=0}^{z}\sum_{b=0}^{w}\sum_{c=0}^{x-z}\sum_{d=0}^{y-w} \frac{(-1)^{b+d}}{\sqrt{2}^{x+y}} \binom{z}{a}\binom{w}{b}\binom{x-z}{c}\binom{y-w}{d} d_{3,\rm h}^{\dagger\ a+b} d_{3,\rm v}^{\dagger\ z+w-a-b} \times c_{2,\rm h}^{\dagger\ c+d} c_{2,\rm v}^{\dagger\ x+y-z-w-c-d}. \tag{39}$$

By using the same change of variables that we applied to Eq. (12), we have that Eq. (39) can be rewritten as

$$\sum_{u=0}^{z+w}\sum_{r=0}^{x+y-z-w}\sum_{v=\max\{0,u-z\}}^{\min\{w,u\}}\sum_{s=\max\{0,r+z-x\}}^{\min\{y-w,r\}} \frac{(-1)^{v+s}}{\sqrt{2}^{x+y}} \times \binom{z}{u-v}\binom{w}{v}\binom{x-z}{r-s}\binom{y-w}{s} d_{3,\rm h}^{\dagger\ u} d_{3,\rm v}^{\dagger\ z+w-u} c_{2,\rm h}^{\dagger\ r} c_{2,\rm v}^{\dagger\ x+y-z-w-r}. \tag{40}$$

By combining Eqs. (38) and (40), we obtain the quantum states that emerge from the PQA for the teleportation

$$|\psi_{n_1,n_2}\rangle_{c_2 d_3; p_1 q_1} = \frac{1}{\sqrt{n_1! n_2!}} \sum_{x=0}^{n_1}\sum_{y=0}^{n_2}\sum_{z=0}^{x}\sum_{w=0}^{y}\sum_{u=0}^{z+w}\sum_{r=0}^{x+y-z-w}\sum_{v=\max\{0,u-z\}}^{\min\{w,u\}}\sum_{s=\max\{0,r+z-x\}}^{\min\{y-w,r\}} \frac{(-1)^{v+s}}{\sqrt{2}^{x+y}} \binom{n_1}{x}\binom{n_2}{y}\binom{x}{z} \times \binom{y}{w}\binom{z}{u-v}\binom{w}{v}\binom{x-z}{r-s}\binom{y-w}{s} T_{\rm c,d}^{x+y} R_{\rm c,d}^{n_1+n_2-x-y} T_{\rm t}^{z+w} R_{\rm t}^{x+y-z-w} d_{3,\rm h}^{\dagger\ u} d_{3,\rm v}^{\dagger\ z+w-u} c_{2,\rm h}^{\dagger\ r} c_{2,\rm v}^{\dagger\ x+y-z-w-r} p_{1,\rm h}^{\dagger\ n_1-x} q_{1,\rm v}^{\dagger\ n_2-y} |0\rangle. \tag{41}$$

Then, by putting it all together, we have that the overall quantum state prior to the BSM is given by

$$\rho_{\rm BSM}|_{\text{double trigger}} = \sum_{n=0}^{\infty}\sum_{n_1=0}^{\infty}\sum_{n_2=0}^{\infty} p_n r_{n_1} r_{n_2} |\phi_n\rangle_{a_1 b_1; f_1 g_1}\langle\phi_n| \otimes |\psi_{n_1,n_2}\rangle_{c_2 d_3; p_1 q_1}\langle\psi_{n_1,n_2}|, \tag{42}$$

where the pure states $|\phi_n\rangle_{a_1 b_1; f_1 g_1} \otimes |\psi_{n_1,n_2}\rangle_{c_2 d_3; p_1 q_1}$ can be written as

$$|\phi_n\rangle_{a_1 b_1; f_1 g_1} \otimes |\psi_{n_1,n_2}\rangle_{c_2 d_3; p_1 q_1} = \frac{1}{n!\sqrt{(n+1) n_1! n_2!}} \sum_{i=0}^{n}\sum_{x=0}^{n_1}\sum_{y=0}^{n_2}\sum_{j=0}^{i}\sum_{k=0}^{i}\sum_{l=0}^{n-i}\sum_{m=0}^{n-i}\sum_{z=0}^{x}\sum_{w=0}^{y}\sum_{u=0}^{z+w}\sum_{r=0}^{x+y-z-w}\sum_{v=\max\{0,u-z\}}^{\min\{w,u\}} \sum_{s=\max\{0,r+z-x\}}^{\min\{y-w,r\}} \frac{(-1)^{i+v+s}}{\sqrt{2}^{x+y}} \binom{n}{i}\binom{n_1}{x}\binom{n_2}{y}\binom{i}{j}\binom{i}{k}\binom{n-i}{l}\binom{n-i}{m}\binom{x}{z}\binom{y}{w}\binom{z}{u-v}\binom{w}{v}\binom{x-z}{r-s}\binom{y-w}{s} \times T_{\rm c,d}^{j+l+x+y} R_{\rm c,d}^{n+n_1+n_2-j-l-x-y} T_{\rm c,ch,d}^{k+m} R_{\rm c,ch,d}^{n-k-m} T_{\rm t}^{z+w} R_{\rm t}^{x+y-z-w} a_{1,\rm h}^{\dagger\ l} a_{1,\rm v}^{\dagger\ j} b_{1,\rm h}^{\dagger\ k} b_{1,\rm v}^{\dagger\ m} d_{3,\rm h}^{\dagger\ u} d_{3,\rm v}^{\dagger\ z+w-u} c_{2,\rm h}^{\dagger\ r} c_{2,\rm v}^{\dagger\ x+y-z-w-r} \times f_{1,\rm h}^{\dagger\ n-i-l} f_{1,\rm v}^{\dagger\ i-j} g_{1,\rm h}^{\dagger\ i-k} g_{1,\rm v}^{\dagger\ n-i-m} p_{1,\rm h}^{\dagger\ n_1-x} q_{1,\rm v}^{\dagger\ n_2-y} |0\rangle. \tag{43}$$

Next, modes b_1 and c_2 interfere at the 50:50 BS within the PQA. This BS transforms the state $|\phi_n\rangle_{a_1 b_1; f_1 g_1} \otimes |\psi_{n_1,n_2}\rangle_{c_2 d_3; p_1 q_1}$ into the state $|\phi_{n,n_1,n_2}\rangle_{a_1 b_2 c_3 d_3; f_1 g_1 p_1 q_1}$ given by

$$|\phi_{n,n_1,n_2}\rangle_{a_1 b_2 c_3 d_3; f_1 g_1 p_1 q_1} = \frac{1}{n!\sqrt{(n+1) n_1! n_2!}} \sum_{i=0}^{n}\sum_{x=0}^{n_1}\sum_{y=0}^{n_2}\sum_{j=0}^{i}\sum_{k=0}^{i}\sum_{l=0}^{n-i}\sum_{m=0}^{n-i}\sum_{z=0}^{x}\sum_{w=0}^{y}\sum_{u=0}^{z+w}\sum_{r=0}^{x+y-z-w}\sum_{v=\max\{0,u-z\}}^{\min\{w,u\}} \sum_{s=\max\{0,r+z-x\}}^{\min\{y-w,r\}}\sum_{a=0}^{r}\sum_{b=0}^{x+y-z-w-r}\sum_{c=0}^{k}\sum_{d=0}^{m} \frac{(-1)^{i+v+s+a+b}}{\sqrt{2}^{2(x+y)+k+m-z-w}} \binom{n}{i}\binom{n_1}{x}\binom{n_2}{y}\binom{i}{j}\binom{i}{k}\binom{n-i}{l}\binom{n-i}{m}\binom{x}{z}\binom{y}{w} \times \binom{z}{u-v}\binom{w}{v}\binom{x-z}{r-s}\binom{y-w}{s}\binom{r}{a}\binom{x+y-z-w-r}{b}\binom{k}{c}\binom{m}{d} T_{\rm c,d}^{j+l+x+y} R_{\rm c,d}^{n+n_1+n_2-j-l-x-y} T_{\rm c,ch,d}^{k+m} R_{\rm c,ch,d}^{n-k-m} \times T_{\rm t}^{z+w} R_{\rm t}^{x+y-z-w} a_{1,\rm h}^{\dagger\ l} a_{1,\rm v}^{\dagger\ j} d_{3,\rm h}^{\dagger\ u} d_{3,\rm v}^{\dagger\ z+w-u} b_{2,\rm h}^{\dagger\ a+c} b_{2,\rm v}^{\dagger\ b+d} c_{3,\rm h}^{\dagger\ k+r-a-c} c_{3,\rm v}^{\dagger\ x+y+m-z-w-r-b-d} f_{1,\rm h}^{\dagger\ n-i-l} f_{1,\rm v}^{\dagger\ i-j} g_{1,\rm h}^{\dagger\ i-k} \times g_{1,\rm v}^{\dagger\ n-i-m} p_{1,\rm h}^{\dagger\ n_1-x} q_{1,\rm v}^{\dagger\ n_2-y} |0\rangle. \tag{44}$$

As in the case of the ESR-based setup, Alice's and Bob's measurement settings can be incorporated at this stage by rotating the pairs of operators $(a^\dagger_{1,\mathrm{h}}, a^\dagger_{1,\mathrm{v}})$ and $(d^\dagger_{3,\mathrm{h}}, d^\dagger_{3,\mathrm{v}})$ with angles θ_{A} and θ_{B}, respectively. We denote the resulting pure state by $|\phi_{n,n_1,n_2}\rangle^{\theta_{\mathrm{A}},\theta_{\mathrm{B}}}_{a_2b_2c_3d_4;f_1g_1p_1q_1}$, and it is given by

$$
\begin{aligned}
|\phi_{n,n_1,n_2}\rangle^{\theta_{\mathrm{A}},\theta_{\mathrm{B}}}_{a_2b_2c_3d_4;f_1g_1p_1q_1} &= \frac{1}{n!\sqrt{(n+1)n_1!n_2!}} \sum_{i=0}^{n}\sum_{x=0}^{n_1}\sum_{y=0}^{n_2}\sum_{j=0}^{i}\sum_{k=0}^{i}\sum_{l=0}^{n-i}\sum_{m=0}^{n-i}\sum_{z=0}^{x}\sum_{w=0}^{y}\sum_{u=0}^{z+w}\sum_{r=0}^{x+y-z-w}\sum_{v=\max\{0,u-z\}}^{\min\{w,u\}} \\
&\sum_{s=\max\{0,r+z-x\}}^{\min\{y-w,r\}}\sum_{a=0}^{r}\sum_{b=0}^{x+y-z-w-r}\sum_{c=0}^{k}\sum_{d=0}^{m}\sum_{e=0}^{l+j}\sum_{h=\max\{0,e-l\}}^{\min\{e,j\}}\sum_{o=0}^{z+w}\sum_{\tilde{n}=\max\{0,o-u\}}^{\min\{o,z+w-u\}} \frac{(-1)^{i+v+s+a+b+h+\tilde{n}}}{\sqrt{2}^{2(x+y)+k+m-z-w}}\binom{n}{i}\binom{n_1}{x}\binom{n_2}{y}\binom{i}{j} \\
&\times \binom{i}{k}\binom{n-i}{l}\binom{n-i}{m}\binom{x}{z}\binom{y}{w}\binom{z}{u-v}\binom{w}{v}\binom{x-z}{r-s}\binom{y-w}{s}\binom{r}{a}\binom{x+y-z-w-r}{b}\binom{k}{c}\binom{m}{d}\binom{l}{e-h}\binom{j}{h} \\
&\times \binom{u}{o-\tilde{n}}\binom{z+w-u}{\tilde{n}} T_{\mathrm{c,d}}^{j+l+x+y} R_{\mathrm{c,d}}^{n+n_1+n_2-j-l-x-y} T_{\mathrm{c,ch,d}}^{k+m} R_{\mathrm{c,ch,d}}^{n-k-m} T_{\mathrm{t}}^{z+w} R_{\mathrm{t}}^{x+y-z-w} \cos\theta_{\mathrm{A}}{}^{e+j-2h} \sin\theta_{\mathrm{A}}{}^{l+2h-e} \\
&\times \cos\theta_{\mathrm{B}}{}^{o+z+w-u-2\tilde{n}} \sin\theta_{\mathrm{B}}{}^{u+2\tilde{n}-o} a^\dagger_{2,\mathrm{h}}{}^{e} a^\dagger_{2,\mathrm{v}}{}^{l+j-e} d^\dagger_{4,\mathrm{h}}{}^{o} d^\dagger_{4,\mathrm{v}}{}^{z+w-o} b^\dagger_{2,\mathrm{h}}{}^{a+c} b^\dagger_{2,\mathrm{v}}{}^{b+d} c^\dagger_{3,\mathrm{h}}{}^{k+r-a-c} c^\dagger_{3,\mathrm{v}}{}^{x+y+m-z-w-r-b-d} \\
&\times f^\dagger_{1,\mathrm{h}}{}^{n-i-l} f^\dagger_{1,\mathrm{v}}{}^{i-j} g^\dagger_{1,\mathrm{h}}{}^{i-k} g^\dagger_{1,\mathrm{v}}{}^{n-i-m} p^\dagger_{1,\mathrm{h}}{}^{n_1-x} q^\dagger_{1,\mathrm{v}}{}^{n_2-y}|0\rangle. \qquad (45)
\end{aligned}
$$

Then, in an identical way as in the ESR-based setup, the probability $\mathrm{P}\left(\vec{\alpha}|n,n_1,n_2\right)_{\theta_{\mathrm{A}},\theta_{\mathrm{B}}}$ that a specific click pattern $\vec{\alpha} = (\alpha,\beta,\gamma,\delta,\mu,\nu,\tau,\lambda)$ occurs with the state written in Eq. (45) is given by

$$
\mathrm{P}\left(\vec{\alpha}|n,n_1,n_2\right)_{\theta_{\mathrm{A}},\theta_{\mathrm{B}}} = \left\| |\tilde{\phi}_{n,n_1,n_2}\rangle^{\theta_{\mathrm{A}},\theta_{\mathrm{B}}}_{\vec{\alpha};f_1g_1p_1q_1} \right\|^2, \qquad (46)
$$

where the unnormalised state $|\tilde{\phi}_{n,n_1,n_2}\rangle^{\theta_{\mathrm{A}},\theta_{\mathrm{B}}}_{\vec{\alpha};f_1g_1p_1q_1}$ has the form

$$
|\tilde{\phi}_{n,n_1,n_2}\rangle^{\theta_{\mathrm{A}},\theta_{\mathrm{B}}}_{\vec{\alpha};f_1g_1p_1q_1} = \langle\vec{\alpha}|\phi_{n,n_1,n_2}\rangle^{\theta_{\mathrm{A}},\theta_{\mathrm{B}}}_{a_2b_2c_3d_4;f_1g_1p_1q_1}, \qquad (47)
$$

being $|\vec{\alpha}\rangle = |\alpha,\beta,\gamma,\delta,\mu,\nu,\tau,\lambda\rangle$. After addressing the relevant orthogonality relation, given by

$$
\begin{aligned}
&\langle\vec{\alpha}|a^\dagger_{2,\mathrm{h}}{}^{e} a^\dagger_{2,\mathrm{v}}{}^{l+j-e} d^\dagger_{4,\mathrm{h}}{}^{o} d^\dagger_{4,\mathrm{v}}{}^{z+w-o} c^\dagger_{3,\mathrm{h}}{}^{k+r-a-c} c^\dagger_{3,\mathrm{v}}{}^{x+y+m-z-w-r-b-d} b^\dagger_{2,\mathrm{h}}{}^{a+c} b^\dagger_{\mathrm{v}}{}^{b+d}|0\rangle = \\
&(\alpha!\beta!\gamma!\delta!\mu!\nu!\tau!\lambda!)^{1/2}\,\delta^{e}_{\alpha}\delta^{l+j-e}_{\beta}\delta^{o}_{\gamma}\delta^{z+w-o}_{\delta}\delta^{k+r-a-c}_{\mu}\delta^{x+y+m-z-w-r-b-d}_{\nu}\delta^{a+c}_{\tau}\delta^{b+d}_{\lambda}, \qquad (48)
\end{aligned}
$$

one finds that the state $|\tilde{\phi}_{n,n_1,n_2}\rangle^{\theta_{\mathrm{A}},\theta_{\mathrm{B}}}_{\vec{\alpha};f_1g_1p_1q_1}$ can be written as

$$
\begin{aligned}
|\tilde{\phi}_{n,n_1,n_2}\rangle^{\theta_{\mathrm{A}},\theta_{\mathrm{B}}}_{\vec{\alpha};f_1g_1p_1q_1} &= \frac{1}{n!}\left[\frac{\alpha!\beta!\gamma!\delta!\mu!\nu!\tau!\lambda!}{(n+1)n_1!n_2!2^{\mu+\nu+\tau+\lambda}}\right]^{\frac{1}{2}} T_{\mathrm{c,d}}^{\alpha+\beta} R_{\mathrm{c,d}}^{n+n_1+n_2-\alpha-\beta} T_{\mathrm{c,ch,d}}^{\gamma+\delta+\mu+\nu+\tau+\lambda} R_{\mathrm{c,ch,d}}^{n-\gamma-\delta-\mu-\nu-\tau-\lambda} T_{\mathrm{t}}^{\gamma+\delta} \\
&\times R_{\mathrm{t}}^{-\gamma-\delta} \cos\theta_{\mathrm{A}}{}^{\alpha} \sin\theta_{\mathrm{A}}{}^{\beta} \cos\theta_{\mathrm{B}}{}^{2\gamma+\delta} \sin\theta_{\mathrm{B}}{}^{-\gamma} \sum_{i=0}^{n}\sum_{x=0}^{n_1}\sum_{y=0}^{n_2} \sum_{k=\max\{0,\gamma+\delta+\mu+\tau-x-y,\gamma+\delta+\mu+\nu+\tau+\lambda+i-n-x-y\}}^{\min\{i,\mu+\tau,\gamma+\delta+\mu+\nu+\tau+\lambda-x-y\}} \\
&\times \sum_{j=\max\{0,\alpha+\beta+i-n\}}^{\min\{i,\alpha+\beta\}} \sum_{w=\max\{0,\gamma+\delta-x\}}^{\min\{y,\gamma+\delta\}} \sum_{u=0}^{\gamma+\delta} \sum_{v=\max\{0,u+w-\gamma-\delta\}}^{\min\{w,u\}} \sum_{s=\max\{0,\mu+\tau+\gamma+\delta-x-k-w\}}^{\min\{y-w,\mu+\tau-k\}} \sum_{a=\max\{0,\tau-k\}}^{\min\{\tau,\mu+\tau-k\}} \\
&\times \sum_{b=\max\{0,x+y+k-\gamma-\delta-\mu-\nu-\tau\}}^{\min\{\lambda,x+y+k-\gamma-\delta-\mu-\tau\}} \sum_{h=\max\{0,j-\beta\}}^{\min\{\alpha,j\}} \sum_{\tilde{n}=\max\{0,\gamma-u\}}^{\min\{\gamma,\gamma+\delta-u\}} \frac{(-1)^{i+v+s+a+b+h+\tilde{n}}}{\sqrt{2}^{x+y}} \binom{n}{i}\binom{n_1}{x}\binom{n_2}{y}\binom{i}{j}\binom{i}{k}\binom{n-i}{\alpha+\beta-j} \\
&\times \binom{x}{\gamma+\delta-w}\binom{y}{w}\binom{y-w}{s}\binom{n-i}{\gamma+\delta+\mu+\nu+\tau+\lambda-x-y-k}\binom{\gamma+\delta-w}{u-v}\binom{w}{v}\binom{x+w-\gamma-\delta}{\mu+\tau-k-s}\binom{\mu+\tau-k}{a} \\
&\times \binom{j}{h}\binom{u}{\gamma-\tilde{n}}\binom{\gamma+\delta-u}{\tilde{n}}\binom{k}{\tau-a}\binom{\gamma+\delta+\mu+\nu+\tau+\lambda-x-y-k}{\lambda-b}\binom{\alpha+\beta-j}{\alpha-h}\binom{x+y+k-\gamma-\delta-\mu-\tau}{b} \\
&\times \left(\frac{T_{\mathrm{c,d}}R_{\mathrm{c,ch,d}}R_{\mathrm{t}}}{T_{\mathrm{c,ch,d}}R_{\mathrm{c,d}}}\right)^{x+y} \left(\frac{\sin\theta_{\mathrm{A}}}{\cos\theta_{\mathrm{A}}}\right)^{2h-j} \left(\frac{\sin\theta_{\mathrm{B}}}{\cos\theta_{\mathrm{B}}}\right)^{2\tilde{n}+u} f^\dagger_{1,\mathrm{h}}{}^{n+j-i-\alpha-\beta} f^\dagger_{1,\mathrm{v}}{}^{i-j} g^\dagger_{1,\mathrm{h}}{}^{i-k} g^\dagger_{1,\mathrm{v}}{}^{n+x+y+k-i-\gamma-\delta-\mu-\nu-\tau-\lambda} \\
&\times p^\dagger_{1,\mathrm{h}}{}^{n_1-x} q^\dagger_{1,\mathrm{v}}{}^{n_2-y}|0\rangle. \qquad (49)
\end{aligned}
$$

Finally, by taking the squared norm of the previous state as decribed in Eq. (46), we obtain that

$$
\begin{aligned}
\mathrm{P}\left(\vec{\alpha}|n,n_1,n_2\right)_{\theta_{\mathrm{A}},\theta_{\mathrm{B}}} = {} & \frac{\alpha!\beta!\gamma!\delta!\mu!\nu!\tau!\lambda!}{(n+1)2^{\mu+\nu+\tau+\lambda}}\zeta_{\mathrm{c,d}}^{\alpha+\beta}(1-\zeta_{\mathrm{c,d}})^{n+n_1+n_2-\alpha-\beta}\zeta_{\mathrm{c,ch,d}}^{\gamma+\delta+\mu+\nu+\tau+\lambda}(1-\zeta_{\mathrm{c,ch,d}})^{n-\gamma-\delta-\mu-\nu-\tau-\lambda} \\
& \times\left(\frac{t}{1-t}\right)^{\gamma+\delta}\cos\theta_{\mathrm{A}}{}^{2\alpha}\sin\theta_{\mathrm{A}}{}^{2\beta}\cos\theta_{\mathrm{B}}{}^{2(2\gamma+\delta)}\sin\theta_{\mathrm{B}}{}^{-2\gamma}\sum_{i=0}^{n}\sum_{\Delta=-i}^{n-i}\sum_{x=0}^{n_1}\sum_{y=0}^{n_2}\sum_{j=\max\{0,\alpha+\beta+i-n,-\Delta\}}^{\min\{i,\alpha+\beta,\alpha+\beta-\Delta\}} \\
& \times\sum_{k=\max\{0,\gamma+\delta+\mu+\tau-x-y,\gamma+\delta+\mu+\nu+\tau+\lambda+i-n-x-y,-\Delta,\gamma+\delta+\mu+\tau-x-y-\Delta\}}^{\min\{i,\mu+\tau,\gamma+\delta+\mu+\nu+\tau+\lambda-x-y,\mu+\tau-\Delta,\gamma+\delta+\mu+\nu+\tau+\lambda-x-y-\Delta\}}\sum_{w=\max\{0,\gamma+\delta-x\}}^{\min\{y,\gamma+\delta\}}\sum_{W=\max\{0,\gamma+\delta-x\}}^{\min\{y,\gamma+\delta\}}\sum_{u=0}^{\gamma+\delta}\sum_{U=0}^{\gamma+\delta} \\
& \times\sum_{v=\max\{0,u+w-\gamma-\delta\}}^{\min\{w,u\}}\sum_{V=\max\{0,U+W-\gamma-\delta\}}^{\min\{W,U\}}\sum_{s=\max\{0,\mu+\tau+\gamma+\delta-x-k-w\}}^{\min\{y-w,\mu+\tau-k\}}\sum_{S=\max\{0,\mu+\tau+\gamma+\delta-x-k-\Delta-W\}}^{\min\{y-W,\mu+\tau-k-\Delta\}}\sum_{a=\max\{0,\tau-k\}}^{\min\{\tau,\mu+\tau-k\}} \\
& \times\sum_{A=\max\{0,\tau-k-\Delta\}}^{\min\{\tau,\mu+\tau-k-\Delta\}}\sum_{b=\max\{0,x+y+k-\gamma-\delta-\mu-\nu-\tau\}}^{\min\{\lambda,x+y+k-\gamma-\delta-\mu-\tau\}}\sum_{B=\max\{0,x+y+k+\Delta-\gamma-\delta-\mu-\nu-\tau\}}^{\min\{\lambda,x+y+k+\Delta-\gamma-\delta-\mu-\tau\}}\sum_{h=\max\{0,j-\beta\}}^{\min\{\alpha,j\}}\sum_{H=\max\{0,j+\Delta-\beta\}}^{\min\{\alpha,j+\Delta\}} \\
& \times\sum_{\tilde{n}=\max\{0,\gamma-u\}}^{\min\{\gamma,\gamma+\delta-u\}}\sum_{\tilde{N}=\max\{0,\gamma-U\}}^{\min\{\gamma,\gamma+\delta-U\}}(-1)^{v+s+a+b+h+\tilde{n}+V+S+A+B+H+\tilde{N}+\Delta}\left(\frac{\zeta_{\mathrm{c,d}}(1-\zeta_{\mathrm{c,ch,d}})(1-t)}{2\zeta_{\mathrm{c,ch,d}}(1-\zeta_{\mathrm{c,d}})}\right)^{x+y} \\
& \times\left(\frac{\sin\theta_{\mathrm{A}}}{\cos\theta_{\mathrm{A}}}\right)^{2(h+H-j)-\Delta}\left(\frac{\sin\theta_{\mathrm{B}}}{\cos\theta_{\mathrm{B}}}\right)^{2(\tilde{n}+\tilde{N})+u+U} \\
& \times\Upsilon(n,n_1,n_2,i,x,y,j,k,w,W,u,U,v,V,s,S,a,A,b,B,h,H,\tilde{n},\tilde{N},\Delta,\alpha,\beta,\gamma,\delta,\mu,\nu,\tau,\lambda),
\end{aligned}
\tag{50}
$$

where

$$
\begin{aligned}
& \Upsilon(n,n_1,n_2,i,x,y,j,k,w,W,u,U,v,V,s,S,a,A,b,B,h,H,\tilde{n},\tilde{N},\Delta,\alpha,\beta,\gamma,\delta,\mu,\nu,\tau,\lambda) = n_1!n_2!i!(i+\Delta)!(n-i)! \\
& \times(n-i-\Delta)!\binom{\mu+\tau-k}{a}\binom{\mu+\tau-k-\Delta}{A}\binom{x+y+k-\gamma-\delta-\mu-\tau}{b}\binom{x+y+k+\Delta-\gamma-\delta-\mu-\tau}{B}\binom{u}{\gamma-\tilde{n}} \\
& \times\binom{U}{\gamma-\tilde{N}}\binom{\gamma+\delta-u}{\tilde{n}}\binom{\gamma+\delta-U}{\tilde{N}}\left[(\alpha-h)!(\alpha-H)!(\beta+h-j)!(\beta+H-j-\Delta)!h!H!(j-h)!(j+\Delta-H)!\right]^{-1} \\
& \times\frac{\left[(n_1-x)!(n_2-y)!(i-j)!(i-k)!(n+j-i-\alpha-\beta)!(n+x+y+k-i-\gamma-\delta-\mu-\nu-\tau-\lambda)!(u-v)!\right]^{-1}}{(\gamma+\delta+v-u-w)!(\gamma+\delta+V-U-W)!v!V!(w-v)!(W-V)!(\mu+\tau-k-s)!(\mu+\tau-k-\Delta-S)!} \\
& \times\frac{\left[(x+w+k+s-\gamma-\delta-\mu-\tau)!(x+W+k+\Delta+S-\gamma-\delta-\mu-\tau)!(y-w-s)!(y-W-S)!s!S!(\tau-a)!\right]^{-1}}{(k+a-\tau)!(k+\Delta+A-\tau)!(b+\gamma+\delta+\mu+\nu+\tau-x-y-k)!(B+\gamma+\delta+\mu+\nu+\tau-x-y-k-\Delta)!} \\
& \times\frac{\left[(U-V)!(\tau-A)!\right]^{-1}}{(\lambda-b)!(\lambda-B)!}.
\end{aligned}
\tag{51}
$$

The normalization condition $\sum_{\vec{\alpha}}\mathrm{P}\left(\vec{\alpha}|n,n_1,n_2\right)_{\theta_{\mathrm{A}},\theta_{\mathrm{B}}}=1$ holds for any set of physical parameters θ_{A}, θ_{B}, $\zeta_{\mathrm{c,d}}$, $\zeta_{\mathrm{c,ch,d}}$ and t, and only those click patterns $\vec{\alpha}$ such that $\alpha+\beta\leq n$, $\gamma+\delta\leq n_1+n_2$ and $\gamma+\delta+\mu+\nu+\tau+\lambda\leq n+n_1+n_2$ have a nonzero probability to happen due to the fact that for the moment we have disregarded dark counts.

As a final step, we need to define the click pattern distribution in the noisy scenario, $\tilde{\mathrm{p}}\left(\vec{\alpha}|n,n_1,n_2\right)_{\theta_{\mathrm{A}},\theta_{\mathrm{B}}}$, as well as the post-processed click pattern distribution, $\mathbf{P}(A_{\vec{\alpha}}|n,n_1,n_2)_{\theta_{\mathrm{A}},\theta_{\mathrm{B}}}$ (with $A_{\vec{\alpha}}=(A_{\mathrm{A}},A_{\mathrm{B}},\mu,\nu,\tau,\lambda)$). This is exactly analogous to what we did for the ESR-based setup in App. I A 1, and we omit the details here for simplicity. The only difference is that no permutation step $\alpha\leftrightarrow\beta$ is performed in this case. This is so because, conditioned on a successful heralding event, Alice's and Bob's outcomes are expected to be directly correlated in this case.

3. Parameters P_{SH}, $Q|_{\mathrm{SH}}$ and $\omega|_{\mathrm{SH}}$

To determine P_{SH}, we note that any given detection event is discarded unless a trigger is observed at the idler mode of both single photon sources and, at the same time, a success occurs at the PQA. Therefore, and due to the

symmetries of the channel model under consideration, we can define, say the event $\Omega = \{(\mu, \nu, \tau, \lambda) = (1, 1, 0, 0)\}$ (as we did in App. I A 2 and thus the overall success probability is given by

$$P_{\rm SH} = 4P_{\Omega,\text{double trigger}} = 4P^2_{\text{trigger}}P_{\Omega|\text{double trigger}}, \tag{52}$$

where the conditional probability $P_{\Omega|\text{double trigger}}$ has the form

$$P_{\Omega|\text{double trigger}} = \sum_{A_{\rm A}, A_{\rm B}} \mathbf{P}(A_{\rm A}, A_{\rm B}, \Omega|\text{double trigger})_{\theta_{\rm A},\theta_{\rm B}} = \sum_{\rm n, n_1, n_2} \rm p_n r_{n_1} r_{n_2} \sum_{\rm A_A, A_B} \mathbf{P}(\rm A_A, A_B, \Omega|n, n_1, n_2)_{\theta_{\rm A},\theta_{\rm B}}, \tag{53}$$

with $A_{\rm A}, A_{\rm B} \in \{0, 1\}$. On the other hand, we have that the conditional QBER reads

$$Q|_{\Omega,\text{double trigger}} = \frac{\mathbf{P}(0,1,\Omega)_{0,0} + \mathbf{P}(1,0,\Omega)_{0,0}}{P_{\Omega|\text{double trigger}}} = \frac{1}{P_{\Omega|\text{double trigger}}} \sum_{n,n_1,n_2} p_n r_{n_1} r_{n_2} \left[\mathbf{P}(0,1,\Omega|n,n_1,n_2)_{0,0} + \mathbf{P}(1,0,\Omega|n,n_1,n_2)_{0,0}\right]. \tag{54}$$

Similarly, the conditional winning probability reads $\omega|_{\Omega,\text{double trigger}} = S|_{\Omega,\text{double trigger}}/8 + 1/2$, where

$$S|_{\Omega,\text{double trigger}} = E_{0,-\frac{\pi}{8}}|_\Omega + E_{0,\frac{\pi}{8}}|_\Omega - E_{\frac{\pi}{4},-\frac{\pi}{8}}|_\Omega + E_{\frac{\pi}{4},\frac{\pi}{8}}|_\Omega \tag{55}$$

and the quantities $E_{\theta_{\rm A},\theta_{\rm B}}|_\Omega$ are given by

$$E_{\theta_{\rm A},\theta_{\rm B}}|_\Omega = \frac{2}{P_{\Omega|\text{double trigger}}} \sum_{n,n_1,n_2} p_n r_{n_1} r_{n_2} \times [\mathbf{P}(0,0,\Omega|n,n_1,n_2)_{\theta_{\rm A},\theta_{\rm B}} + \mathbf{P}(1,1,\Omega|n,n_1,n_2)_{\theta_{\rm A},\theta_{\rm B}}] - 1. \tag{56}$$

Note that in Eq. (55) the summand that carries the minus sign is different from that of Eq. (29). As already explained in App. I A 2, the definition of the conditional CHSH violation depends on the particular Bell pair shared by the parties after a successful BSM [4].

C. DIQKD with PDC sources

Here, we particularise the calculations of the previous Appendixes (App. I A and App. I B) to the case of PDC sources, in order to analyze their performance and experimental requirements for DIQKD. Precisely, we suppose that the quantum state ρ_{ab} emitted by Alice's entanglement source is described by Eqs. (5) and (4), with the statistics p_n given by

$$p_n = \frac{(n+1)\lambda^n}{(1+\lambda)^{n+2}}. \tag{57}$$

The quantum state ρ_{bc} generated by the entanglement source within the qubit amplifier depends on the architecture we consider. In the case of a PQA, we will assume that the single-photon states $\rho^{\rm h}_{\rm single}$ and $\rho^{\rm v}_{\rm single}$ have the form $\rho_{\rm single} = \sum_{n=0}^{\infty} r_n|n\rangle\langle n|$ (see App. I B 1) with the statistics r_n given by Eq. (35), *i.e.*, they are generated with a triggered single-photon PDC source in combination with PNR detectors. The corresponding trigger probability is thus given by Eq. (34). In the case of an ESR architecture, we will asume that the state ρ_{bc} is directly generated with a PDC source, *i.e.*, it also has the form given by Eqs. (5) and (4) with the statistics p_n given by Eq. (57).

To simplify the numerics, in our simulations below we consider a contribution of up to three photon pairs per source. That is, we set $p_n = 0$ in Eq. (57) for all $n \geq 4$ and we choose $p_3 = 1 - p_0 - p_1 - p_2$. Likewise, we do the same with the statistics r_n in Eq. (35). This is a reasonable approximation when the optimal intensities of the light sources are sufficiently small, which, indeed, is what we expect and observe in our simulations.

1. No channel loss

We start our analysis by evaluating the minimum value of the data block size, $n_{\rm SH}$, required to obtain a zero-distance secret key rate $K|_{L=0} \geq 10^{-10}$, as a function of the detection and coupling efficiency, $\eta_{\rm c,d}$. This is what we refer to as the "critical lines" in the main text.

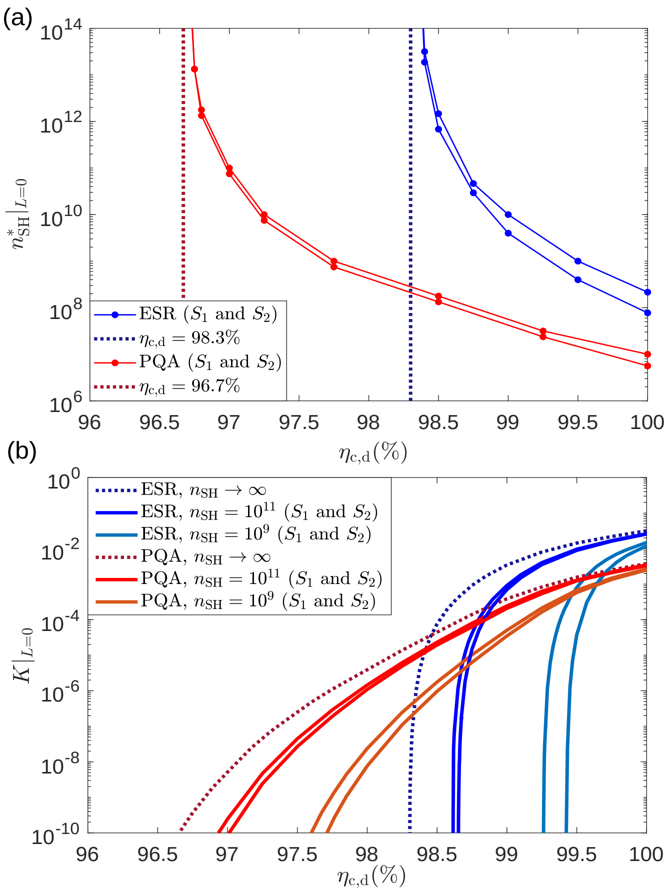

FIG. 3: Performance evaluation of DIQKD with PDC sources. Bluish (reddish) lines are used for the ESR (PQA) architecture. (a) Minimum value of the detection and coupling efficiency, $\eta_{\rm c,d}$, and minimum value of the data block size, $n_{\rm SH}$, required to obtain a zero-distance secret key rate $K|_{L=0} \geq 10^{-10}$. Both sets of security requirements, S_1 and S_1, are compared for each qubit amplifier. Any combination of parameters $\eta_{\rm c,d}$ and $n_{\rm SH}$ must be above the lower (upper) lines to achieve a secret key rate above the threshold value with the security requirements given by the sets S_1 (S_2). The dotted blue (red) vertical line indicates the (asymptotic) minimum efficiency, $\eta_{\rm c,d} \approx 98.3\%$ ($\eta_{\rm c,d} \approx 96.7\%$), which is the smallest detection efficiency that delivers a zero-distance asymptotic secret key rate $K_\infty|_{L=0} \geq 10^{-10}$ when using the ESR (PQA) architecture. (b) Zero-distance secret key rate, $K|_{L=0}$, as a function of $\eta_{\rm c,d}$ for various values of the data block size $n_{\rm SH}$. For each qubit amplifier, three different block sizes are considered: $n_{\rm SH} \to \infty$, $n_{\rm SH} = 10^{11}$ and $n_{\rm SH} = 10^{9}$. The finite secret key rates appear in pairs of solid lines, one for the security set S_1 (upper line) and another one for the security set S_2 (lower line). The asymptotic secret key rates corresponding to $n_{\rm SH} \to \infty$ are illustrated with dotted lines.

Also, the same two sets of security settings presented there are considered here, S_1 ($\epsilon_{\rm sec} = 10^{-5}, \epsilon_{\rm cor} = 10^{-10}$, $\epsilon_{\rm rob} = 10^{-2}$) and S_2 ($\epsilon_{\rm sec} = 10^{-9}, \epsilon_{\rm cor} = 10^{-15}$, $\epsilon_{\rm rob} = 10^{-3}$), and the dark count rate of the detectors is set to zero as well. By definition, any combination of $\eta_{\rm c,d}$ and $n_{\rm SH}$ lying below the critical lines illustrated in the figure leads to a minuscule (if not zero) secret key rate below 10^{-10}. The blue (red) solid lines correspond to the ESR (PQA) architecture, and in each case the lower (upper) line uses the security requirements S_1 (S_2). Fig. 3(a) shows clearly that the ESR architecture demands much larger data block sizes and detection and coupling efficiencies than the PQA architecture in this case. This can be understood if one compares $P_{\rm SH}$ and $K|_{\rm SH}$ separately for both qubit amplifiers: while the ESR has a considerably larger success probability in the relevant efficiency regime, its conditional secret key rate is lower. This suggests that the PQA performs better at filtering genuine entanglement, and thus it is more robust to efficiency decrease and statistical fluctuations (partly due to the availability of an extra tunable parameter t). Also, as already observed in the case of ideal sources, we note that using the security set S_2 (instead of that given by S_1) does not affect much the minimum data block size, despite the significant difference that exists between both sets in terms of security requirements.

The dotted blue (red) vertical line shown in Fig. 3(a) corresponds to the (asymptotic) minimum efficiency, $\eta_{\mathrm{c,d}} \approx 98.3\%$ ($\eta_{\mathrm{c,d}} \approx 96.7\%$), that is necessary to obtain a secret key rate above the threshold value with the ESR-based qubit amplifier (PQA) when the block size n_{SH} tends to infinity. As expected, these values are much higher than those required for the case of ideal sources (see the main text), especially for the ESR. These results are also in accordance with the results reported in [1], as that work also considers a similar setup and device models like here, though the analysis of [1] is restricted to the asymptotic key rate scenario. From Fig. 3(a) we observe that when n_{SH} decreases, the minimum value of $\eta_{\mathrm{c,d}}$ increases even further as expected. For example, if $n_{\mathrm{SH}} = 10^{11}$ and we focus on, say, the weaker set of security requirements, S_1, we find that the minimum values of $\eta_{\mathrm{c,d}}$ are about 98.6% and 97% for the ESR and for the PQA, respectively.

Fig. 3(b) illustrates the zero-distance secret key rate, $K|_{L=0}$, as a function of $\eta_{\mathrm{c,d}}$ for varios values of the block size n_{SH}. This figure shows that, in the absence of channel loss, an ESR-based qubit amplifier outperforms a PQA in the regime of very high detection and coupling efficiencies, while in principle the PQA can tolerate slightly lower values of $\eta_{\mathrm{c,d}}$, as we have already seen in Fig. 3(a). In any case, the minimum value of $\eta_{\mathrm{c,d}}$, especially for the ESR, seems to be already probably too high to have practical relevance. The main reasons for this behaviour are twofold. First, the vacuum signals emitted by the light sources significantly reduce the probability to have a successful heralding event in the amplifier. And, second, multiple photon pairs are also responsable for spurious heralding events which increase (decrease) $Q|_{\mathrm{SH}}$ ($\omega|_{\mathrm{SH}}$) and thus decrease the resulting K. The higher $\eta_{\mathrm{c,d}}$ is, the higher the number of multiple photon pairs that can be filtered out by Alice and Bob's PNR detectors and, therefore, the better the resulting performance. As already mentioned above, in our simulations we optimise over the intensities of the different light sources and, as expected, the optimal intensities decrease when $\eta_{\mathrm{c,d}}$ decreases in order to reduce the likelihood of emitting multiple photon pairs.

2. Channel loss

Next, we consider the effect of the channel loss and we set $p_{\mathrm{d}} = 10^{-7}$, as detector noise becomes more relevant as the distance increases.

The results are illustrated in Fig. 4, which shows the secret key rate K as a function of Λ for various values of $\eta_{\mathrm{c,d}}$ and n_{SH}. More precisely, Figs. 4(a) and 4(b) are respectively devoted to the ESR-architecture and to the PQA-architecture. For each case, we assume two values of $\eta_{\mathrm{c,d}}$: the ideal one $\eta_{\mathrm{c,d}} = 100\%$, and another one close to the threshold value of the ESR, say, $\eta_{\mathrm{c,d}} = 98.7\%$, for comparison purposes. Regarding the block size n_{SH}, we set it to a value near the critical line in each case. That is, we consider the pairs $(\eta_{\mathrm{c,d}}, n_{\mathrm{SH}}) \in \{(100\%, 10^9), (98.7\%, 10^{11})\}$ for the ESR, and $(\eta_{\mathrm{c,d}}, n_{\mathrm{SH}}) \in \{(100\%, 10^7), (98.7\%, 10^9)\}$ for the PQA. Also, Fig. 4 includes the results for the sets S_1 and S_2 of security requirements, as well as the asymptotic curves corresponding to K_∞. These latter curves serve as upper bounds to the attainable finite-key rates for each $\eta_{\mathrm{c,d}}$.

Generally speaking, we observe that, in the presence of channel loss, the performance of DIQKD with PDC sources is again significantly worse than that achievable with ideal sources. The reason for this, as already explained above, is the presence of vacuum and multiple photon pairs, which require that the intensities of the sources are quite low to palliate their negative effect. Indeed, in the case of an ESR, we find that even when $n_{\mathrm{SH}} = 10^{11}$, the detection and coupling efficiency is as high as 98.7%, and the weaker set of security requirements is considered (S_1), the resulting secret key rate is already as low as $K \approx 10^{-10}$ for a channel loss of only 9 dB. Similarly, for the same value of $\eta_{\mathrm{c,d}}$ and a block size $n_{\mathrm{SH}} = 10^9$, we find that the PQA can only tolerate about 14 dB channel loss. In this regard, we remark that setting n_{SH} to a different value for each qubit amplifier does not necessarily lead to an unfair comparison between them, as the average number of signals, $\langle N \rangle$, required to gather a particular block size is different in both cases. This is discussed in more detail in the following subsection.

Finally, from Fig. 4 we observe again that increasing the security requirements from S_1 to S_2 does not affect the system performance significantly. Also, we note that if Bob did not use a qubit amplifier, the maximum possible value of Λ in this scenario would be as low as $\Lambda \lessapprox 0.4$ dB, even if one sets $\eta_{\mathrm{c,d}} = 100\%$ and $n_{\mathrm{SH}} \to \infty$ (see App. I D for further details).

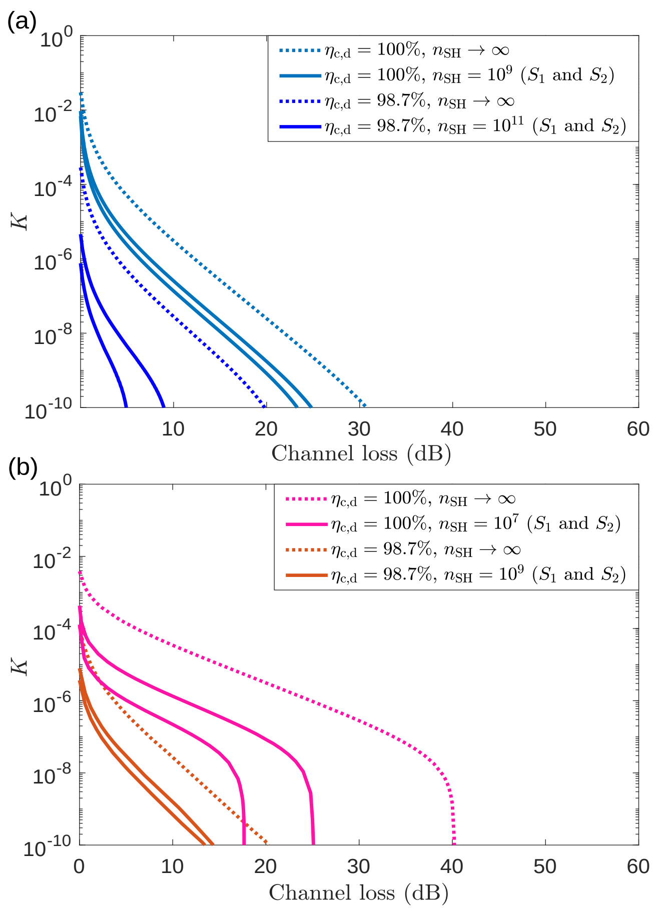

FIG. 4: Secret key rate K as a function of the overall channel loss Λ measured in dB for the case of PDC sources. Figure (a) corresponds to the ESR architecture and figure (b) to the PQA architecture. For each qubit amplifier, we use two different detection and coupling efficiencies, $\eta_{c,d} = 100\%$ and $\eta_{c,d} = 98.7\%$, each of them tagged with a different color. For each value of the efficiency, we plot the asymptotic secret key rate K_∞ (dotted line), together with two finite-key rates for different values of n_{SH} (solid lines). Each finite-key rate is plotted twice, one line corresponds to the security settings S_1 and the other line to the security settings S_2. We take $n_{SH} \in \{10^9, 10^{11}\}$ for the ESR-based qubit amplifier and $n_{SH} \in \{10^7, 10^9\}$ for the PQA. By increasing the value of $\eta_{c,d}$ and/or n_{SH} the finite-key rates approach those of the optimal scenario, which corresponds to K_∞ assuming $\eta_{c,d} = 100\%$.

3. Time constraints

To conclude this part, we now consider the duration of a DIQKD session with PDC sources. As we show below, in this case the time requirements are much more demanding than in the scenario with ideal sources. As discussed above, this happens because the optimal intensity of Alice's source, ρ_{ab}, is quite low in the high loss regime. As a result, the average number, $\langle N \rangle$, of signals that Alice has to send Bob to achieve n_{SH} successful heralding events turns out to be quite high.

This is illustrated in Fig. 5, which shows the value of $\langle N \rangle$ as a function of $\eta_{c,d}$ at $L = 0$ km. As in Fig. 3, dark counts are disregarded here because their effect is negligible. Also, we consider both sets of security requirements (S_1 and S_2) and two different block sizes for each qubit amplifier: $n_{SH} = 10^{11}$ and $n_{SH} = 10^9$. We note that the case $\langle N \rangle |_{L=0} \geq 10^{15}$ leads to DIQKD sessions that would take longer than one day even with 10 GHz PDC sources. Actually, if the conditions $\langle N \rangle |_{L=0} < 10^{15}$ (see Fig. 5) and $K \geq 10^{-10}$ (see Fig. 3) are imposed, one finds that the detection efficiency must satisfy $\eta_{c,d} \gtrapprox 98\%$, irrespectively of the qubit amplifier and the data block size. This means that the examples shown in Fig. 3 for the PQA where $K \geq 10^{-10}$ is possible for $\eta_{c,d} < 98\%$, are probably not too practical, as they require too long DIQKD sessions.

Obviously, if one considers the case of nonzero channel loss, the time constraints become sharper. This is illustrated

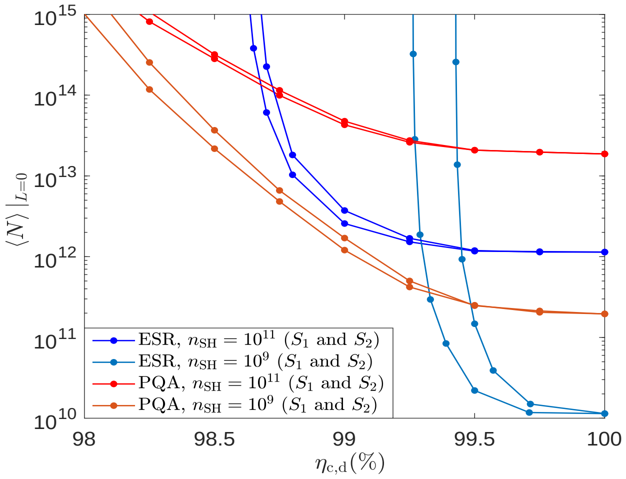

FIG. 5: Average number of transmitted signals, $\langle N\rangle|_{L=0}$, that Alice needs to send Bob to collect a data block size equal to n_{SH} when using PDC sources, as a function of the detection and coupling efficiency $\eta_{\mathrm{c,d}}$ at $L=0$ km. The free experimental and security parameters are set to the values that optimise the secret key rate given by Fig. 3(b). The figure considers two different data block sizes, *i.e.*, $n_{\mathrm{SH}}=10^9$ and $n_{\mathrm{SH}}=10^{11}$. Also, we consider both sets of security requirements (S_1 and S_2) and, like in Fig. 3, here we disregard dark counts because their effect at $L=0$ km is negligible.

S_1	$\eta_{\mathrm{c,d}}$	n_{SH}	Λ	K	$\langle N\rangle$
ESR	100%	10^9	13 dB	5.7×10^{-8}	10^{15}
ESR	98.7%	10^{11}	1 dB	5.9×10^{-7}	10^{15}
PQA	100%	10^7	24 dB	1.1×10^{-8}	1.7×10^{13}
PQA	98.7%	10^9	4 dB	5.5×10^{-8}	10^{15}

S_2	$\eta_{\mathrm{c,d}}$	n_{SH}	Λ	K	$\langle N\rangle$
ESR	100%	10^9	12 dB	4.9×10^{-8}	10^{15}
ESR	98.7%	10^{11}	0 dB	2.9×10^{-7}	10^{15}
PQA	100%	10^7	17 dB	6.9×10^{-9}	8.6×10^{12}
PQA	98.7%	10^9	3 dB	5.7×10^{-8}	10^{15}

TABLE I: Maximum value of the channel loss, Λ, before either $\langle N\rangle\geq10^{15}$ or the secret key rate K starts dropping down to zero, depending on the pair $(\eta_{\mathrm{c,d}},n_{\mathrm{SH}})$ and on the qubit amplifier. The considered detection and coupling efficiencies, as well as the data block sizes, correspond to the finite-key rates illustrated in Fig. 4 with security settings S_1 and S_2. As shown by the table, in the case of the PQA with $\eta_{\mathrm{c,d}}=100\%$ and $n_{\mathrm{SH}}=10^7$, the secret key rate drops down to zero before $\langle N\rangle$ exceeds 10^{15} signals. To be precise, the cutoff for S_1 (S_2) roughly occurs at $\Lambda=24$ dB (17 dB), and the corresponding $\langle N\rangle$ is still 1.7×10^{13} (8.6×10^{12}).

in Table I, which shows the maximum value of the channel loss, Λ, for which $\langle N\rangle\leq10^{15}$ for various pairs $(\eta_{\mathrm{c,d}},n_{\mathrm{SH}})$ previously evaluated in Fig. 4. For instance, when $\eta_{\mathrm{c,d}}=98.7\%$, and assuming the ESR (PQA) architecture, the maximum Λ decreases from 9 (14) dB as shown in Fig. 4 to roughly 1 (4) dB for $n_{\mathrm{SH}}=10^{11}$ (10^9).

Indeed, in App. I E we show that, due to similar time constraints, locating the entanglement source ρ_{ab} in the middle of the channel between Alice and Bob, and furnishing both Alice and Bob with a qubit amplifier, does not seem to improve the performance that can be obtained when Alice holds the source and only Bob holds a qubit amplifier, at least in the case of PDC sources.

D. DIQKD without an heralding mechanism

In this Appendix, we calculate the maximum channel loss that a photonic DIQKD implementation can tolerate in the absence of an heralding mechanism. For that purpose, we consider the setup where Bob does not hold a qubit amplifier in his lab, as shown by Fig. 6. Also, since we are interested in the maximum achievable distance, we further assume perfect coupling and detection efficiencies, *i.e.*, $\eta_{\mathrm{c}}=\eta_{\mathrm{d}}=1$, and no detector noise, *i.e.*, $p_{\mathrm{d}}=0$. In this way, channel loss is the only source of loss that we contemplate, modeled by a transmission efficiency $\eta_{\mathrm{ch}}=10^{-\Lambda/10}$ as usual.

In the case of a perfect entanglement source, $\rho_{ab}=|\phi_1\rangle_{ab}\langle\phi_1|$ (see Eq. (4)) we have that the parameters of the honest implementation are simply given by $Q=(1-\eta_{\mathrm{ch}})/2$ and $\omega=(\sqrt{2}\eta_{\mathrm{ch}}+2)/4$, so that the maximum tolerated

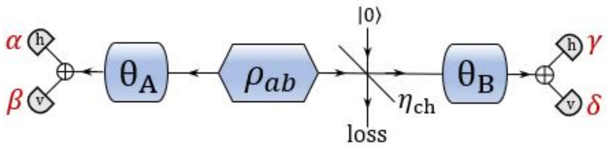

FIG. 6: Schematic of the DIQKD setup without a qubit amplifier. Since we are interested in determining the maximum achievable distance, detection and coupling efficiencies are assumed to be perfect, *i.e.*, $\eta_{\rm c} = \eta_{\rm d} = 1$, and detector noise is set to zero, *i.e.*, $p_{\rm d} = 0$. Again, $\eta_{\rm ch}$ tags the transmission efficiency of the channel, $\eta_{\rm ch} = 10^{-\Lambda/10}$, the symbol "$\oplus$" represents the PBSs, the greek letters in red color identify the number of photons observed at each of the detectors and $|0\rangle$ denotes the vacuum state.

channel loss is given by $\Lambda_{\max} = \max\{\Lambda \in \mathbb{R}^+ | K_\infty \geq 0\} \approx 0.7$dB, where K_∞ is the asymptotic secret key rate in the absence of a qubit amplifier,

$$K_\infty = 1 - h\left[\frac{1}{2} + \frac{1}{2}\sqrt{16\omega(\omega-1)+3}\right] - h(Q). \tag{58}$$

If we assume, for instance, an attenuation coefficient of, say, $\alpha = 0.2$ dB/km, which corresponds to the typical value for single-mode fibers in the telecom wavelength, then $\Lambda_{\max} = 0.7$ dB means that the maximum achievable distance between the parties in the DIQKD link is $L_{\max} = 3.5$ km.

Let us now consider the case where ρ_{ab} is a more general entangled state with arbitrary photon-number statistics, $\rho_{ab} = |\psi\rangle_{ab}\langle\psi|$ as described by Eqs. (5) and (4). Again, this is equivalent to considering the mixed states σ_{ab} given in Eq. (3) due to the use of PNR detectors. Then, by using the same techniques employed there, it is possible to derive the click pattern distribution $\mathrm{P}\left(\alpha,\beta,\gamma,\delta|n\right)_{\theta_{\rm A},\theta_{\rm B}}$ that matches the setup of Fig. 6, conditioned on the number of photon pairs n emitted by the source. Precisely, one finds

$$\mathrm{P}\left(\alpha,\beta,\gamma,\delta|n\right)_{\theta_{\rm A},\theta_{\rm B}} = \delta^n_{\alpha+\beta}\tilde{\mathrm{P}}\left(\alpha,\beta,\gamma,\delta|n\right)_{\theta_{\rm A},\theta_{\rm B}}, \tag{59}$$

where

$$\begin{aligned}\tilde{\mathrm{P}}\left(\alpha,\beta,\gamma,\delta|n\right)_{\theta_{\rm A},\theta_{\rm B}} &= \frac{\alpha!\beta!\gamma!\delta!}{n+1}\eta_{\rm ch}^{\gamma+\delta}(1-\eta_{\rm ch})^{n-\gamma-\delta}\cos\theta_{\rm A}{}^{2\alpha}\sin\theta_{\rm A}{}^{2(n-\alpha)}\cos\theta_{\rm B}{}^{2\gamma}\sin\theta_{\rm B}{}^{2\delta}\sum_{j=0}^{n}\sum_{\Delta=-j}^{n-j}\sum_{l=\max\{0,\gamma+\delta-j,\Delta\}}^{\min\{n-j,\gamma+\delta,\gamma+\delta+\Delta\}}\\ &\times\sum_{h=\max\{0,\alpha+j-n\}}^{\min\{\alpha,j\}}\sum_{H=\max\{0,\alpha+j+\Delta-n\}}^{\min\{\alpha,j+\Delta\}}\sum_{s=\max\{0,l-\delta\}}^{\min\{\gamma,l\}}\sum_{S=\max\{0,l-\delta-\Delta\}}^{\min\{\gamma,l-\Delta\}}(-1)^{h+H+s+S+\Delta}\left(\frac{\sin\theta_{\rm A}}{\cos\theta_{\rm A}}\right)^{2(h+H-j)-\Delta}\\ &\times\left(\frac{\sin\theta_{\rm B}}{\cos\theta_{\rm B}}\right)^{2(s+S-l)+\Delta}\Upsilon(n,j,l,h,H,s,S,\alpha,\beta,\gamma,\delta,\Delta),\end{aligned} \tag{60}$$

with the parameter $\Upsilon(n,j,l,h,H,s,S,\alpha,\beta,\gamma,\delta,\Delta)$ being of the form

$$\begin{aligned}\Upsilon(n,j,l,h,H,s,S,\alpha,\beta,\gamma,\delta,\Delta) &= \binom{n-j}{\alpha-h}\binom{n-j-\Delta}{\alpha-H}\binom{j}{h}\binom{j+\Delta}{H}\left[(j+l-\gamma-\delta)!(n-j-l)!(\gamma-s)!(\gamma-S)!\right]^{-1}\\ &\times\left[(s+\delta-l)!(S+\delta+\Delta-l)!s!S!(l-s)!(l-\Delta-S)!\right]^{-1}.\end{aligned} \tag{61}$$

Summing over n and taking into account the $\delta^n_{\alpha+\beta}$ factor, we obtain that the overall distribution reads

$$\mathrm{P}\left(\alpha,\beta,\gamma,\delta\right)_{\theta_{\rm A},\theta_{\rm B}} = \sum_{n=0}^{\infty}p_n\mathrm{P}\left(\alpha,\beta,\gamma,\delta|n\right)_{\theta_{\rm A},\theta_{\rm B}} = p_{\alpha+\beta}\tilde{\mathrm{P}}\left(\alpha,\beta,\gamma,\delta|\alpha+\beta\right)_{\theta_{\rm A},\theta_{\rm B}}. \tag{62}$$

As in App. I A 1, a permutation step is required to enforce correlation between Alice's and Bob's outcomes. Therefore, we define

$$\tilde{\mathrm{p}}\left(\alpha,\beta,\gamma,\delta\right)_{\theta_{\rm A},\theta_{\rm B}} = \mathrm{P}\left(\beta,\alpha,\gamma,\delta\right)_{\theta_{\rm A},\theta_{\rm B}}. \tag{63}$$

Given $\tilde{\mathrm{p}}\left(\alpha,\beta,\gamma,\delta\right)_{\theta_{\rm A},\theta_{\rm B}}$, one can readily define the post-processed click pattern distribution $\mathbf{P}(A_{\rm A},A_{\rm B})_{\theta_{\rm A},\theta_{\rm B}}$ by simply summing over all click patterns $(\alpha,\beta,\gamma,\delta)$ that are mapped to a specific pair of deterministic assignments, $(A_{\rm A},A_{\rm B})$,

as we are assuming here that $p_{\rm d}=0$. Once this is done, it is straightforward to define the parameters of the honest implementation. In particular, we have that

$$Q = \mathbf{P}(0,1)_{0,0} + \mathbf{P}(1,0)_{0,0}. \tag{64}$$

On the other hand, $\omega = S/8 + 1/2$, where in this case the CHSH violation reads $S = E_{0,-\frac{\pi}{8}} + E_{0,\frac{\pi}{8}} - E_{\frac{\pi}{4},-\frac{\pi}{8}} + E_{\frac{\pi}{4},\frac{\pi}{8}}$ and

$$E_{\theta_{\rm A},\theta_{\rm B}} = 2\left[\mathbf{P}(0,0)_{\theta_{\rm A},\theta_{\rm B}} + \mathbf{P}(1,1)_{\theta_{\rm A},\theta_{\rm B}}\right] - 1. \tag{65}$$

In doing so, one can numerically compute the maximum tolerated channel loss for any given photon-number statistics p_n. For instance, in the case of PDC sources, the statistics read $p_n = (n+1)\lambda^n(1+\lambda)^{-n-2}$, so that $\Lambda_{\rm max} = \max\{\Lambda \in \mathbb{R}^+ | K_\infty \geq 0\}$. In this scenario, however, the definition of K_∞ includes a maximization over the free parameter λ characterizing the intensity of the PDC source, *i.e.*,

$$K_\infty = \max_{\lambda\in\mathbb{R}^+}\left\{1 - h\left[\frac{1}{2} + \frac{1}{2}\sqrt{16\omega(\omega-1)+3}\right] - h(Q)\right\}. \tag{66}$$

In this way, we find $\Lambda_{\rm max} \approx 0.4$ dB, which results in a maximum transmission distance $L_{\rm max} \approx 2$ km for an attenuation coefficient $\alpha = 0.2$ dB/km.

E. DIQKD with two qubit amplifiers

Finally, in this Appendix we consider a different DIQKD setup from that presented in the main text, *i.e.*, we now assume that the entanglement source ρ_{ab} is located in the middle of the channel, equidistant from Alice's and Bob's labs. Also, we suppose that both parties hold an ESR-based qubit amplifier to palliate the effect of the channel loss (the case with two PQAs is briefly discussed afterwards also below).

The goal is to investigate whether or not such setup could increase the maximum transmission distance before the secret key rate sharply drops to zero. Intuitively speaking, the cutoff point where the secret key rate drops down to zero happens at the range of distances for which a significant fraction of the successful heralding events at the qubit amplifier are triggered by the dark counts of the detectors. In this scenario, the conditional quantum state shared by Alice and Bob after a successful heralding takes place is a separable state, thus leading to a vanishing conditional secret key rate. In the setup with a central source ρ_{ab} and two qubit amplifiers, the input signal to each qubit amplifier has only traveled a half of the overall transmission distance, so the probability of still carrying a photon that hits a detector within the amplifier may still be large compared to that of a dark count, and this could lead to an enhancement of the transmission distance. Of course, one also expects that the overall secret key rate decreases, as the probability to have a simultaneous successful heralding event at both qubit amplifiers is lower than that of a single success in a unique qubit amplifier, as required in the original setup. Nevertheless, compared to that setup, it takes longer distances for the conditional secret key rate to sharply drop down to zero, since it comes from an entangled state with a higher probability. As a result, the cutoff point is shifted to further distances.

For instance, in the case where ρ_{ab} is an ideal entanglement source, the setup with two qubit amplifiers roughly doubles the maximum distance without significantly affecting the secret key rate. This is illustrated by the bluish lines in Fig. 7, where we compare the secret key rate with one and two qubit amplifiers as a function of the channel loss. Here, as in the previous examples, we set the dark count rate to $p_{\rm d} = 10^{-7}$. Also, we assume the set S_1 of security settings presented in the main text, and we use the same values of $\eta_{\rm c,d}$ employed there. From Fig. 7 we have that, if ideal entanglement sources were available, it would actually be beneficial to use two ESRs for long-distance transmissions. Similar conclusions would be obtained if two PQAs were used instead.

However, this might not be the case if one considers practical light sources with a nonzero probability of emitting multi-photon pulses. For example, in the case of PDC sources, it turns out that the use of two qubit amplifiers does not seem to improve the performance of the system in a practical regime. This is exemplified by the yellowish lines in Fig. 7. Precisely, Fig. 7(a) shows that no advantage is obtained with two qubit amplifiers in a practical key rate regime in this scenario. Arguably, one would expect to see an advantage by considering lower detection and coupling efficiencies or higher dark count rates, as in both situations the cutoff point where the key rate sharply drops to zero is shifted to lower distances. Nevertheless, these two scenarios are notably restricted by time considerations. To see this, in Fig. 7(b) we plot the average number of transmitted signals $\langle N\rangle$ required to achieve the secret key rates of Fig. 7(a), comparing again the cases of one and two ESRs. As expected, using two qubit amplifiers instead

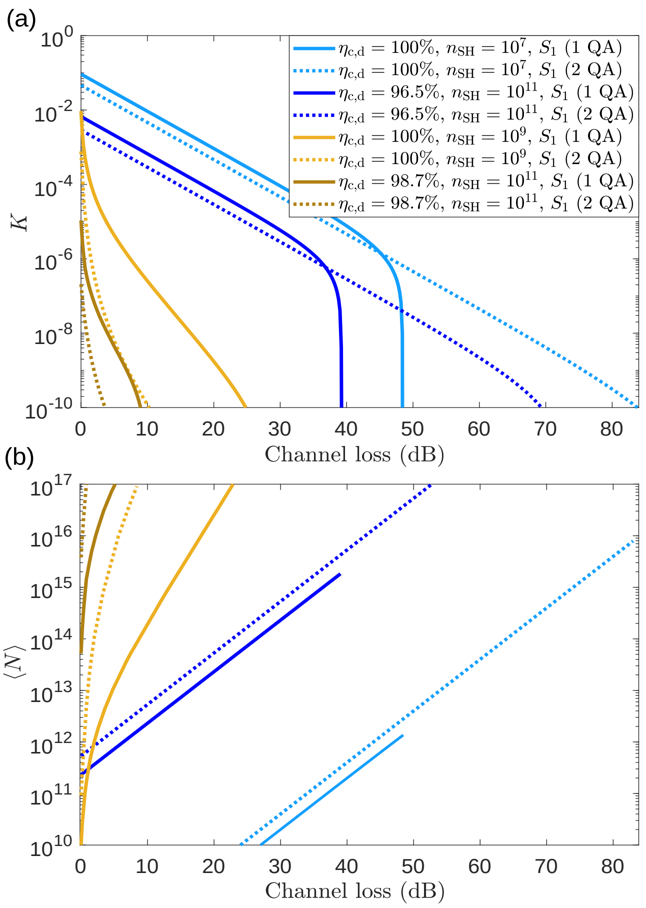

FIG. 7: Comparison between the performance of the DIQKD scheme with one and two ESR-based qubit amplifiers for ideal sources (bluish lines) and for PDC sources (yellowish lines). In both cases, solid (dotted) lines are used for the case with one (two) qubit amplifiers. For illustration purposes, here we consider the same combinations of parameters $\eta_{\rm c,d}$ and $n_{\rm SH}$ that were already used in the main text and in App. I C. Also, we suppose the less demanding set of security requirements introduced in the main text, given by S_1. (a) Secret key rate as a function of the channel loss. (b) Average number of transmitted signals as a function of the channel loss.

of one demands longer DIQKD sessions in order to harvest a specific block size, due to the requirement of having simultaneous successful heralding events. In fact, even for $\eta_{\rm c,d}=100\%$ and $n_{\rm SH}=10^9$, the necessary value of $\langle N\rangle$ is larger than 10^{15} for a channel loss as low as $\Lambda\approx 4$ dB, and any lower value of the detection and coupling efficiencies would result in smaller values of Λ. This being the case, even if one considers higher values of the dark count rate $p_{\rm d}$, no advantage is expected from the setup with two qubit amplifiers within such a short channel loss interval (before the duration of the DIQKD session becomes impractical).

Indeed, if one considers two PQAs instead of two ESRs and compares again this setup with the one that uses a single PQA, the time constraint with PDC sources becomes even more strict, as each PQA additionally includes two triggered single-photon sources that must yield a success in their idler mode in order not to dismiss a detection event. That is, a setup with two PQAs requires the trigger of four single-photon sources and two simultaneous successful BSMs afterwards. As a result, this overall decrease of the success probability (which is particularly relevant for PDC sources) translates into larger values of $\langle N\rangle$ which seem to render this solution impractical.

In short, the potential advantage of using two qubit amplifiers to enhance further the distance covered with DIQKD strongly depends on the photon-number statistics of the entanglement sources under consideration. In this sense, although Fig. 7 is restricted to ideal sources and PDC sources, we include below all the necessary calculations to evaluate the case of general photon-number statistics, maintaining the form of the states given in Eqs. (3) and (4). In this regard, we remark that reducing the probability to emit multiple photon pulses with respect to that of PDC sources, one could approach the behaviour of ideal sources shown in Fig. 7.

1. Click pattern distribution

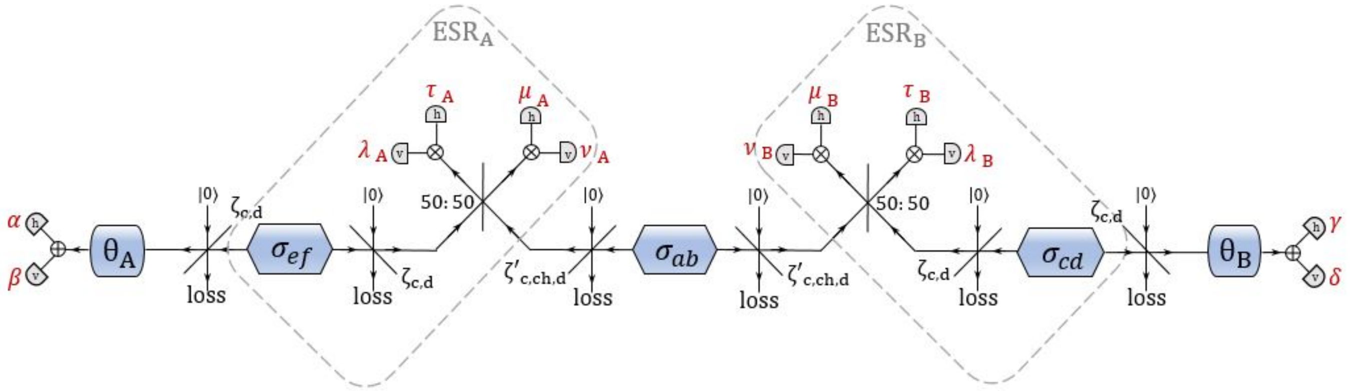

FIG. 8: Schematic of the DIQKD protocol with a central source and two ESR-based qubit amplifiers. The central source is denoted by σ_{ab}, while σ_{ef} (σ_{cd}) stands for the source within the ESR held by Alice (Bob). As usual, θ_A (θ_B) denotes the rotation angle of Alice's (Bob's) measurement settings and $\zeta_\mathrm{c,d}$ and $\zeta_\mathrm{c,ch,d}$ tag the effective efficiency parameters, $\zeta_\mathrm{c,d}=\eta_\mathrm{c}\eta_\mathrm{d}$ and $\zeta_\mathrm{c,ch,d}=\eta_\mathrm{c}\eta_\mathrm{ch}\eta_\mathrm{d}$, where η_c, η_d and η_ch denote the transmittance of the BSs modeling the coupling loss, the detection loss and the channel loss, respectively. The symbol "$\oplus$" is used again to indicate the PBSs, the greek letters in red color tag the number of photons observed at each of the detectors and $|0\rangle$ stands for the vacuum state. Two dashed grey rectangles identify the two ESRs, ESR$_\mathrm{A}$ and ESR$_\mathrm{B}$.

A schematic of the DIQKD setup just discussed, with a central source and assisted by two ESR-based qubit amplifiers, is shown in Fig. 8. Using the same techniques employed in App. I A and App. I B, it is possible to derive the click pattern distribution that matches the setup of Fig. 8, conditioned on the numbers of photon pairs n_1, n_2 and n_3 emitted by the entanglement sources σ_{ab}, σ_{ef} and σ_{cd}, respectively. Precisely, let us define the click pattern (in vector notation) as $\vec{\alpha}=(\alpha,\beta,\gamma,\delta,\mu_\mathrm{A},\nu_\mathrm{A},\tau_\mathrm{A},\lambda_\mathrm{A},\mu_\mathrm{B},\nu_\mathrm{B},\tau_\mathrm{B},\lambda_\mathrm{B})$, where μ_A, ν_A, τ_A and λ_A (μ_B, ν_B, τ_B and λ_B) are the numbers of photons recorded within Alice's (Bob's) ESR in any given detection event, while α and β (γ and δ) denote again the numbers of photons she (he) observes with the detectors in her (his) lab when performing a measurement with rotation angle θ_A (θ_B). In particular, one finds that $\mathrm{P}\left(\vec{\alpha}|n_1,n_2,n_3\right)_{\theta_\mathrm{A},\theta_\mathrm{B}}$ reads

$$
\begin{aligned}
&\mathrm{P}\left(\vec{\alpha}|n_1,n_2,n_3\right)_{\theta_\mathrm{A},\theta_\mathrm{B}}=\frac{\alpha!\beta!\gamma!\delta!\mu_\mathrm{A}!\nu_\mathrm{A}!\tau_\mathrm{A}!\lambda_\mathrm{A}!\mu_\mathrm{B}!\nu_\mathrm{B}!\tau_\mathrm{B}!\lambda_\mathrm{B}!}{(n_1+1)(n_2+1)(n_3+1)2^{\mu_\mathrm{A}+\nu_\mathrm{A}+\tau_\mathrm{A}+\lambda_\mathrm{A}+\mu_\mathrm{B}+\nu_\mathrm{B}+\tau_\mathrm{B}+\lambda_\mathrm{B}}}\zeta_\mathrm{c,d}^{\alpha+\beta+\gamma+\delta+\mu_\mathrm{A}+\nu_\mathrm{A}+\tau_\mathrm{A}+\lambda_\mathrm{A}+\mu_\mathrm{B}+\nu_\mathrm{B}+\tau_\mathrm{B}+\lambda_\mathrm{B}}\\
&\times(1-\zeta_\mathrm{c,d})^{2(n_2+n_3)-\alpha-\beta-\gamma-\delta-\mu_\mathrm{A}-\nu_\mathrm{A}-\tau_\mathrm{A}-\lambda_\mathrm{A}-\mu_\mathrm{B}-\nu_\mathrm{B}-\tau_\mathrm{B}-\lambda_\mathrm{B}}(1-\zeta_\mathrm{c,ch,d})^{2n_1}\cos\theta_\mathrm{A}{}^{-2\beta}\sin\theta_\mathrm{A}{}^{2(\alpha+2\beta)}\cos\theta_\mathrm{B}{}^{-2\delta}\sin\theta_\mathrm{B}{}^{2(\gamma+2\delta)}\\
&\times\sum_{i_1=0}^{n_1}\sum_{i_2=0}^{n_2}\sum_{i_3=0}^{n_3}\sum_{\Delta=\max\{-i_1,i_2-n_2,-i_3\}}^{\min\{n_1-i_1,i_2,n_3-i_3\}}\sum_{j_1=\max\{0,-\Delta,\nu_\mathrm{A}+\lambda_\mathrm{A}-i_2\}}^{\min\{i_1,\nu_\mathrm{A}+\lambda_\mathrm{A},\nu_\mathrm{A}+\lambda_\mathrm{A}-\Delta\}}\sum_{j_1'=\max\{0,-\Delta,\mu_\mathrm{B}+\tau_\mathrm{B}+i_3-n_3\}}^{\min\{i_1,\mu_\mathrm{B}+\tau_\mathrm{B},\mu_\mathrm{B}+\tau_\mathrm{B}-\Delta\}}\sum_{j_2'=\max\{0,\Delta,\alpha+\beta+i_2-n_2\}}^{\min\{i_2,\alpha+\beta,\alpha+\beta+\Delta\}}\\
&\times\sum_{j_3'=\max\{0,-\Delta,\gamma+\delta+i_3-n_3\}}^{\min\{i_3,\gamma+\delta,\gamma+\delta-\Delta\}}\sum_{l=\max\{0,\Delta,\mu_\mathrm{A}+\tau_\mathrm{A}+i_2-n_2\}}^{\min\{n_1-i_1,\mu_\mathrm{A}+\tau_\mathrm{A},\mu_\mathrm{A}+\tau_\mathrm{A}+\Delta\}}\sum_{l'=\max\{0,\Delta,\nu_\mathrm{B}+\lambda_\mathrm{B}-i_3\}}^{\min\{n_1-i_1,\nu_\mathrm{B}+\lambda_\mathrm{B},\nu_\mathrm{B}+\lambda_\mathrm{B}+\Delta\}}\sum_{k=\max\{0,l-\tau_\mathrm{A}\}}^{\min\{l,\mu_\mathrm{A}\}}\sum_{K=\max\{0,l-\tau_\mathrm{A}-\Delta\}}^{\min\{l-\Delta,\mu_\mathrm{A}\}}\\
&\times\sum_{m=\max\{0,j_1-\lambda_\mathrm{A}\}}^{\min\{j_1,\nu_\mathrm{A}\}}\sum_{M=\max\{0,j_1+\Delta-\lambda_\mathrm{A}\}}^{\min\{j_1+\Delta,\nu_\mathrm{A}\}}\sum_{p=\max\{0,j_1'-\tau_\mathrm{B}\}}^{\min\{j_1',\mu_\mathrm{B}\}}\sum_{P=\max\{0,j_1'+\Delta-\tau_\mathrm{B}\}}^{\min\{j_1'+\Delta,\mu_\mathrm{B}\}}\sum_{q=\max\{0,l'-\lambda_\mathrm{B}\}}^{\min\{l',\nu_\mathrm{B}\}}\sum_{Q=\max\{0,l'-\Delta-\lambda_\mathrm{B}\}}^{\min\{l'-\Delta,\nu_\mathrm{B}\}}\sum_{s=\max\{0,\beta-j_2'\}}^{\min\{\beta,\alpha+\beta-j_2'\}}\\
&\times\sum_{S=\max\{0,\beta-j_2'+\Delta\}}^{\min\{\beta,\alpha+\beta-j_2'+\Delta\}}\sum_{z=\max\{0,\delta-j_3'\}}^{\min\{\delta,\gamma+\delta-j_3'\}}\sum_{Z=\max\{0,\delta-j_3'-\Delta\}}^{\min\{\delta,\gamma+\delta-j_3'-\Delta\}}(-1)^{k+K+m+M+p+P+q+Q+s+S+z+Z+\Delta}\left[\frac{\zeta_\mathrm{c,ch,d}(1-\zeta_\mathrm{c,d})}{\zeta_\mathrm{c,d}(1-\zeta_\mathrm{c,ch,d})}\right]^{j_1+l+j_1'+l'}\\
&\times\left(\frac{\sin\theta_\mathrm{A}}{\cos\theta_\mathrm{A}}\right)^{\Delta-2(j_2'+s+S)}\left(\frac{\sin\theta_\mathrm{B}}{\cos\theta_\mathrm{B}}\right)^{-2(j_3'+z+Z)-\Delta}\\
&\times\Upsilon(n_1,n_2,n_3,i_1,i_2,i_3,j_1,j_1',j_2',j_3',l,l',k,K,m,M,p,P,q,Q,s,S,z,Z,\alpha,\beta,\gamma,\delta,\mu_\mathrm{A},\nu_\mathrm{A},\tau_\mathrm{A},\lambda_\mathrm{A},\mu_\mathrm{B},\nu_\mathrm{B},\tau_\mathrm{B},\lambda_\mathrm{B},\Delta),
\end{aligned}
\tag{67}
$$

with

$$\Upsilon(n_1,n_2,n_3,i_1,i_2,i_3,j_1,j_1',j_2',j_3',l,l',k,K,m,M,p,P,q,Q,s,S,z,Z,\alpha,\beta,\gamma,\delta,\mu_\mathrm{A},\nu_\mathrm{A},\tau_\mathrm{A},\lambda_\mathrm{A},\mu_\mathrm{B},\nu_\mathrm{B},\tau_\mathrm{B},\lambda_\mathrm{B},\Delta) =$$
$$\frac{i_1!(n_1-i_1)!(i_1+\Delta)!(n_1-i_1-\Delta)!i_2!(n_2-i_2)!(i_2-\Delta)!(n_2-i_2+\Delta)!i_3!(n_3-i_3)!(i_3+\Delta)!(n_3-i_3-\Delta)!}{(i_1-j_1')!(i_1-j_1)!(n_1-i_1-l)!(n_1-i_1-l')!(i_2-\nu_\mathrm{A}-\lambda_\mathrm{A}+j_1)!(n_2-i_2-\mu_\mathrm{A}-\tau_\mathrm{A}+l)!(n_2-i_2-\alpha-\beta+j_2')!}$$
$$\times\frac{[(i_3-j_3')!(n_3-i_3-\mu_\mathrm{B}-\tau_\mathrm{B}+j_1')!(n_3-i_3-\gamma-\delta+j_3')!(i_3-\nu_\mathrm{B}-\lambda_\mathrm{B}+l')!(\delta-z)!(\delta-Z)!z!Z!(j_3'-\delta+z)!S!]^{-1}}{(i_2-j_2')!(j_3'+\Delta-\delta+Z)!(\gamma+\delta-j_3'-z)!(\gamma+\delta-j_3'-\Delta-Z)!(\beta-s)!(\beta-S)!(j_2'-\beta+s)!(j_2'-\Delta-\beta+S)!s!}$$
$$\times\frac{[(\alpha+\beta-j_2'-s)!(\alpha+\beta-j_2'+\Delta-S)!k!K!(l-k)!(l-\Delta-K)!(\mu_\mathrm{A}-k)!(\mu_\mathrm{A}-K)!(\tau_\mathrm{A}-l+k)!]^{-1}}{m!M!(j_1-m)!(j_1+\Delta-M)!(\nu_\mathrm{A}-m)!(\nu_\mathrm{A}-M)!(\lambda_\mathrm{A}-j_1+m)!(\lambda_\mathrm{A}-j_1-\Delta+M)!p!P!(j_1'-p)!(j_1'+\Delta-P)!}$$
$$\times[(\tau_\mathrm{A}-l+\Delta+K)!(\mu_\mathrm{B}-P)!(\tau_\mathrm{B}-j_1'+p)!(\tau_\mathrm{B}-j_1'-\Delta+P)!q!Q!(l'-q)!(l'-\Delta-Q)!(\nu_\mathrm{B}-q)!(\nu_\mathrm{B}-Q)!]^{-1}$$
$$\times[(\mu_\mathrm{B}-p)!(\lambda_\mathrm{B}-l'+q)!(\lambda_\mathrm{B}-l'+\Delta+Q)!]^{-1}. \tag{68}$$

The normalization condition $\sum_{\vec{\alpha}}\mathrm{P}\left(\vec{\alpha}|n_1,n_2,n_3\right)_{\theta_\mathrm{A},\theta_\mathrm{B}}=1$ holds for any set of physical parameters θ_A, θ_B, $\zeta_\mathrm{c,d}$, and $\zeta_\mathrm{c,ch,d}$, and only those click patterns $\vec{\alpha}$ such that $\alpha+\beta\leq n_2$, $\gamma+\delta\leq n_3$, $\mu_\mathrm{A}+\nu_\mathrm{A}+\tau_\mathrm{A}+\lambda_\mathrm{A}\leq n_1+n_2$ and $\mu_\mathrm{B}+\nu_\mathrm{B}+\tau_\mathrm{B}+\lambda_\mathrm{B}\leq n_1+n_3$ have a nonzero probability to happen, since we have disregarded dark counts so far. If we incorporate now the noise model introduced in the main text, the click pattern distribution in the noisy scenario becomes

$$\tilde{\mathrm{P}}\left(\vec{\alpha}|n_1,n_2,n_3\right)_{\theta_\mathrm{A},\theta_\mathrm{B}}=(1-12p_\mathrm{d})\mathrm{P}\left(\vec{\alpha}|n_1,n_2,n_3\right)_{\theta_\mathrm{A},\theta_\mathrm{B}}+p_\mathrm{d}\sum_{\vec{\sigma}\in\Gamma_{\vec{\alpha}}}\mathrm{P}\left(\vec{\sigma}|n_1,n_2,n_3\right)_{\theta_\mathrm{A},\theta_\mathrm{B}}+O(p_\mathrm{d}^2), \tag{69}$$

where, again, $\Gamma_{\vec{\alpha}}=\{\vec{\sigma}:|\vec{\alpha}|=|\vec{\sigma}|+1\}$ and, for an arbitrary $\vec{\alpha}$, $|\vec{\alpha}|=\alpha+\beta+\gamma+\delta+\mu_\mathrm{A}+\nu_\mathrm{A}+\tau_\mathrm{A}+\lambda_\mathrm{A}+\mu_\mathrm{B}+\nu_\mathrm{B}+\tau_\mathrm{B}+\lambda_\mathrm{B}$. Coming next, by defining

$$\tilde{\mathrm{p}}\left(\vec{\alpha}|n_1,n_2,n_3\right)_{\theta_\mathrm{A},\theta_\mathrm{B}}=\tilde{\mathrm{P}}_{\alpha\leftrightarrow\beta}\left(\vec{\alpha}|n_1,n_2,n_3\right)_{\theta_\mathrm{A},\theta_\mathrm{B}}, \tag{70}$$

we flip Alice's outcomes to enforce direct correlation with Bob's, and by summing over all click patterns $\vec{\alpha}$ that are mapped to the pair of deterministic assignments $(A_\mathrm{A},A_\mathrm{B})$, we finally obtain the post-processed click pattern distribution, $\mathbf{P}(A_{\vec{\alpha}}|n_1,n_2,n_3)_{\theta_\mathrm{A},\theta_\mathrm{B}}$. In accordance with App. I A 1 and App. I B 2, $A_{\vec{\alpha}}$ is defined as $A_{\vec{\alpha}}=(A_\mathrm{A},A_\mathrm{B},\mu_\mathrm{A},\nu_\mathrm{A},\tau_\mathrm{A},\lambda_\mathrm{A},\mu_\mathrm{B},\nu_\mathrm{B},\tau_\mathrm{B},\lambda_\mathrm{B})$. Note that if we sum over all possible photon numbers n_1, n_2 and n_3 and assume arbitrary statistics for the entanglement sources, say p_{n_1}, p'_{n_2} and p''_{n_3}, we obtain the overall distribution

$$\mathbf{P}(A_{\vec{\alpha}})_{\theta_\mathrm{A},\theta_\mathrm{B}}=\sum_{n_1,n_2,n_3}p_{n_1}p'_{n_2}p''_{n_3}\mathbf{P}(A_{\vec{\alpha}}|n_1,n_2,n_3)_{\theta_\mathrm{A},\theta_\mathrm{B}}. \tag{71}$$

2. *Parameters P_SH, $Q|_\mathrm{SH}$ and $\omega|_\mathrm{SH}$*

In contrast to the setup with a single qubit amplifier, two BSMs are performed in the current scenario, one per ESR (see Fig. 8. Therefore, given that four different click patterns are considered to be successful heralding events in each qubit amplifier, there exist sixteen events that are not discarded by either Alice or Bob, corresponding to those cases for which both $(\mu_\mathrm{A},\nu_\mathrm{A},\tau_\mathrm{A},\lambda_\mathrm{A})$ and $(\mu_\mathrm{B},\nu_\mathrm{B},\tau_\mathrm{B},\lambda_\mathrm{B})$ belong to the set $\{(1,1,0,0),(0,1,1,0),(1,0,0,1),(0,0,1,1)\}$. Let Ω be one of them, say,

$$\Omega=\big\{(\mu_\mathrm{A},\nu_\mathrm{A},\tau_\mathrm{A},\lambda_\mathrm{A},\mu_\mathrm{B},\nu_\mathrm{B},\tau_\mathrm{B},\lambda_\mathrm{B})=(1,1,0,0,1,1,0,0)\big\}. \tag{72}$$

Then, due to the symmetries of the channel model, it turns out that $P_\mathrm{SH}=16P_\Omega$, $\omega|_\mathrm{SH}=\omega|_\Omega$ and $Q|_\mathrm{SH}=Q|_\Omega$. As in App. I A 2, P_Ω can be computed as

$$P_\Omega=\sum_{A_\mathrm{A},A_\mathrm{B}}\mathbf{P}(A_\mathrm{A},A_\mathrm{B},\Omega)_{0,0}, \tag{73}$$

with $A_\mathrm{A},A_\mathrm{B}\in\{0,1\}$. Analogously,

$$Q|_\Omega=\frac{1}{P_\Omega}\left[\mathbf{P}(0,1,\Omega)_{0,0}+\mathbf{P}(1,0,\Omega)_{0,0}\right], \tag{74}$$

and $\omega|_\Omega = S|_\Omega/8 + 1/2$ with the conditional CHSH violation given by

$$S|_\Omega = E_{0,-\frac{\pi}{8}}|_\Omega + E_{0,\frac{\pi}{8}}|_\Omega - E_{\frac{\pi}{4},-\frac{\pi}{8}}|_\Omega + E_{\frac{\pi}{4},\frac{\pi}{8}}|_\Omega, \tag{75}$$

being

$$E_{\theta_\mathrm{A},\theta_\mathrm{B}}|_\Omega = \frac{2}{P_\Omega}[\mathbf{P}(0,0,\Omega)_{\theta_\mathrm{A},\theta_\mathrm{B}} + \mathbf{P}(1,1,\Omega)_{\theta_\mathrm{A},\theta_\mathrm{B}}] - 1. \tag{76}$$

Note that, with our choice of Ω, it is the third summand that carries the minus sign in Eq. (75).

---

[1] Curty, M., Moroder, T. (2011). Heralded-qubit amplifiers for practical device-independent quantum key distribution. Physical Review A 84, 010304.

[2] For all $j \in \mathbb{Z}$, $\Theta_j = 1$ if $j \geq 0$ and $\Theta_j = 0$ otherwise.

[3] Actually, the summand that carries the minus sign in the definition of $S|_\Omega$ depends on the successful heralding event considered. Indeed, two out of the four successful heralding events require the definition of $S|_\Omega$ given by Eq. (29), while the other two require the minus sign to be carried by the third summand in Eq. (29). This is so because the quantum measurements that lead to a maximum violation of the CHSH inequality are different for each Bell pair, and one can equivalently account for this fact by changing the definition of $S|_\Omega$ depending on the Bell pair.

[4] Again, also here two successful heralding events require the given definition of $S|_{\Omega,\text{double trigger}}$, while the other two require the minus sign to be carried by the fourth summand in Eq. (55).
